# Supplementary material for: Spurious precision in meta-analysis of observational research
Source: Nat Commun. 2025 Sep 26;16:8454. doi: 10.1038/s41467-025-63261-0 (PMC12475282; doi:10.1038/s41467-025-63261-0)
Supplement: Supplementary file 1 — Supplementary Information [file 41467_2025_63261_MOESM1_ESM.pdf]

Supplementary Information for

Spurious Precision in Meta-Analysis of Observational Research

Zuzana Irsova*, Pedro R. D. Bom, Tomas Havranek, Heiko Rachinger

*Corresponding author: Zuzana Irsova, zuzana.irsova@ies-prague.org

This PDF file includes:

Supplementary Discussion: Simulations

Supplementary Discussion: Empirical Applications

Supplementary Methods: Additional Simulations

Figs. S1 to S29

Tables S1 to S9

References

S1 Supplementary Discussion: Simulations

S1.1 Benchmark Estimators

We select 7 estimators, described in Table 1 of the main text, to illustrate the impact of spurious precision. The estimators represent the breadth of meta-analysis approaches. We always try to include the latest method that corresponds to a particular line of research and has already been examined by simulations and applications. Regarding selection models, many different versions exist. Perhaps the one most commonly used is the 3PSM model [1,2] described in detail, for example, by Pustejovsky and Rodgers. [3,4] We use the Andrews and Kasy [5] selection model, which is very similar to 3PSM but has a different optimization algorithm for maximum likelihood. Kvarven et al. [6] suggest that the Andrews and Kasy model works slightly better than the 3PSM model when compared to preregistered multilab replications. Hong and Reed [7] show that the Andrews and Kasy model performs well in simulations. However, if the 3PSM model was used instead, our results would remain qualitatively similar.

One promising family of models that we do not consider is the one introduced by Mathur and VanderWeele. [8,9] We do not include these models because they are not bias-correction methods but sensitivity tests for meta-analysis: for example, they allow meta-analysts to compute how large publication bias must be to turn the mean reported result insignificant. Recently, Maya Mathur has presented a new technique that corrects for p-hacking. [10] The technique focuses on insignificant estimates, which are assumed to be unaffected by publication bias and p-hacking. If the assumption holds and if many insignificant estimates are reported, one can use Bayesian techniques to recover the underlying distribution of studies. Note that the assumptions of this technique are perfectly aligned with the setup of our simulation exercise, so in our simulations Mathur's technique would correct for all the bias due to p-hacking by definition. While not eliminating the bias perfectly, MAIVE is more flexible since it does not require insignificant reported estimates and does not assume that all insignificant estimates are unbiased. A different simulation framework and empirical applications will be needed to compare the performance of MAIVE and Mathur's brand new technique.

The simple average is the only examined estimator unaffected by precision. The FE/WLS estimator is the inverse-variance weighted version of the simple average. One could add random effects as another estimator, but since there the weight is diluted by the heterogeneity term, the results would always lie between the simple average and FE. A worry about spurious precision will reinforce the case of random effects versus unadjusted FE as a summary statistic, a choice otherwise depending on the nature of heterogeneity. [11,12] Note that in Appendix S3 we simulate a scenario with true effect heterogeneity as assumed by the random effects model.

The simple average and FE/WLS are summary statistics not meant to correct biases—though researchers note that FE/WLS helps attenuate publication bias.[13–15] Of the 5 bias-correcting estimators, 3 are based on the funnel plot (PET-PEESE, EK, WAAP), and 2 are selection models (Andrews and Kasy, p-uniform*). PET-PEESE is a classical funnel technique and can be understood as an extension of the Egger regression; it was shown to work well relative to other estimators when compared to preregistered replications.[16] Regressions based on the funnel plot effectively invoke inverse-variance weighting twice: first as an explicit weight, second implicitly via the search for the mean effect conditional on maximum precision. Intuitively, such an approach will be highly sensitive to spurious precision. EK is a model that can be understood as a hybrid of funnel-based and selection model approaches[8] because it estimates a threshold for which estimates are selected no more. Since it follows funnel plot intuition and depends on the same assumptions, we group it together with funnel-based techniques. EK is the technique theoretically most dependent on correctly reported precision: the standard error is used as a weight, regressor, and identification threshold.

The last funnel-based technique, WAAP, is based on FE/WLS, but also uses the standard error to exclude studies that have low power (indirectly, that are too imprecise). We understand WAAP as another way to estimate the top of the funnel plot, the mean study implied by maximum precision. So WAAP, EK, and PET-PEESE share the same underlying assumptions: 1) In the absence of publication bias, there is no correlation between estimates and standard errors. 2) If there is publication bias, it works on the reported effect size, not the standard error. The standard error is exogenous (given to the researcher) and more precise (less noisy) estimates are less biased, as in the Lombard effect analogy laid out in Section of the main text. Note that, in addition to publication bias, the funnel-based techniques also allow for p-hacking of effect sizes—if the p-hacking is a direct response to imprecision.

The selection models we consider, Andrews and Kasy and p-uniform*, allow for joint selection on both estimates and standard errors, as long as the final selection criterion is the p-value. Andrews and Kasy is the latest selection model in the tradition of Hedges, while the p-uniform* is the latest version of selection model simplifications that are more parsimonious but less flexible. Even more flexible selection models exist that allow for separate selection on estimates and standard errors, irrespective of the p-value,[17–19] but these are difficult to estimate in meta-analyses of typical sizes and have so far been rarely used outside sensitivity analysis.[20,21] From the description above it might seem that selection models are immune to spurious precision. Alas, not necessarily so: they are not robust to p-hacking. If individual results are not only selectively chosen for publication but actively p-hacked, they are not individually unbiased.[9] Reported (and thus potentially spurious) precision may add to the bias, because it is used as

a weight in maximum likelihood selection models. Both the Lombard effect and Taylor's law intuitions would then be inconsistent with selection models.

S1.2 Meta-Analysis Instrumental Variable Estimator

The methodological recommendation of this paper is to replace the reported standard error with the error's portion explainable by sample size. That is, we only use variation in errors that can be linked to variation in sample sizes. Because in most contexts the sample size is harder to increase than the standard error is to p-hack, the adjusted measure is likely to better capture the underlying precision. The variation in standard errors unrelated to variation in sample sizes is more susceptible to p-hacking. Conventional estimators can be rid of the unrelated variation, and the result is the meta-analysis instrumental variable estimator (MAIVE). As noted, we choose the adjusted version of PET-PEESE as the baseline MAIVE. PEESE uses squared standard errors, and so we instrument the reported *variance* with inverse sample size:

$$SE(\hat{\alpha})_i^2 = \psi_0 + \psi_1(1/N_i) + \nu_i, \tag{S1}$$

where $\hat{\alpha}$ is the effect size reported in a primary study, ψ_0 is a constant, N_i is the sample size of the primary study, and ν_i is an error term that soaks up, among other things, the spurious elements of the reported standard error related to p-hacking. The instrumented variance, to be used for adjusting current meta-analysis estimators, equals $\hat{\psi_0} + \hat{\psi_1}(1/N_i)$. In addition to PEESE, squared standard errors are also used in conventional inverse-variance weighting, so we find the quadratic specification natural. In estimators that require the standard error without squaring (such as PET, the first stage of PET-PEESE), we use $\sqrt{\hat{\psi_0} + \hat{\psi_1}(1/N_i)}$ for the adjustment of the standard error. Regressing in Equation S1 the reported standard error on the inverse of the square root of sample size would cause only minor quantitative changes; it is largely a matter of taste and aesthetics.

The idea of using an instrument for the standard error in meta-analysis goes back to Tom Stanley in 2005.[(22)] He notes that meta-regression techniques can suffer from attenuation bias, the "iron law of econometrics,"[(23,24)] because the reported standard errors on the right-hand side are estimated standard deviations. When a regressor is measured with random noise, the estimated slope coefficient is biased downwards. Stanley uses the instrumental variable approach to explore the robustness of funnel asymmetry tests, but does not employ it as an estimator of the underlying effect nor does he adjust the weights—in his influential 2005 *Journal of Economic Surveys* paper or any subsequent published work.

We believe that the attenuation problem is not important in meta-regression. Consider the basic Egger regression, a linear regression of reported effect sizes on the reported standard

errors—for simplicity without weights. To our knowledge, this regression was first used by Card and Krueger in their 1995 *American Economic Review* meta-analysis of the effect of minimum wage on employment,[25] a part of long-term research effort for which David Card won the 2021 Nobel Prize in economics. For attenuation bias to appear, the underlying relation needs to hold between estimates and standard deviations. Then the right-hand variable in meta-regression, the standard error, is a noisy version of the standard deviation. But the underlying relation really holds for estimates and reported standard errors. What matters is the resulting t-statistic, the ratio of estimates and standard errors. It follows that in the basic Card-Krueger-Egger regression there should be no attenuation bias. Measurement error bias is perhaps possible via inverse-variance weighting, or in models that combine meta-regression and selections models, such as endogenous kink[26]. We do not consider this mechanism.

Instead, we focus on reverse causality (selection on standard errors, a possibility noted in economics by Olken[27]) and omitted variables (resulting in p-hacking on both estimates and standard errors). These two issues were not discussed by Stanley, but have since been raised in footnotes by the co-authors of the present manuscript. Partial and imperfect versions of MAIVE, with limited and scattered justification, have appeared as robustness checks in our applied meta-analyses.[24,28,29] The estimator we prefer, MAIVE version of PET-PEESE without additional inverse-variance weights, has not been mentioned or used previously. Aside from Stanley's papers, we were inspired by the work of Egger et al.[30], Schmidt et al.[11], Hansen[31], and Nakagawa et al.[32,33], who explicitly or implicitly refer to the possibility of spurious precision in meta-analysis. Consider, for example, the following quote from Hansen, 2016, p. 1920:[31]

> The weighting of studies according to estimated precision is particularly problematic because the most unreliable estimates are also those with the least precise standard errors, and some of them will be treated as reliable simply due to error in their standard errors.

Related to our topic is also the work by Hedges[34–36], who develops adjustment techniques to reduce exaggerated precision (and compute appropriate effect size) in experiments where clustering is inadequately treated. This adjustment would provide a solution to spurious precision if inadequate clustering in primary studies was the only source of spurious precision. As we have noted, however, spurious precision can have many potential causes, and the one most plausible (p-hacking) is not addressed by clustering adjustment. Another stream of related literature is represented by Pustejovsky and Rodgers.[3,4] They point out that meta-analyses of standardized mean differences are generally biased because of a mechanical correlation between effect sizes and the corresponding standard errors. While this mechanism is not directly related to spurious precision as simulated in this paper, it creates the same endogeneity problem in

meta-regression and inverse-variance weighting. Once again, the solution put forward by Pustejovsky and Rodgers would solve the endogeneity problem if the mechanical correlation was the only source of endogeneity.

An obvious response to spurious precision could be to simply replace precision by a function of sample size, a replacement mentioned by several researchers [32,37–39]. While this would also remove spurious precision, the instrumental approach has 7 advantages. First, the underlying causal relationship is one between estimates and standard errors, not sample size. The t-statistic is important. Second, the optimal meta-analysis weight is based on inverse variance, not on sample size. [40] Third, the correlation between precision and sample size is not perfect. By using the instrumental variable approach, the confidence intervals can take into account that imperfect correlation in the first stage. Fourth, under classical assumptions, the funnel plot with estimate size and precision is symmetrical in the absence of publication bias. The assumptions are stronger for funnels that use sample size instead of precision. [41]

Fifth, the standard error is affected not only by sample size but also by estimation context. Variables related to clustering and identification in regression analysis can be used as instruments alongside sample size in MAIVE. The technique is thus more flexible, though it may be challenging to find instruments uncorrelated with the meta-regression error term. Sixth, when the correlation between precision and sample size is small, which may happen in observational research contexts (or in experimental research with poor randomization), the instrument can be weak. Statistical methods have been developed to deal with weak instruments and ensure valid inference, [42–45] an outcome not guaranteed when we simply replace variance with inverse sample size. Seventh, MAIVE automatically recomputes sample size to the units of precision reported by primary studies. This task can be achieved without the instrumental variable approach, but here the process is automatic. In consequence, with MAIVE all meta-analysis techniques can proceed as usual, now with a corrected measure of precision that is more likely than the reported, nominal precision to reflect the underlying, true precision. The message of our paper is not that inverse-variance weighting and funnel-based correction methods are wrong, but that they can be made considerably more robust with little cost.

In Table 2 of the main text we list the versions of the 7 estimators employed in simulations. For the simple average there is just one version, because the unweighted mean does not use the standard error at all. For the remaining 6 estimators we adjust the inverse-variance weight using the fitted value from the first-stage instrumental variable regression described above, creating the MAIVE variants of these estimators. For FE/WLS, WAAP, Andrews and Kasy, and p-uniform* we have just one version of adjustment: the standard error in these models is simply replaced with the square root of the fitted value. For regression-based models

(PET-PEESE and EK) we have in total 5 variants: i) no adjustment, ii) adjusted weights, iii) instrumented standard errors in the meta-regression but unadjusted weights, iv) adjusted weights and instrumented standard errors, and v) instrumented standard errors and omitted weights. Meta-regression techniques thus allow us to easily separate the effect of spurious precision on weighting and identification. The separation is more difficult for selection models; the adjusted versions that we use for selection models aggregate both effects and thus have weak statistical justification. This is an important issue we highlight for future research.

S1.3 Stylized Selection Scenario

S1.3.1 Simulation Setup

We simulate a meta-analysis environment where the object of interest is a regression coefficient. Striving for statistical significance, researchers engage in questionable research practices,[46] which give rise to selection on estimates or standard errors. We consider two alternative selection mechanisms, one stylized and one based on p-hacking. In the stylized selection environment, researchers unsatisfied with statistically insignificant results simply replace the obtained estimates or standard errors by values that are just enough to make the estimate statistically significant. (As noted, the lower standard error can be achieved via manipulation or a different treatment of clustering, heteroskedasticity, or nonstationarity.) Although admittedly simplistic, this selection mechanism allows us to conveniently control the relative degree of selection entering through estimates and standard errors.

Acknowledging the severe limitations of this selection scenario as a description of researchers' actual behavior, we use it merely to highlight the *qualitative* implications of selection on standard errors for the performance of the various estimators considered. That said, Table S1 and Table S2 show two examples that could potentially lead to an outcome observationally similar to our stylized scenario. Table S1 makes it clear that different computations of the standard error lead to dramatically different results and hence very different reported precision. While most researchers in this literature (the effect of class size on test scores) will agree that the computation of the standard error should take into account clustering, not all will agree on the precise form of clustering and whether bootstrap should be used. Table S2 shows that including or removing control variables can sometimes change the standard error without much change in the estimated effect. Indeed, in the last three columns the point estimate is virtually identical.

The other mechanism considered in our simulations and discussed in the next section is based on p-hacking, whereby the researcher engages in a continuous search of statistical significance by trying many different control variables, which changes estimates and standard errors at the same time. Although more realistic, this scenario features significantly less control over the

Table S1: Regression of test scores on class size: the importance of clustering

	Krueger (1999)	Replications using different computations of SE				
	(1)	(2) Bootstrap	(3) Class clusters	(4) School clusters	(5) Huber-White SE	(6) Plain vanilla SE
Small class	4.82	4.71	4.71	4.71	4.71	4.71
	(2.19)	(2.00)	(1.88)	(1.38)	(0.79)	(0.76)
Sample	5,861	5,743	5,743	5,743	5,743	5,743

Notes: The first column is adapted from Krueger, A.B. (1999): "Experimental Estimates of Education Production Functions." The Quarterly Journal of Economics 114(2), pp. 497–532.[47] Panel A (Kindergarten), OLS estimates (column 1) from Table V reproduced by permission of Oxford University Press on behalf of the President and Fellows of Harvard College. Excluded from the CC-BY License of this publication, for permissions contact journals.permissions@oup.com. In the remaining columns, we try to replicate Krueger's findings using the *webstar* dataset[48], which is derived from Project STAR, and apply different methods to compute the standard error. We are not able to precisely replicate Krueger's results because the original data and code used in his study are not available. Dependent variable: test score percentile. "Small class" denotes the treatment variable in the STAR experiment. All models also include one control variable (teacher aides) that is not reported in the table. Standard errors (SE) in parentheses.

Table S2: Regression of test scores on class size: the importance of controls

	(1)	(2)	(3)	(4)
Small class (treatment)	4.82	5.37	5.36	5.37
	(2.19)	(1.26)	(1.21)	(1.19)
White/Asian			8.35	8.44
			(1.35)	(1.36)
Girl			4.48	4.39
			(0.63)	(0.63)
Free lunch			-13.15	-13.07
			(0.77)	(0.77)
White teacher				-0.57
				(2.1)
Teacher experience				0.26
				(0.10)
Master's degree				-0.51
				(1.06)
School intercepts	No	Yes	Yes	Yes
Sample	5,861	5,861	5,861	5,861

Notes: Adapted from Krueger, A.B. (1999): "Experimental Estimates of Education Production Functions." The Quarterly Journal of Economics 114(2), pp. 497–532.[47] Panel A (Kindergarten), OLS estimates (columns 1–4) from Table V reproduced by permission of Oxford University Press on behalf of the President and Fellows of Harvard College. Excluded from the CC-BY License of this publication, for permissions contact journals.permissions@oup.com. Dependent variable: test score percentile. "Small class" denotes the treatment variable in the STAR experiment. Other variables denote characteristics of children and teachers. All models also include one control variable (teacher aides) that is not reported in the table. Standard errors in parentheses.

relative degree of selection on standard errors. One important advantage of p-hacking selection is its ability to generate selection of the two aforementioned flavors while, at the same time, causing heterogeneity in the reported estimates. But first back to the stylized scenario.

Generation of Primary Data. The primary data in the stylized scenario are generated according to

$$Y = \alpha_0 + \alpha_1 X + u, \tag{S2}$$

where $\alpha_0 = 0$ (without loss of generality), $X \sim U(0,1)$ and $u \sim N(0, \sigma_u^2)$. The parameter of interest to meta-analysis is α_1. Let i index each primary study and let there be M such primary studies, so that $i = 1, 2, \ldots, M$. Each primary study obtains random samples of size N_i for variables Y and X, estimates the regression model specified by Equation S2, and reports the OLS estimate of α_1 and its corresponding standard error.

Selection. Researchers prefer positive and statistically significant estimates of α_1. A fraction π of the researchers engage in questionable research practices and are willing to change either the reported estimates (E-selection) or on the standard errors (SE-selection) in order to inflate the statistical significance of their findings. They do so only when obtaining a positive but statistically insignificant estimate. Hence, when the obtained estimate is either negative or positive but statistically significant, the results are reported honestly. If, on the contrary, the obtained estimate is positive but statistically insignificant, the researcher changes the originally obtained findings with probability π.

With probability ϕ, a researcher willing to engage in questionable research practices chooses SE-selection, replacing the obtained standard error by a value that is just enough to achieve statistical significance at the 5% level; that is, the reported standard error is $\hat{\alpha}_1/1.96$. With probability 1-ϕ the researcher chooses instead E-selection, replacing the obtained estimate by a value that is just enough to achieve statistical significance at the 5% level; that is, the reported estimate is $SE(\hat{\alpha}_1) \times 1.96$.

In this environment, therefore, the overall magnitude of publication selection is measured by π and the relative importance of SE-selection versus E-selection is measured by ϕ. Note that, to keep perfect control on ϕ as measuring the relative importance of the two types of selection, we assume that researchers do not engage in both types simultaneously. This is also the reason why we assume that negative estimates are not subject to selection; otherwise, a negative estimate would have to become positive, which would necessarily involve E-selection. This restriction will be removed in the p-hacking scenario of the following section, so we ask the reader to tolerate it while we build toward a more realistic simulation.

Parameter Values and Distributions. We implement this type of selection by means of the parameter values and distributions summarized in Table S3. The number of studies in a meta-analysis is $M = 80$ in line with previous related simulations.[26,49] In Appendix S4 we consider simulations with $M = 30$. We assume that primary sample sizes are drawn from a uniform distribution over (30, 1000); in the next section we will calibrate the sample size distribution based on 436 published meta-analyses. We consider three alternative values of α_1: zero, one, and two. We interpret these values as representing no effect, a moderate effect, and a large effect, respectively. We assume that the probability of potentially engaging in questionable research practices is $\pi = 0.5$ and let ϕ vary from 0 to 1 in steps of 0.25. Note that $\phi = 0$ corresponds to pure E-selection, whereas $\phi = 1$ corresponds to pure SE-selection. Finally, we calibrate $\sigma_u^2 = 3.3$ in order to generate similar effective incidences of selection for $\alpha_1 = 0$ and $\alpha_1 = 2$, which is about 24% in both cases; for $\alpha_1 = 1$ it is a bit larger, at about 32%. (The effective incidence of selection is the overall fraction of findings subject to selection. Note that, in this scenario, effective selection incidence has a hump-shaped profile when graphed against α_1. This is because of the assumption of no selection on negative findings. Hence, when $\alpha_1 = 0$, selection incidence is not very high because approximately half of the estimates are negative. It gets higher for $\alpha_1 = 1$, because less estimates are then negative. And it gets lower again for $\alpha_1 = 2$ because more estimates become significantly positive naturally, even without selection.)

Table S3: Parameter values and distributions in the stylized selection scenario

Parameter/Variable	Description	Values/distribution
X	Regressor of the primary model	$\sim U(0,1)$
u	Error term of the primary model	$\sim N(0,\sigma_u^2)$
M	Number of studies/estimates	80
N_i	Sample size of the primary study	$\sim U(30,1000)$
α_1	Size of the true effect	0, 1, 2
π	Fraction of researchers susceptible to QRP	0.5
ϕ	Fraction of selection on standard errors	0, 0.25, 0.5, 0.75, 1
σ_u^2	Variance of the error term	3.3

Notes: QRP = questionable research practices. See text for explanation of the chosen values and distributions. When possible, in calibration we follow the tradition of previous simulations built for meta-analysis in the context of regression estimates.[7,26,49]

Replications and Statistics. To study the performance of the 7 baseline estimators and their MAIVE variants, we set the number of replications to $R = 2000$. We compute the bias and the mean squared error (MSE) of each estimator by averaging the estimation errors and the squared estimation errors over R, respectively. Hence, for a generic estimator z, the two

statistics are given by:

$$\text{Bias}(z) = \frac{1}{R}\sum_{i=1}^{R}(z_i - \alpha_1)$$

$$\text{MSE}(z) = \frac{1}{R}\sum_{i=1}^{R}(z_i - \alpha_1)^2$$

In addition, we also compute the coverage rates of each estimator by counting the number of confidence intervals that contain the true value of α_1 as a fraction of the total number of replications. Because of space and ease of exposition considerations, here we present only the results on bias and coverage rates, with MSE results reported in Appendix S6.

S1.3.2 Results

Figures S1-S4 show simulation results for the stylized selection scenario. Because the results for a large true effect are very similar to the results for a moderate effect, we relegate the former to Appendix S5. We do not show the results for Andrews and Kasy's estimator and p-uniform*, since they are not suited for this particular flavor of selection, giving huge biases and low coverage rates. We discussed the issue with the authors of p-uniform*, who confirmed that the estimator is not suitable to our stylized scenario. Thus it would be unfair to include these estimators now and compare them with the agnostic funnel-based techniques. All estimators are included in the more realistic p-hacking scenario of the following section.

The figures follow the same structure: panel (a) displays the unadjusted estimators, panels (b)-(e) show, one by one, the effect of adjusting FE/WLS, PET-PEESE, EK, and WAAP, and panel (f) compares the best versions of the adjusted estimators. Figures S1-S2 show the bias and coverage rates for the case of no effect ($\alpha_1 = 0$), then Figures S3-S4 display the same results for the case of a moderate effect ($\alpha_1 = 1$), and, finally, Figures S23-S25 in Appendix S5 do the same for the case of a large effect ($\alpha_1 = 2$). Results for MSE are shown in Appendix S6.

The most important results from Figure S1 are the following. When selection operates fully on estimates ($\phi = 0$), the simple average of the reported estimates shows a large bias, partly corrected by FE/WLS and WAAP, and entirely corrected by PET-PEESE and EK, as expected. When selection is fully on standard errors ($\phi = 1$), however, only the simple average is unbiased; FE/WLS, WAAP, PET-PEESE, and EK all show a similar positive bias. This implies that selection on standard errors is fundamentally a weighting problem. The bias arises from assigning too much weight to positively-selected estimates. Accounting for the correlation between estimates and standard errors (PET-PEESE and EK) does not solve the problem.

Because selection on standard errors is a weighting problem, adjusting the weights in

Fig. S1: Bias: stylized scenario, no effect ($\alpha_1 = 0$)

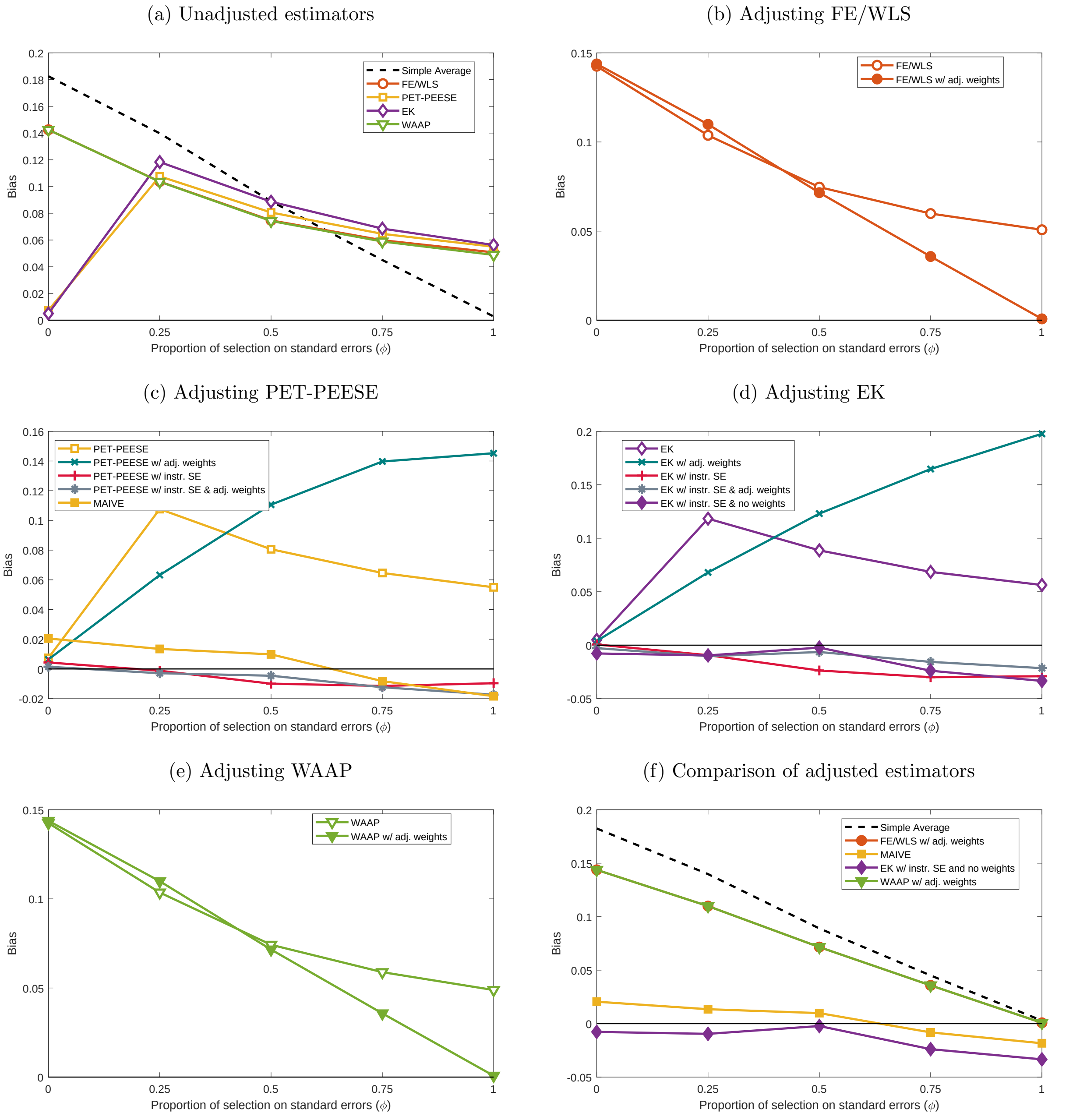

Notes: Figure shows the bias for the case of no effect in our stylized scenario. Panels show (a) a comparison of biases for all unadjusted estimators; bias for (b) the fixed effects or weighted least squares estimator with adjustment, (c) the adjusted precision-effect test and precision-effect estimate with standard errors, (d) the adjusted endogenous kink estimator, (e) the adjusted weighted average of adequately powered; and (f) a comparison of biases for all adjusted estimators.

Fig. S2: Coverage: stylized scenario, no effect ($\alpha_1 = 0$)

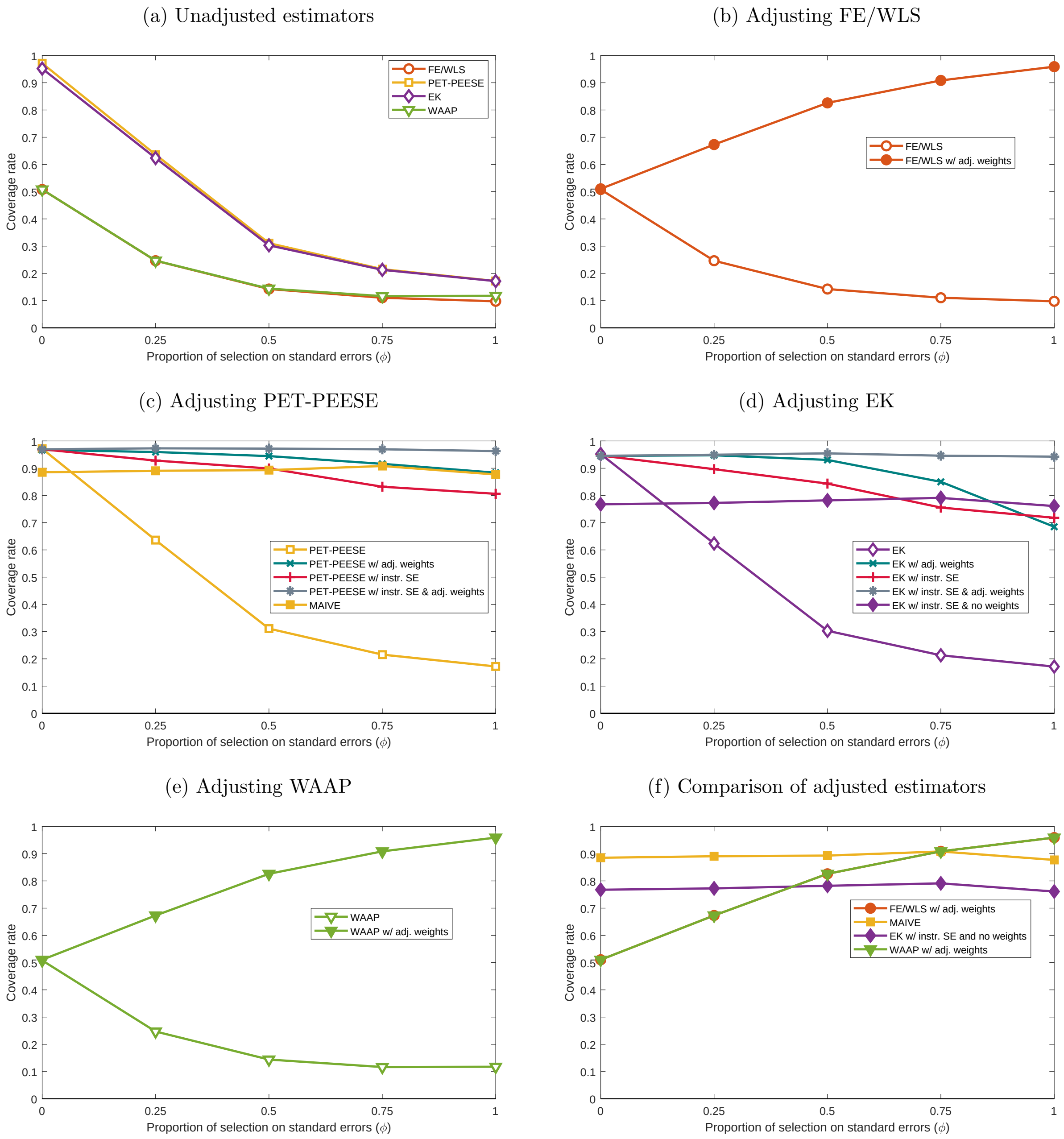

Notes: Figure shows the coverage rates for the case of no effect in our stylized scenario. Panels show (a) a comparison of coverage rates for all unadjusted estimators; coverage rate for (b) the fixed effects or weighted least squares estimator with adjustment, (c) the adjusted precision-effect test and precision-effect estimate with standard errors, (d) the adjusted endogenous kink estimator, (e) the adjusted weighted average of adequately powered; and (f) a comparison of coverage rates for all adjusted estimators.

Fig. S3: Bias: stylized scenario, moderate effect ($\alpha_1 = 1$)

(a) Unadjusted estimators

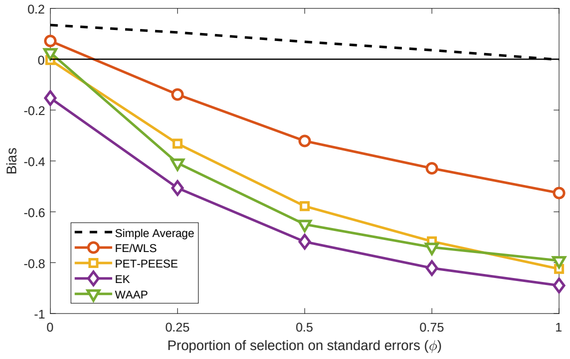

(b) Adjusting FE/WLS

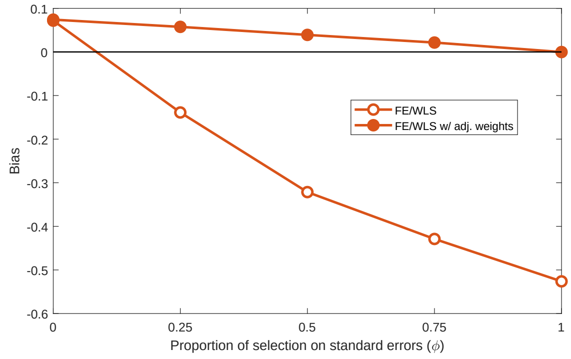

(c) Adjusting PET-PEESE

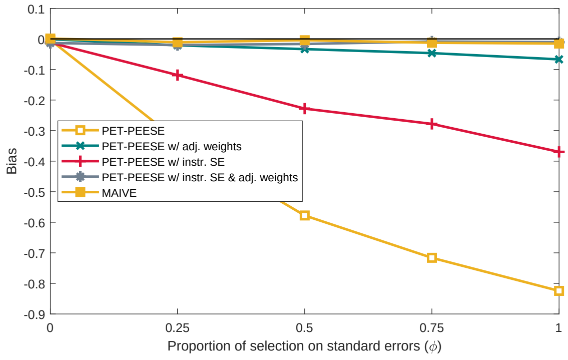

(d) Adjusting EK

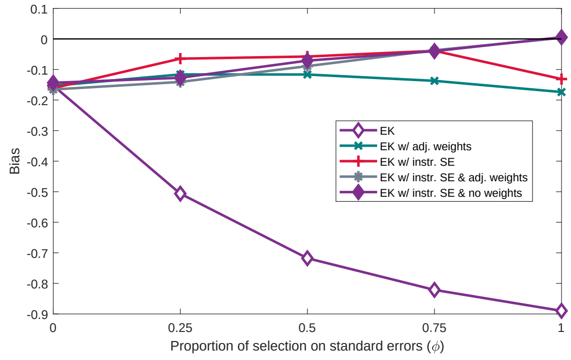

(e) Adjusting WAAP

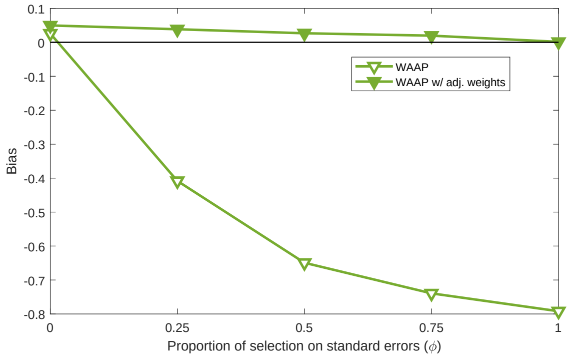

(f) Comparison of adjusted estimators

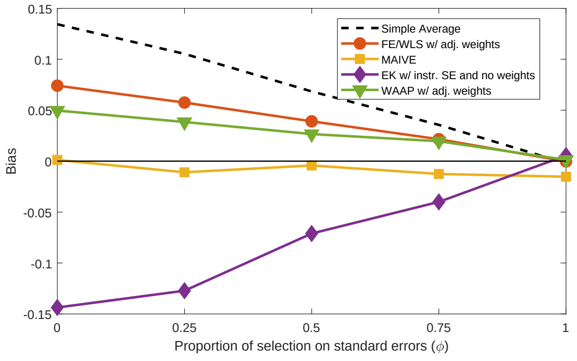

Notes: Figure shows the bias for the case of a moderate effect in our stylized scenario. Panels show (a) a comparison of biases for all unadjusted estimators; bias for (b) the fixed effects or weighted least squares estimator with adjustment, (c) the adjusted precision-effect test and precision-effect estimate with standard errors, (d) the adjusted endogenous kink estimator, (e) the adjusted weighted average of adequately powered; and (f) a comparison of biases for all adjusted estimators.

Fig. S4: Coverage: stylized scenario, moderate effect ($\alpha_1 = 1$)

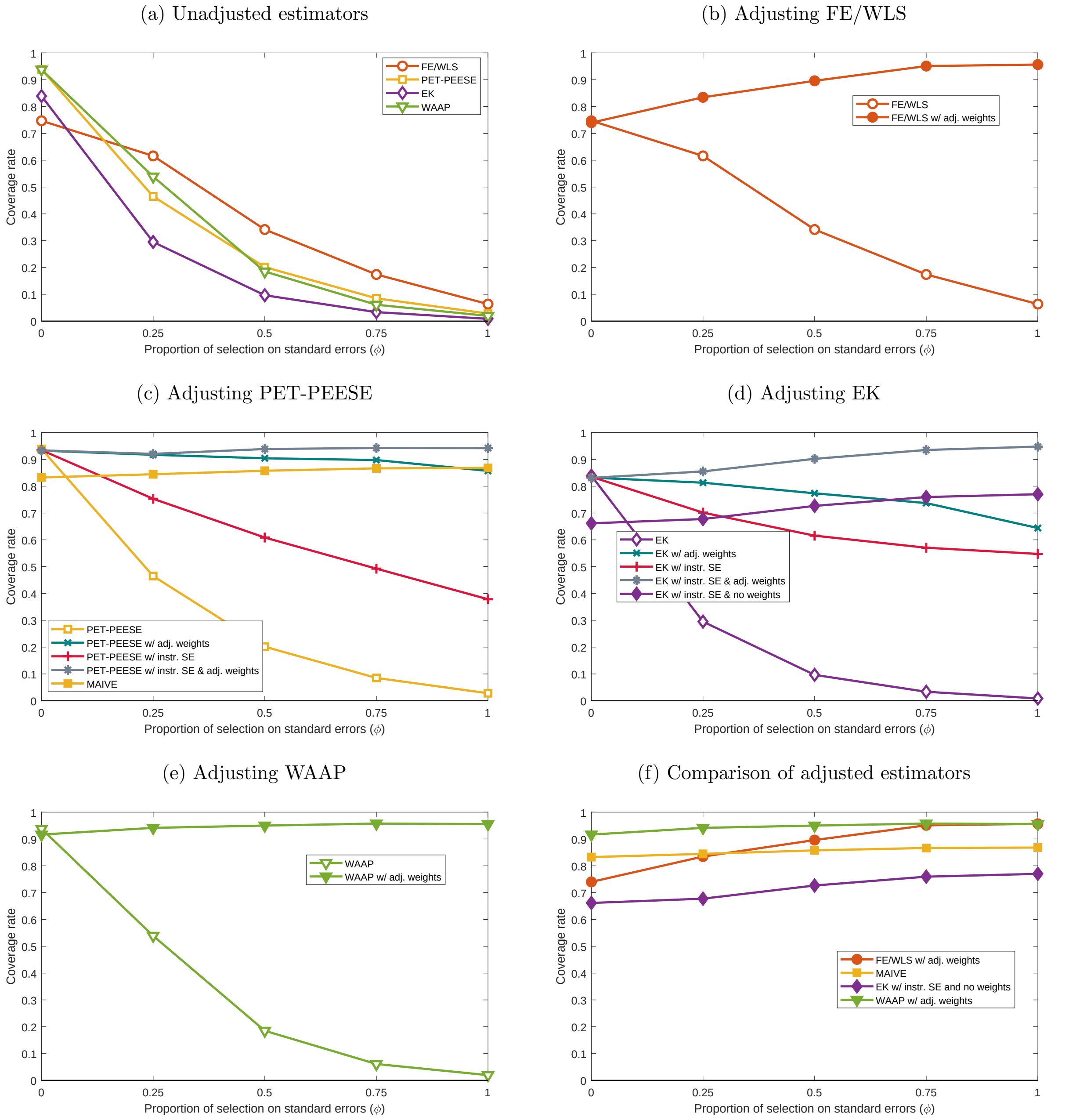

Notes: Figure shows the coverage rates for the case of a moderate effect in our stylized scenario. Panels show (a) a comparison of coverage rates for all unadjusted estimators; coverage rate for (b) the fixed effects or weighted least squares estimator with adjustment, (c) the adjusted precision-effect test and precision-effect estimate with standard errors, (d) the adjusted endogenous kink estimator, (e) the adjusted weighted average of adequately powered; and (f) a comparison of coverage rates for all adjusted estimators.

FE/WLS naturally makes it unbiased. The same applies for WAAP, which is virtually equal to FE/WLS in this case. But adjusting the weights by itself does not solve the problem if the standard errors are also included as a regressor (PET-PEESE and EK) and ϕ is large; in fact, it makes the bias larger. Not including the standard errors as a regressor, on the other hand, does not correct the bias if ϕ is small. Because ϕ is, in practice, unknown, the standard error should be included in the regression but instrumented (either in PET-PEESE or in EK). Instrumenting makes the bias much smaller.

What about instrumenting the SEs and at the same time adjusting the weights in PET-PEESE and EK? It turns out that this does not change much the bias relative to the case of only instrumenting the SEs. As we will see below, however, it does improve on other metrics (coverage, in particular) and for nonzero true effects. Dropping the weights when including and instrumenting the SEs in the regression also works reasonably well. The bottom line is that instrumenting SEs and adjusting or dropping the weights in PET-PEESE or EK reduces the selection bias for any value of ϕ.

Regarding the mean squared error, shown in Figure S26 in Appendix S6, adjusting the weights or instrumenting the SEs, despite decreasing the estimators' bias, often increases the estimators' variance and hence their MSE. But the lower variance of the unadjusted estimators is partly spurious and, coupled with large biases, results in low coverage. Hence, this increase in variance is a necessary adjustment to restore the nominal coverage of confidence intervals, as we will see below. For all following simulations, MAIVE adjustments also help improve MSE.

Results for coverage rates are shown in Figure S2. Like the bias, the coverage rates of the unadjusted estimators deteriorate rapidly as the degree of selection on standard errors (ϕ) increases. For as little as $\phi = 0.25$, the coverage rate drops from about 95% to about 60% for PET-PEESE and EK, and from 50% to 25% for FE/WLS and WAAP. Only adjusting the weights or only instrumenting the SEs improves the coverage rate substantially but this rate falls with ϕ. But instrumenting the SEs while adjusting the weights keeps the coverage rate at the nominal level in PET-PEESE and EK, irrespective of ϕ. Dropping instead of adjusting the weights leads to lower coverage. (Note that this is generally not the case in the more realistic p-hacking scenario of the next section, when dropping the weights often performs better than adjusting them.) Adjusting the weights in FE/WLS or WAAP improves coverage, but only sufficiently so for very large values of ϕ.

The most important results from Figures S3-S4, which concern a moderate underlying effect, are the following. For a positive true effect size ($\alpha_1 = 1$), the unadjusted estimators, and in particular those that explicitly address publication selection (PET-PEESE, EK, and WAAP), become extremely downward biased as the fraction of selection on standard errors (ϕ) increases.

(Here the bias due to inflated precision is much larger than classical publication bias due to inflated estimates.) The reason is that selection on standard errors, like selection on estimates, gives rise to a positive correlation between estimates and standard errors. The solution, as we saw above, should be readjusting the weights. But PET-PEESE and EK attribute this correlation to inflation in the estimates, and correct it down, causing a large negative bias for large ϕ. WAAP, likewise, is negatively biased, because it assigns too much weight to small estimates with artificially small standard errors.

As in the case of $\alpha_1 = 0$, adjusting the weights in FE/WLS and WAAP eliminates the bias for large ϕ. In PET-PEESE and EK, simultaneously instrumenting the SEs and adjusting (or dropping) the weights also eliminates the bias for large ϕ while keeping it low for small ϕ. However, only adjusting the weights or only instrumenting the SEs leads to larger negative biases for large ϕ. Overall, the fully adjusted PET-PEESE shows the smallest bias across values of ϕ. In contrast to $\alpha_1 = 0$, the MSEs of the unadjusted estimators now increase with ϕ (Figure S27). Moreover, the adjusted estimators now show smaller MSEs. The adjusted estimators, especially PET-PEESE and WAAP, attain a coverage rate very close to the nominal level.

The results for a large underlying effect are available in Appendix S5. The pattern of bias is very similar to the case of $\alpha_1 = 1$, with unadjusted estimators showing large negative biases for large ϕ. Adjusting the weights in FE/WLS and WAAP eliminates the bias for large ϕ. Fully adjusting PET-PEESE and EK also leads to low biases across the different values of ϕ. In terms of MSEs and coverage rates, the case of $\alpha_1 = 2$ is also similar to the case of $\alpha_1 = 1$: the adjusted estimators always attain lower MSEs and better coverage, very close to the nominal level in all cases for any value of ϕ.

In a nutshell, selection on standard errors causes conventional estimators to be biased. The bias can be huge and negative for positive true effects. Coverage rates deteriorate substantially in the presence of this type of selection. Adjusting or omitting the weights and instrumenting the standard errors in PET-PEESE and EK is effective in reducing the bias caused by selection on standard errors. The instrumental adjustment also restores the coverage rates of confidence intervals to their nominal levels. Adjusting the weights in FE/WLS and WAAP improves these models' performance, but they remain less effective than the MAIVE version of PET-PEESE in correcting the bias caused by selection on estimates.

S1.4 Selection Based on p-Hacking

S1.4.1 Simulation Setup

Generation of Primary Data. In the more realistic p-hacking simulation the data generating process for primary studies includes not one but two regressors, X_1 and X_2:

$$Y = \alpha_0 + \alpha_1 X_1 + \alpha_2 X_2 + u, \tag{S3}$$

where, again, $\alpha_0 = 0$ (without loss of generality), $X_1 \sim U(0, 1)$, and $u \sim N(0, \sigma_u^2)$. The second regressor, X_2, is a convex combination of X_1 and an independent random term $\epsilon \sim N(0, 1)$; i.e., $X_2 = \psi X_1 + (1 - \psi)\epsilon$, where $\psi \in (0, 1)$. Hence, X_1 and X_2 are positively correlated by construction, this correlation being governed by ψ. The parameter of interest to meta-analysis is α_1. In some simulations, this parameter is allowed to be random (Appendix S3). In this case, α_1 refers to its mean and $\sigma^2_{\alpha_1}$ is its variance. The M primary studies each report an OLS estimate and a corresponding standard error of α_1 using a sample of size N_i. The numerical values of the parameters depend on the selection mechanism assumed and are discussed below. A schematic overview of this simulation setup is available in Figure S5.

Selection. In this selection scenario, some researchers engage in questionable research practices by manipulating the specification of the model. In particular, we assume that primary studies start by estimating the correctly specified model (Equation S3). If the obtained estimate of α_1 is not positive and statistically significant in the correctly specified model, then, with probability π, the dissatisfied authors of such a primary study replace the true control variable X_2 by a different control variable, X_3. They try many such variables until they find one that 'works,' in the sense of turning the estimate of α_1 positive and statistically significant. (We implement this idea by first uniformly drawing a correlation coefficient between X_2 and X_3, constrained to be positive and less than 0.8. We then generate variable X_3 to match this correlation with X_2. The maximum correlation of 0.8 is imposed just to save on computing time, since very high correlations do not help the cause of getting statistical significance.)

Replacing X_2 by a related but weaker control variable X_3 helps achieving statistical significance through both E-selection and SE-selection. E-selection works through the bias it causes on the estimate of α_1. Because X_1 and X_2 are positively correlated, dropping X_2 in fact biases upwards the estimate of α_1 (omitted-variable bias), making statistical significance more likely. The bias increases with the correlation between X_1 and X_2 and with the value of α_2. The bias is somewhat mitigated by the inclusion of X_3. Note that, by inducing biases in the reported estimates, p-hacking causes not only publication selection but also excess variation in the reported

Fig. S5: Schematic overview of the p-hacking simulation setup

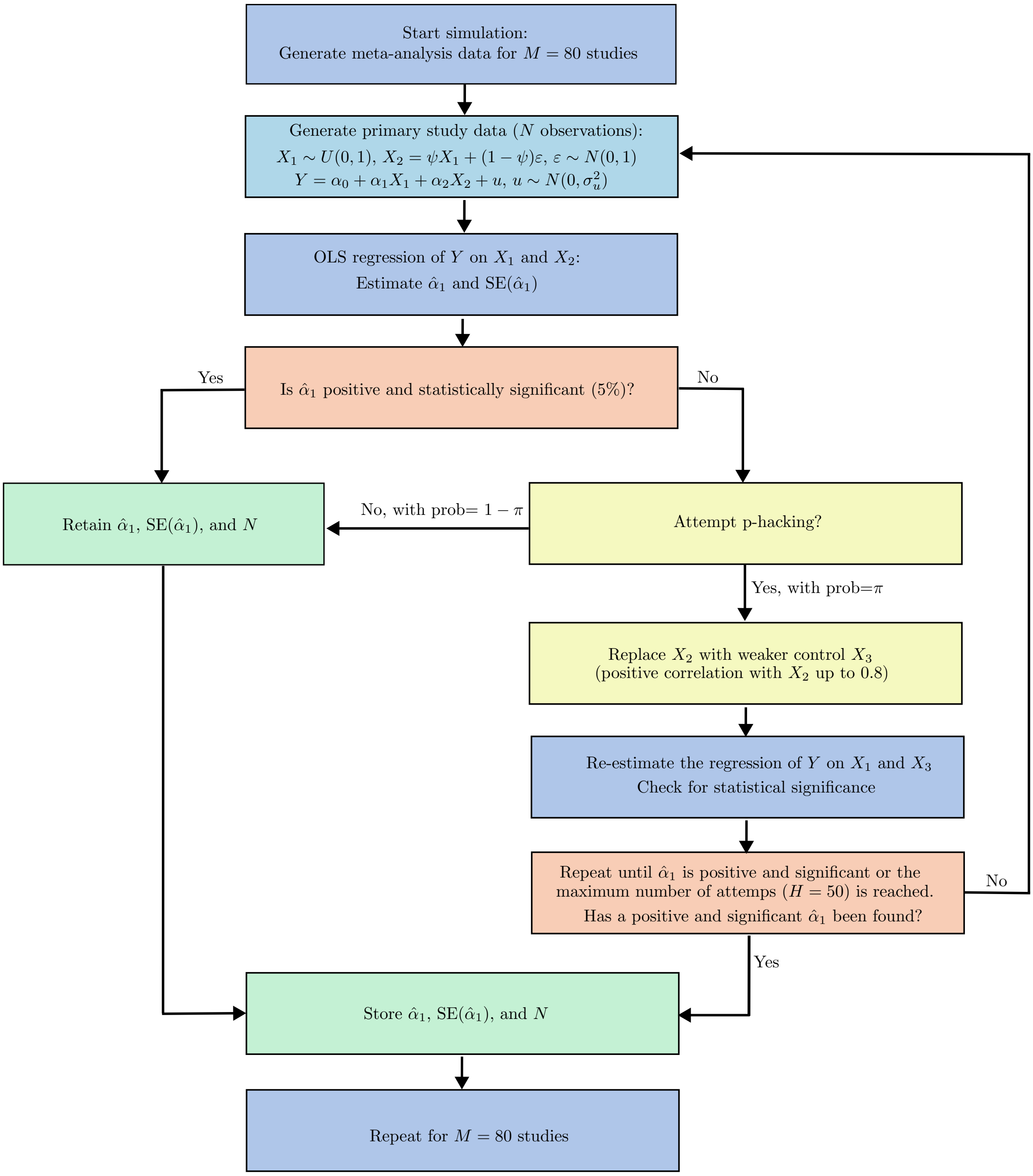

Notes: Figure provides a schematic overview of the simulation setup for the p-hacking scenario. For a detailed description of the simulation design and procedures, see Section S1.4.1.

findings (i.e., heterogeneity), a feature that characterizes most meta-analyses in observational research. In economics, for example, heterogeneity—rather then the unconditional mean—is often the focus of applied meta-analyses.

The p-hacking process also causes SE-selection: replacing X_2 by a weaker control decreases collinearity, thus artificially decreasing the SE of the estimate of α_1 relative to the SE in the correctly specified model. SE-selection increases with the correlation between X_1 and X_2, governed by ψ, but proportionally more so than E-selection. (To see this, note that E-selection depends on the bias of the estimate of α_1. At most, in case X_2 is dropped or replaced by an irrelevant control, this bias is given by $\psi\alpha_2$, and thus increases linearly with ψ. But SE-selection increases more than linearly with ψ. This is because the SE of $\hat{\alpha}_1$ in a model where X_2 is included can be written as $c\sqrt{1/(1-\psi^2)}$, where c depends on the variances of Y and X_1, on the R^2 of the regression, and the sample size, but is invariant to ψ. So the SE increases more than linearly with ψ, approaching infinity as ψ approaches one.) So this scenario still allows us to control the relative magnitude of SE-selection versus E-selection, albeit indirectly and imperfectly. It is not possible to fully decouple the two flavors of selection in this simulation environment, unlike in the stylized scenario. Figure S6 shows an example of p-hacking. Compared to Figure 1, the p-hacked estimates move not strictly east or north but northeast.

Fig. S6: Stylized example of a p-hacking environment

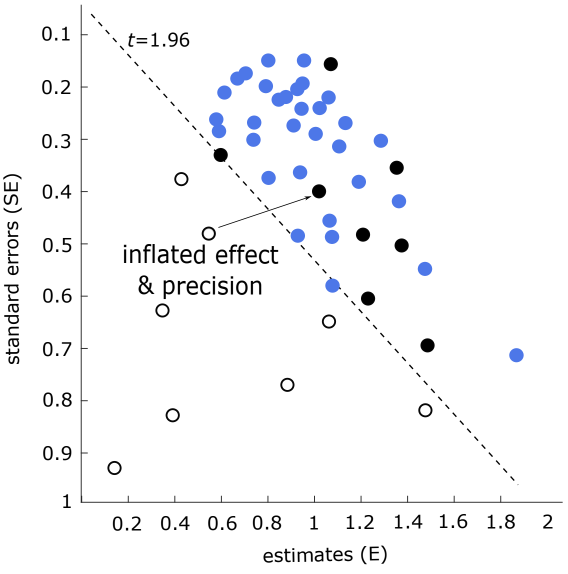

Notes: Blue-filled circles (lighter in grayscale) denote estimates statistically significant at the 5% level; these are reported. Hollow circles denote insignificant estimates, which are not reported in their original form but p-hacked to yield statistical significance (black-filled circles). Compared to Figure 1, the p-hacked estimates move not strictly east or north but northeast.

On a technical note, we limit the number of control variables attempted by p-hackers to H. If, at the H-th attempt, the estimate of α_1 remains negative or statistically insignificant, the

Table S4: Parameter values in the p-hacking selection scenario

Parameter/Variable	Description	Values/distribution
X_1	Main regressor of the primary model	$\sim U(0,1)$
X_2	Control variable of the primary model	$\psi X_1 + (1-\psi)\epsilon$
ϵ	Independent stochastic component of X_2	$\sim N(0,1)$
ψ	Correlation coefficient between X_1 and X_2	0.5, 0.6, 0.7, 0.8, 0.9
α_1	Size of the true effect	0, 1
α_2	Slope coefficient of X_2	2
u	Error term of the primary model	$\sim N(0,\sigma_u^2)$
σ_u^2	Variance of the error term	5.06
N_i	Sample size of the primary study	$\sim \Gamma(a,b)$
a	Shape parameter of the gamma distribution	0.65
b	Scale parameter of the gamma distribution	731
M	Number of studies/estimates	80
π	Fraction of potential p-hackers	0.5
H	p-hacking attempts before drawing new data	50

Notes: See text for explanation of the chosen values. When possible, in calibration we follow the tradition of previous simulations built for meta-analysis in the context of regression estimates.[(7,26,49,50)]. We also take inspiration from the simulation designs used by studies in other disciplines.[(3,4,51–53)]

p-hacker gives up the p-hacking search and resorts instead to an entirely new dataset, starting the process all over again. We do so because it may be extremely difficult (and time-consuming, from a computational perspective) in some cases to p-hack a very negative estimate into a significantly positive one. This is especially the case when α_1 is very small, which, by sampling error alone, may generate substantially negative estimates in some datasets.

Parameter Values and Distributions. We implement p-hacking selection using the parameter values and distributions summarized in Table S4. A key parameter in the simulations is ψ, the correlation between X_1 and X_2, since it governs the relative degree of SE-selection versus E-selection. The higher its values, the larger the relative degree of SE-selection. (We can quantify, for each value of ψ, the relative importance of SE-selection that would correspond to parameter ϕ in the stylized scenario; see below.) We let ψ take on values from 0.5 to 0.9 in steps of 0.1, using the middle value of 0.7 as the baseline value for the calibration of other parameters. In line with related simulation studies,[(26,49)] we assume a meta-analysis of $M = 80$ studies ($M = 30$ in Appendix S4) and a probability of engaging in publication selection (in this context, the fraction of potential p-hackers) of $\pi = 50\%$. The maximum number of p-hacking attempts before drawing new data is set at $H = 50$.

Regarding the true effect, we consider the cases where it is nil ($\alpha_1 = 0$) and where it is positive ($\alpha_1 = 1$). We do not separately consider a case of a "large effect" as in the stylized scenario, because once again the results would be qualitatively identical to $\alpha_1 = 1$, so we have just one value for the positive effect. We set the remaining parameters (especially σ_u^2) so that

$\alpha_1 = 1$ is neither too small nor too large an effect. If σ_u^2 is too large, $\alpha_1 = 1$ effectively represents a small effect. Conversely, when σ_u^2 is too small, $\alpha_1 = 1$ effectively represents a large effect. Moreover, the larger the effective size of α_1, the smaller the effective incidence of publication selection, eventually dropping to zero. For $\alpha_1 = 0$, the effective incidence of selection is about 49%. (This assumes that primary studies test the null hypothesis that $\alpha_1 = 0$ using a two-sided test at the 5% level. Hence, the probability of not finding a significantly positive value is 97.5%. Because only half of the studies engage in publication selection, the effective selection incidence is half of this rate, that is, 48.75%.) We then choose σ_u^2 so that the effective selection incidence for $\alpha_1 = 1$ is half of the incidence for $\alpha_1 = 0$—that is, 24%. The implied value of σ_u^2 is 5.06.

Note that for generality we focus on non-standardized effect sizes, regression coefficients. If we focus on a standardized effect size, such as standardized mean difference, we inevitably introduce a mechanical correlation between estimates and standard errors, which would obscure our simulations because our goal in this paper is to model spurious precision that arises due to selection on standard errors, not other sources of endogeneity of the standard error (such as those investigated, for example, by Pustejovsky and Rodgers[3]). The interpretation of the effect size is not important for our simulation as long as the values fall within a range that matters for publication selection. For simplicity, we chose true effect values of 0 a 1 and calibrate the variance of the error term for publication bias to matter for these values. However, in standardized terms, given that the standard deviation of Y is about 2.35 and that of X_1 is about 0.29, $\alpha_1 = 1$ implies that one standard deviation increase in X_1 increases Y by about 0.29/2.35=0.12 standard deviations. That is, the partial correlation coefficient is about 0.12. While the value may seem low, it is consistent with common meta-analyses in our second empirical application: across hundreds of meta-analyses and 170,900 partial correlations, the mean is 0.16 and the median 0.08. For $\alpha_1 = 2$, the standard deviation of Y increases slightly to about 2.52, so that the standardized effect is 2*0.29/2.52=0.23—i.e., one standard deviation of X_1 increases Y by about 0.23 standard deviations—almost twice as large as for $\alpha_1 = 1$.

The p-hacking scenario generates heterogeneity. Given σ_u^2, α_1, and ψ, the main parameter determining the degree of parameter heterogeneity is α_2. Based on the typical findings of applied meta-analyses, simulation studies[26,49] often assume values of I^2 of at least 70%. By setting $\alpha_2 = 2$, we arrive at an I^2 of about 73% for $\alpha_1 = 0$ (for $\alpha_1 = 1$, the I^2 is about half). When allowing for true effect heterogeneity (Appendix S3), we set $\sigma_{\alpha_1}^2$ at 0.64. This further increases the I^2 by about 9 percentage points when $\alpha_1 = 0$; and by about 40 percentage points when $\alpha_1 = 1$. The sample size of a primary study, N_i, is drawn from a truncated gamma distribution $\Gamma(a, b)$. Note that the mean of this distribution is given by ab and the variance by ab^2. We choose the values of a and b to match the research record. Using a database of 436

meta-analyses in economics provided to us by Chris Doucouliagos,[(54)] we find the medians of the mean and variance of the sample sizes within the individual meta-analyses to be 473 and 588^2, respectively; using these as target values, we find the required gamma parameters to be $a = 0.65$ and $b = 731$. We truncate the distribution from below, so that a sample size is never smaller than 30.

Implied Relative Degree of SE-Selection. As mentioned above, we control the relative degrees of selection on estimates and selection on standard errors indirectly through the parameter ψ. In the stylized scenario, this relative degree was controlled directly through ϕ. Although we cannot control ϕ directly here, we can nevertheless infer its size for each value of ψ. To do so, start by denoting, for the set of selected estimates, the observed (post-selection, hacked) t-statistic of $\hat{\alpha}_1$ by $t = \hat{\alpha}_1/\text{SE}(\hat{\alpha}_1)$ and the original (pre-selection, unhacked) t-statistic by $t^* = \hat{\alpha}_1^*/\text{SE}(\hat{\alpha}_1)^*$. Of course, the objective of selection is to increase the size of the t-statistic, so $t > t^*$. E-selection implies $\hat{\alpha}_1 > \hat{\alpha}_1^*$ and SE-selection implies $\text{SE}(\hat{\alpha}_1) < \text{SE}(\hat{\alpha}_1)^*$. In the p-hacking scenario, however, both types usually occur simultaneously and ϕ measures the relative importance of each. Because $t/t^* = (\hat{\alpha}_1/\hat{\alpha}_1^*) \times (\text{SE}(\hat{\alpha}_1)^*/\text{SE}(\hat{\alpha}_1))$, it follows that

$$\ln\left(\frac{t}{t^*}\right) = \ln\left(\frac{\hat{\alpha}_1}{\hat{\alpha}_1^*}\right) + \ln\left(\frac{\text{SE}(\hat{\alpha}_1)^*}{\text{SE}(\hat{\alpha}_1)}\right),$$

which decomposes the amount of publication selection in selected estimates (percent change of the t-statistic) into its E-selection component (given by the first term, the percent increase of $\hat{\alpha}_1$ after selection) and its SE-selection component (given by the second term, the percent decrease in $\text{SE}(\hat{\alpha}_1)$ after selection). Hence, the relative importance of SE-selection can be approximated by the relative size of the second term:

$$\phi = \frac{\ln(\text{SE}(\hat{\alpha}_1)^*/\text{SE}(\hat{\alpha}_1))}{\ln(t/t^*)}. \tag{S4}$$

On a technical note, we need to impose some restrictions to ensure that $0 \leq \phi \leq 1$. If, for a particular selected estimate, $\text{SE}(\hat{\alpha}_1) > \text{SE}(\hat{\alpha}_1)^*$, then selection must have occurred entirely through the estimates and we set $\phi = 0$. If, on the other hand, $\hat{\alpha}_1 < \hat{\alpha}_1^*$, then selection must have occurred through the standard errors, and we set $\phi = 1$. Table S5 shows the values of ϕ corresponding to the various values of ψ. Clearly, the relative importance of SE-selection increases with ψ.

S1.4.2 Results

In the p-hacking scenario we simulate all estimators in all versions described in Table 2 of the main text. The results are reported following the same structure as in the stylized simulation

Table S5: The fraction of SE-selection corresponding to correlation between regressors

True effect (α_1)	Correlation (ψ)				
	0.5	0.6	0.7	0.8	0.9
0	0.009	0.015	0.041	0.095	0.216
1	0.026	0.040	0.102	0.207	0.370

Notes: The table shows the fraction of selection on standard errors relative to selection on estimates (ϕ in the stylized scenario) mapped to the correlation coefficient between X_1 and X_2 (ψ in the p-hacking scenario) and the true effect. For example, if the correlation is 0.9 and the true effect is 1, the implied fraction of SE-selection is 0.37.

scenario. Figures S7-S8 show the bias and coverage for no effect ($\alpha_1 = 0$) and various values of ψ. S9 shows the results for a positive effect ($\alpha_1 = 1$). Results showing MSE are available in Appendix S6. Note that the horizontal axis now does not measure the relative degree of SE-selection, but can be recomputed to that degree using Table S5.

We then consider two extensions of the baseline p-hacking simulation framework. First, we consider true effect heterogeneity by allowing the true effect to be drawn from a normal distribution with mean α_1 and variance $\sigma^2_{\alpha_1} = 0.64$. Figures S11–S16 in Appendix S3 show the results for bias, MSE, and coverage for a (mean) true effect of 0 and a (mean) true effect of 1. Second, we consider smaller meta-samples of size $M = 30$. The results are reported in Figures S17–S22 in Appendix S4.

In addition, Table S6 reports the average F-statistics of the first-stage regression. The F-statistics tend to decline with ψ, since an increased ψ indicates more SE-selection. In any case, the average F is at least in the hundreds, if not in the thousands. This is consistent with the results from the our empirical applications (Appendix S2), where the F-statistics of most meta-analyses for which a first-stage regression can be run are also at least in the hundreds. We conclude that inverse sample size is typically a strong instrument for reported variance.

Regarding Figure S7, the simple average measures the effect size inflation caused by E-selection. It increases with ψ, because E-selection increases with ψ: the larger its value, the larger the (positive) biases in the individual studies subject to selection. All results should thus be evaluated relative to the simple average. SE-selection also increases with ψ, and more than proportionally so (see Table S5). For $\psi = 0.5$, SE-selection is fairly low and all methods correct a large chunk of the selection bias. As ψ goes up, so does the relative importance of SE-selection. As a consequence, the capacity of these methods to correct for the increasing bias deteriorates rapidly. For most methods, the biases due to spurious precision eventually surpass

Fig. S7: Bias: p-hacking selection, no effect ($\alpha_1 = 0$), various values of ψ

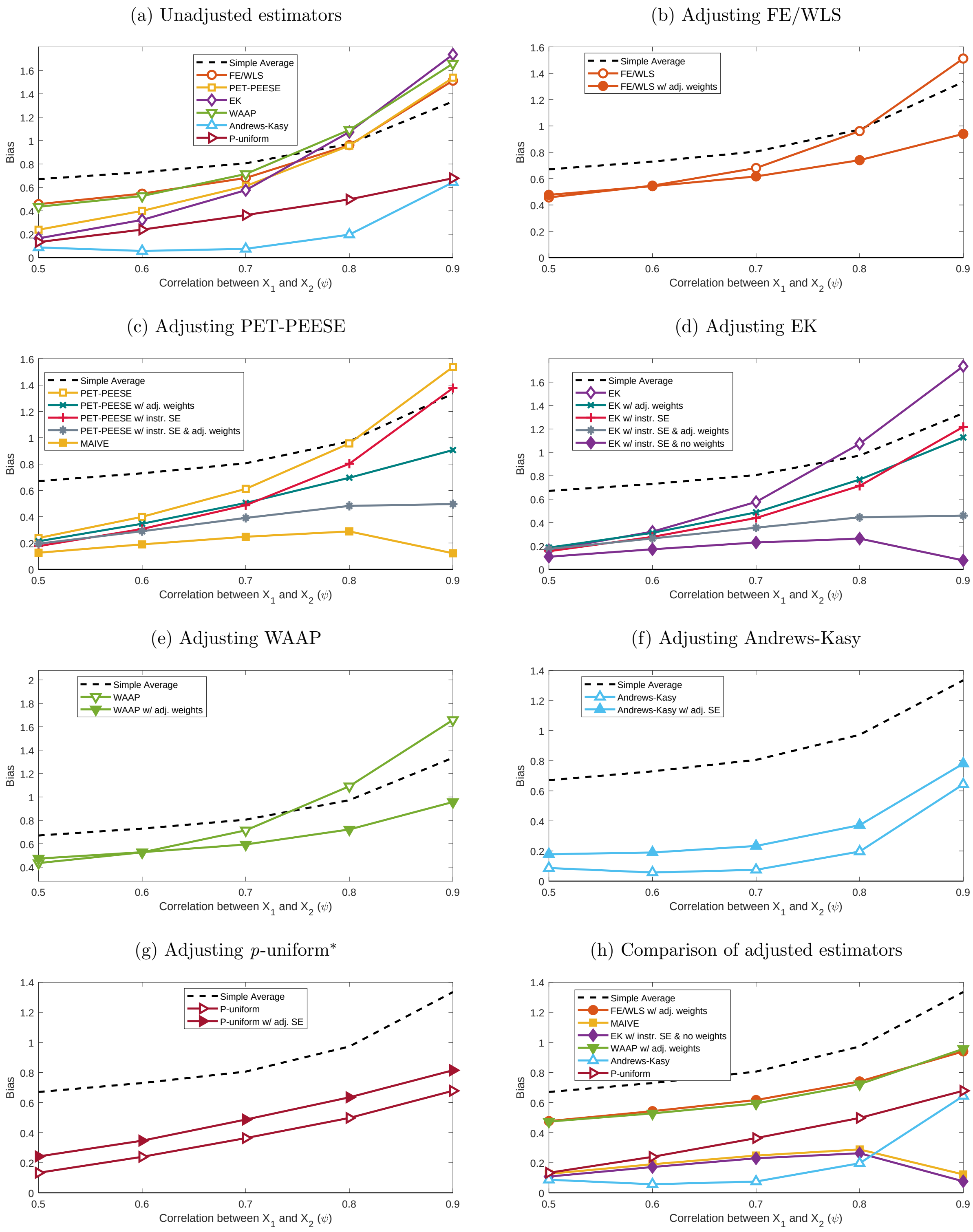

Notes: Figure shows the bias for the case of no effect in the p-hacking scenario. Panels show (a) a comparison of biases for all unadjusted estimators; bias for (b) the fixed effects or weighted least squares estimator with adjustment, (c) the adjusted precision-effect test and precision-effect estimate with standard errors, (d) the adjusted endogenous kink estimator, (e) the adjusted weighted average of adequately powered; (f) the adjusted Andrews and Kasy estimator, (g) the adjusted p-uniform* method; and (h) a comparison of biases for all adjusted estimators.

Fig. S8: Coverage: p-hacking selection, no effect ($\alpha_1 = 0$), various values of ψ

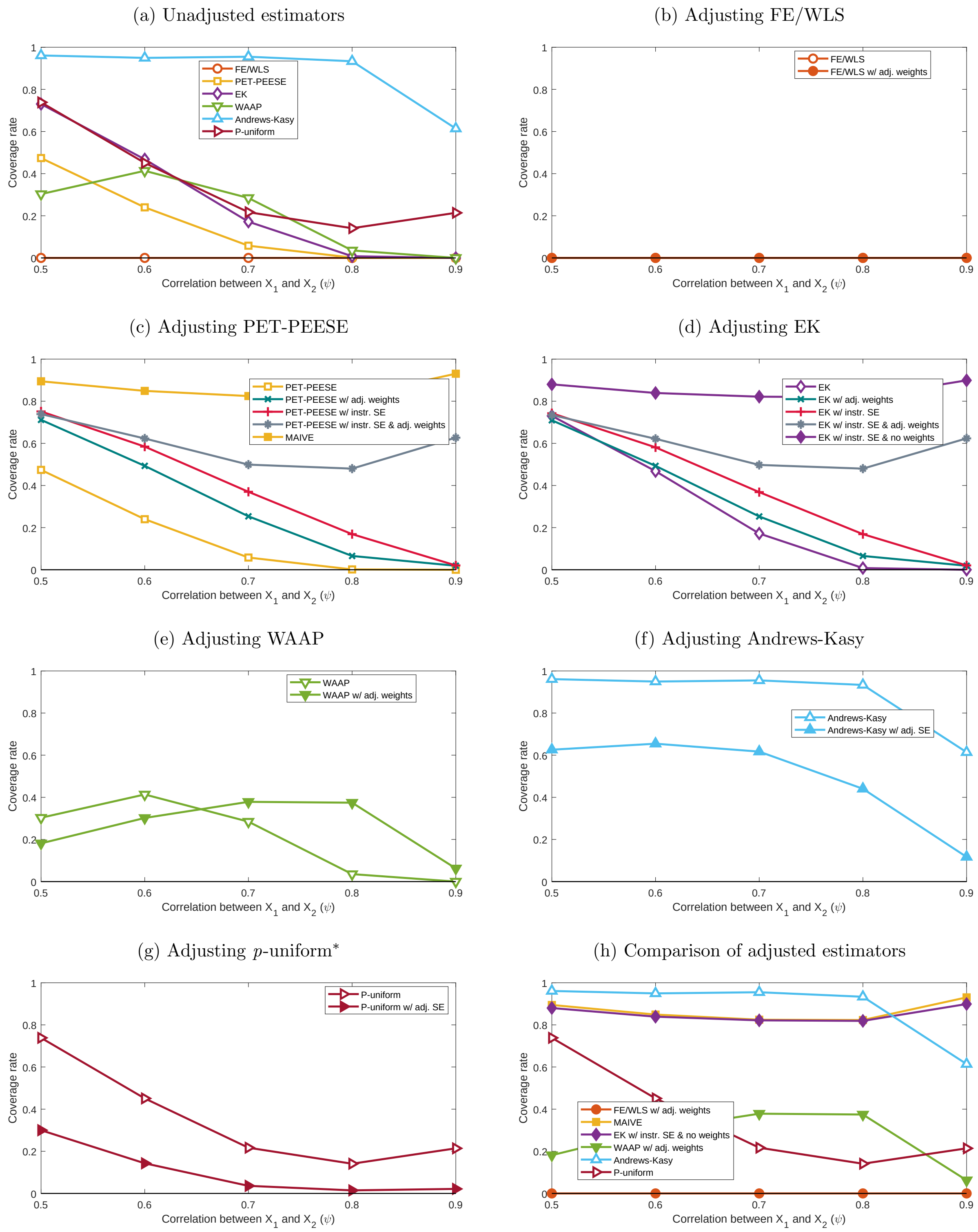

Notes: Figure shows the coverage rates for the case of no effect in the p-hacking scenario. Panels show (a) a comparison of coverage rates for all unadjusted estimators; coverage rate for (b) the fixed effects or weighted least squares estimator with adjustment, (c) the adjusted precision-effect test and precision-effect estimate with standard errors, (d) the adjusted endogenous kink estimator, (e) the adjusted weighted average of adequately powered; (f) the adjusted Andrews and Kasy estimator, (g) the adjusted p-uniform* method; and (h) a comparison of coverage rates for all adjusted estimators.

Fig. S9: Coverage: p-hacking selection, positive effect ($\alpha_1 = 1$), various values of ψ

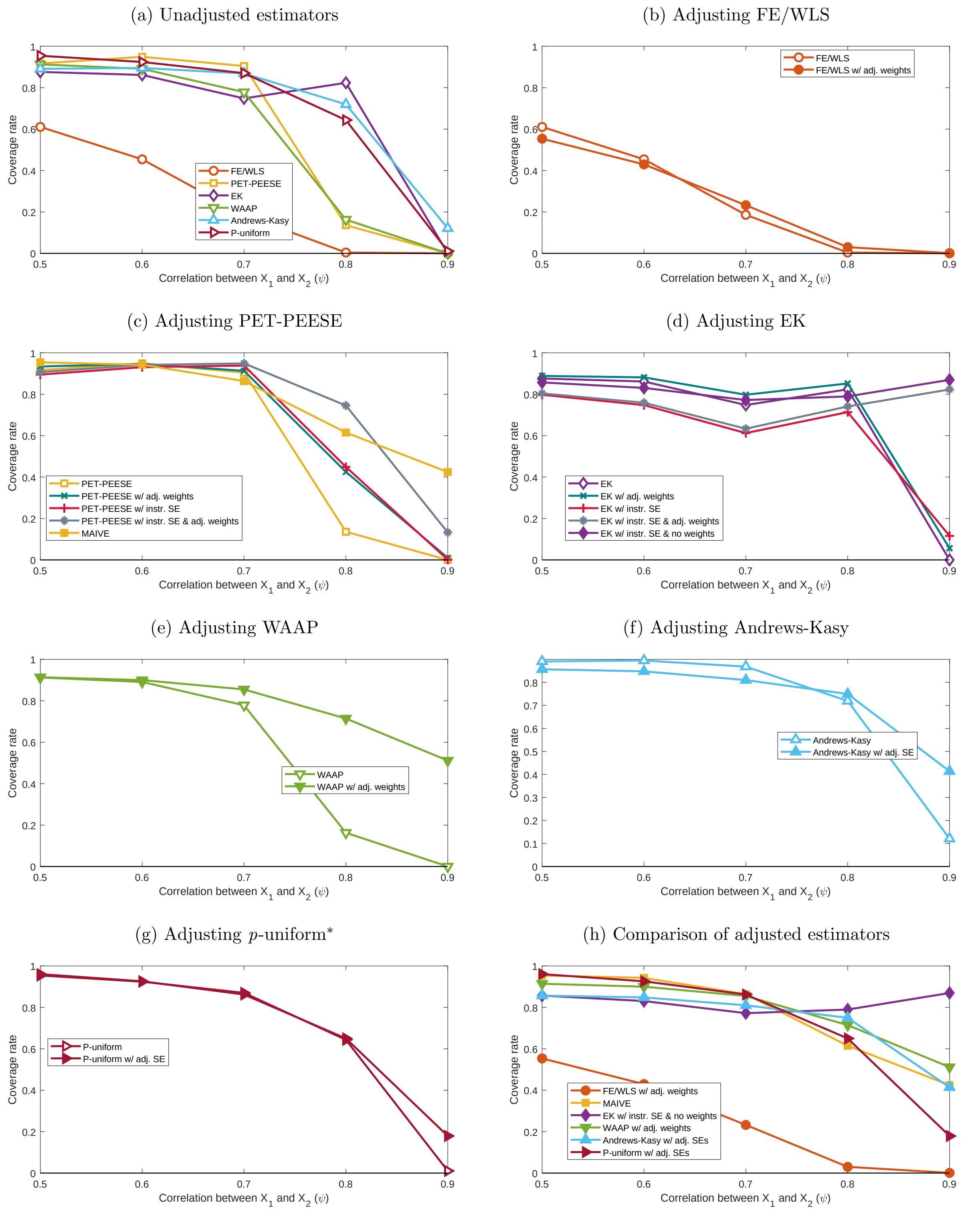

Notes: Figure shows the bias for the case of positive effect in the p-hacking scenario. Panels show (a) a comparison of biases for all unadjusted estimators; bias for (b) the fixed effects or weighted least squares estimator with adjustment, (c) the adjusted precision-effect test and precision-effect estimate with standard errors, (d) the adjusted endogenous kink estimator, (e) the adjusted weighted average of adequately powered; (f) the adjusted Andrews and Kasy estimator, (g) the adjusted p-uniform* method; and (h) a comparison of biases for all adjusted estimators.

Table S6: First-stage average F-statistics in the p-hacking environment

	ψ				
	0.5	0.6	0.7	0.8	0.9
(a) Baseline:					
$\alpha_1 = 0$	1310	1335	1163	774	416
$\alpha_1 = 1$	1371	1345	1090	589	247
(b) Heterogeneity ($\sigma^2_{\alpha_1} = 0.64$)					
$\alpha_1 = 0$	1364	1323	1191	776	409
$\alpha_1 = 1$	1399	1384	1126	641	270
(c) Smaller samples ($M = 30$)					
$\alpha_1 = 0$	841	843	719	431	214
$\alpha_1 = 1$	886	909	678	311	130

Notes: The table reports the average F-statistic across the $R = 2000$ replications in each simulation environment.

the selection bias itself; i.e., the methods actually worsen the existing publication bias. Notable exceptions are Andrews-Kasy and p-uniform*, which always correct part of the bias.

Our proposed adjustment improves all methods except Andrews-Kasy and p-uniform*—where, as we have noted, the adjustment has weaker statistical justification. In EK and PET-PEESE, it works better when instrumenting the SEs in the meta-regression and using no weights. Thus corrected, these estimators tend to perform the best, especially for large ψ. The profiles of MSE in Figure S28 in Appendix S6 are very similar to the profiles of bias in Figure S7.

Regarding Figure S8, the coverage rates also decrease markedly with ψ. Only Andrews-Kasy manages to sustain acceptable coverage for ψ up to 0.75. At this value of ψ, all the other estimators show very low coverage rates of 20% or even lower. Our adjustment works relatively well in PET-PEESE and EK, especially when no weights are used, with coverage rates above 80% (still below the 95% nominal level, though). It is important to emphasize the role of the weights as ψ increases. Only instrumenting the SEs in the meta-regression does not work. Better, though still insufficient, is to also adjust the weights; much better is to drop them altogether. For Andrews-Kasy and p-uniform*, our adjustment does not work at all.

Regarding S9, the results for $\alpha_1 = 1$ are qualitatively similar to those seen above for $\alpha_1 = 0$. Quantitatively, the biases and MSEs are now typically smaller and coverage is better; this is because a larger true effect reduces the overall size of publication bias (less p-hacking is needed). All methods still show a decreasing capacity to correct for publication bias as ψ increases. Eventually, the biases of the correction methods become larger than publication bias itself (measured by the simple average), although this happens at a higher ψ than before. Our adjustment now improves the performance of all methods, including selection models. In PET-PEESE, adjusting the weights is comparable to dropping them, and a similar observation

applies to EK. WAAP with adjusted weights and SEs now does almost as well as PET-PEESE; adjusted EK without weights seems to work even better.

Regarding Figures S11–S16 in Appendix S3, allowing for true effect heterogeneity ($\sigma^2_{\alpha_1} = 0.64$) does not change much the results, at least quantitatively. MAIVE still performs relatively well. Regarding Figures S17–S22 in Appendix S4, for $M = 30$ the main difference is that the Andrews-Kasy selection model becomes less reliable, with huge biases and MSEs.

The reader may be surprised that the bias of unadjusted methods is now positive, while in the stylized scenario the bias was negative (both for $\alpha_1 = 1$). The reason is that in the p-hacking scenario, selection on estimates and selection on standard errors (or, analogously, the Lombard effect and Taylor's rule) interact. In the stylized scenario the bias is always downwards if $\alpha_1 > 0$, because small estimates are assigned small standard errors to be significant, which gives small estimates too much weight. In the p-hacking scenario estimates can be too large and too precise at the same time, which creates the upward bias. Because we consider the p-hacking scenario substantially more realistic than the stylized scenario, we expect that meta-analysis models are biased upwards due to spurious precision. This conclusion is consistent with Kvarven et al. (2020) [16], who argue that common meta-analysis models are biased upwards compared to preregistered multilab replications.

In a nutshell, the more realistic p-hacking simulation implies that the MAIVE version of PET-PEESE without additional inverse-variance weights is more robust to spurious precision than available alternatives. When spurious precision is important, the method clearly dominates unadjusted estimators, including selection models. When the degree of spurious precision is negligible, the performance of MAIVE is similar to unadjusted methods, though it can be sometimes (especially for a zero true effect) beaten by selection models. Based on the simulation results and Table S5, the simple unweighted mean can often beat sophisticated unadjusted estimators for a ratio of SE-selection as low as 0.2 relative to total amount of selection.

S1.5 Additional Discussion

The meta-analysis instrumental variable estimator (MAIVE) reduces the problem of spurious precision by using inverse sample size as an instrument for reported variance. That is, we regress the reported squared standard errors on the inverse of the number of observations used in the primary study. The fitted values from this regression are then used instead of reported variance in the PEESE meta-regression. Standard weighted means, funnel plots, and funnel-based methods can be adjusted similarly to make them reasonably robust to spurious precision. The entire meta-analysis toolkit can be salvaged with this modification.

The instrumental approach has seven benefits over using sample size as a proxy for precision,

and we explain them in Subsection S1.2. There are at least two costs as well, both compared to the proxy approach and the classical one that relies on reported precision. First, MAIVE is more complex since it involves an additional regression and computation of fitted values and valid confidence intervals. But the instrumental approach is available in most statistical programs. We create the `maive` package for R, which makes estimation easy for meta-analysts unfamiliar with instrumental variables. Second, the additional regression makes MAIVE noisier compared to conventional techniques. When a meta-analyst is sure there can be no spurious precision in her data, using reported precision without instruments will yield unbiased and more efficient meta-analysis estimates. The lack of spurious precision can be tested approximately by employing the Hausman specification test:[(55)] if the coefficients estimated in MAIVE are far from those of an unadjusted PEESE, spurious precision is likely an issue.

A discussion is in order regarding the practical application of MAIVE—pronounced, by the way, as the Irish name Maeve. The default instrument is the overall sample size, not degrees of freedom, because the latter depends on clustering units. It is true that, with clustering, the standard error is approximately proportional to the number of clusters rather than to the raw sample size. However, the proper unit of clustering is not always clear[(56–58)]. We provide several examples in the paper—see especially Table S1 and the related discussion. For instance, with panel data, one can cluster at the level of cross-sectional units, years, or both simultaneously. In other words, the proper unit of clustering is often unobserved.

Additionally, clustered standard errors tend to be biased downward when the number of clusters is small[(59,60)], and in such cases, bootstrapping often provides a better approximation of the correct standard error. Another reason to prefer raw sample size over degrees of freedom is that, when used in MAIVE, the latter fails to correct for the underestimation of standard errors in studies that ignore clustering, as these studies receive greater adjusted precision due to their nominally larger degrees of freedom. Nevertheless, if the appropriate clustering unit is well established in the literature, the proper number of clusters in the primary study should be used instead of sample size or as an additional instrument along with sample size, regardless of whether the primary studies correctly account for clustering in their analysis. If it is challenging to determine the correct cluster choice, using the smallest possible number of clusters as an instrument may serve as a conservative approach.

Related to this discussion is the work of Hedges[(34–36)], who develops adjustment techniques to reduce exaggerated precision and compute appropriate effect sizes in experiments where clustering is inadequately addressed. These adjustments should be used when possible and would resolve spurious precision if inadequate clustering were the sole source of the problem.

We prefer the MAIVE version of PEESE without weights (after testing with unweighted

MAIVE-PET whether the true effect is nonzero). This parsimonious specification intuitively fits both panels of Figure 1 of the main text. The `maive` package allows for optional adjusted weights. Researchers may choose a MAIVE version of another estimator, such as endogenous kink. The package also runs the Hausman test. Because PEESE is heteroskedastic by definition and we prefer not to use inverse-variance weights, the package produces heteroskedasticity-robust standard errors by default.

When studies report multiple estimates, standard errors in MAIVE—and any meta-analysis estimator—should be clustered at the study level, again a default option. The issue of clustering and, in general, robust variance estimation for meta-analysis is handled in detail by Pustejovsky and Tipton[(61–65)], who provide sophisticated techniques to produce valid confidence intervals. Meta-analysts can use our R package to adjust their standard errors, export them, and then use them in more complex methods and packages[(3,4,63,64,66)]. With fewer than 30 studies researchers may consider using wild bootstrap.[(67)]

It is a good idea, and possible in the R package, to include study-level dummies (econometric fixed effects) to filter out study-specific idiosyncrasies related to unobserved heterogeneity. The package also reports a robust F-statistic of the first-stage regression. If the F-statistic is small, the instrument is weak and MAIVE results should be treated with caution.[(45)] Researchers may want to use confidence intervals robust to weak instruments.[(42–44)] In this respect, the package reports the Anderson-Rubin confidence interval for the estimate of the mean corrected effect.

The reader might object that our simulation is unfair to existing correction methods. The methods were designed to counter publication bias; we simulate p-hacking. Individual estimates and standard errors get biased, which is why selection models do not work well here—though they do not assume, as funnel methods assume, that selection works only on estimates (the Lombard effect discussed in the Introduction). The distinction between publication bias and p-hacking is clear in theory, but in practice both are often observationally equivalent to the meta-analyst. (But p-hacking likely predominates.[(68)]) As long as we believe our p-hacking environment is broadly realistic, we need a technique that corrects most of the resulting bias. MAIVE is such a technique. One can design p-hacking scenarios in which misspecifications make it almost impossible for meta-analysis methods to uncover the true unconditional mean.[(69,70)] If that is a realistic description of observational research, unconditional meta-analysis means are meaningless.[(71)] MAIVE can be extended to allow for observed heterogeneity and deliver context-specific means via incorporation into Bayesian model averaging meta-regression approaches addressing model uncertainty.[(24,72–75)]

S2 Supplementary Discussion: Empirical Applications

S2.1 Dataset 1: Kvarven et al. (2020)

Kvarven et al.[16] compare meta-analysis and replication results for influential primary studies in psychology. Each of the studies considered has given rise to an empirical literature that attempts to estimate the same effect, and for each literature there has been a meta-analysis. Kvarven et al. collect data from these meta-analyses and pair them with later preregistered multiple-laboratory replications. For 15 primary studies, Kvarven et al. are able to find both a meta-analysis and a preregistered multiple-laboratory replication. While the sample is admittedly small, a comparison of meta-analyses and replications is valuable because we can reasonably expect that preregistered multiple-laboratory replications are unlikely to suffer from publication bias or p-hacking: the results are published in any case. In other words, for each literature the replication result can serve as a proxy for the true underlying effect, not affected by selection. If a bias-correction meta-analysis technique provides results far from those of the multilab replication, we conclude that the technique is unlikely to work well. The original Kvarven et al. dataset does not include sample sizes for individual primary studies, and we need sample sizes for the MAIVE adjustment. We are grateful to Amanda Kvarven for providing us sample sizes for some of the primary studies; we collect the remaining sample sizes by contacting the authors of meta-analyses and inspecting individual primary studies.

The main finding of Kvarven et al. is that meta-analysis techniques yield estimates that are much larger than those of later multilab replications. The implication is that the literature suffers from p-hacking and publication bias and, at the same time, existing bias-correction techniques are unable to fully deal with the problem. Our intention is to find out whether MAIVE helps bring meta-analysis results closer to replication results. In doing so, we follow the approach of Kvarven et al. They use three meta-analysis estimators: PET-PEESE (as an example of a widely used funnel-based meta-regression technique), 3PSM (a widely used selection model), and Trim & Fill (one of the simplest existing correction techniques). We extend the analysis of Kvarven et al. by including the MAIVE version of each of those three estimators. We are most interested in the comparison of PET-PEESE, which was found by Kvarven et al. to have the smallest bias, with the MAIVE version of PET-PEESE without any weights (the version of MAIVE that we have preferred throughout the manuscript). The estimations of PET-PEESE and Trim & Fill, including the MAIVE versions, are conducted in Stata 18 and R. The code is provided in the replication files. The estimations of 3PSM, including the MAIVE version, are conducted using the program developed by Jack Vevea and Kathleen Coburn, which is available at `https://vevealab.shinyapps.io/WeightFunctionModel/`.

We recommend to use the MAIVE adjustment if minimal conditions for normal inference in regression are met and, at the same time, if inverse sample size is a reliably strong instrument for reported variance. Regarding the former, for MAIVE we require each meta-analysis to use at least 30 estimates from primary studies (which, among other things, gives the meta-analyst hope that the meta-regression confidence interval can be reliable). Regarding the latter, to be on the safe side in this application we require that the F-statistic from the first-stage regression, regressing reported variance on inverse sample size, is larger than 100. Keane and Neal (2023) [(76)] argue that the commonly used threshold of 10 is often not enough to ensure a strong instrument and valid inference. They recommend researchers use the Anderson-Rubin confidence interval, which is robust to weak instruments and ensures valid inference. In our R package `maive` we allow researchers to obtain this substantially more robust confidence interval so that they can use MAIVE with lower values of the F-statistics as well.

Table S7 shows the results. In 8 out of the 15 meta-analyses, both conditions for MAIVE (sample size and instrument strength) are met. Out of these 8 meta-analyses, in 6 cases (75%) is the MAIVE version of PET-PEESE with no weights (our preferred MAIVE specification throughout the manuscript) closer to the replication result than unadjusted PET-PEESE is. The MAIVE versions of Trim & Fill and 3PSM do not behave so well, though: in both cases, the majority of MAIVE estimates are farther from the replication values than the standard estimates are. Anyway, there is no strong theoretical basis to expect MAIVE improvements in these methods (like in p-uniform* and Andrews & Kasy), and the finding is also consistent with simulation results we have reported earlier. Table S7 also reports the first-stage F-statistic and the number of estimates (M) in the meta-analysis corresponding to each original study. The F statistic is generally pretty large, being larger than 10 in all cases, larger than 100 in all but 2, and larger than 1000 in most (8) of them. The sample sizes are not always very large, however: for instance, 5 meta-analyses have samples smaller than 30.

Table S8 reports the mean absolute deviations of each estimator, with and without the MAIVE adjustment, from the baseline (and arguably unbiased) preregistrated multilab replication result. Across all meta-analyses, the MAIVE version of PET-PEESE is, on average, slightly farther from the replication value than the standard version. This finding holds for both weighted and unweighted MAIVE variants. However, if we confine the comparison to meta-analyses with $F > 100$, with sample sizes satisfying $M > 30$, or both (the recommended minimum conditions for MAIVE), then the MAIVE versions of PET-PEESE, weighted or unweighted, are closer to the replication values than the standard PET-PEESE version is. Again, the MAIVE adjustment does not improve the performance of Trim & Fill and 3PSM, which is in line with our previous simulations for a selection model.

Table S7: Deviations of meta-analysis from replication results; based on Kvarven et al. (2020) [16]

Original study	Replication	Bias in PET-PEESE			Bias in Trim & Fill		Bias in 3PSM		F	M
		Standard	MAIVE (adj. weights)	MAIVE (no weights)	Standard	MAIVE	Standard	MAIVE		
Srull & Wyer (1979) [77]	0.063	0.09	0.11	0.06	0.32	0.29	0.36	0.39	25787	47
Graham et al. (2009) [78]	0.29	0.71	0.62	0.43	0.44	0.47	0.37	0.37	5604	32
Monin & Miller (2001) [79]	0.1466	0.25	0.22	0.19	0.08	0.08	0.10	0.08	4406	91
Sripada et al. (2014) [80]	0.04	0.05	0.11	0.14	0.44	0.46	0.46	0.70	1839	198
Tversky & Kahneman (1981) [81]	0.6	0.02	0.04	0.00	0.02	0.03	0.06	0.05	1249	80
Oppenheimer et al. (2009) [82]	0.27	0.05	0.01	0.39	0.23	0.24	0.03	0.03	852	100
Rand et al. (2012) [83]	-0.02	0.08	0.10	0.00	0.14	0.14	0.25	0.26	331	51
Husnu & Crisp (2010) [84]	0.13	0.48	0.26	0.37	0.21	0.23	0.17	0.20	151	32
Schwarz et al. (1991) [85]	-0.07	0.07	0.04	0.08	0.20	0.20	0.36	0.36	7237	16
Schooler & Engstler-Schooler (1990) [86]	0.171	0.08	0.13	0.21	0.07	0.07	0.02	0.01	2112	29
Mazar et al. (2008) [87]	-0.04	0.04	0.03	0.06	0.34	0.43	0.22	0.23	1960	15
Critcher & Gilovich (2008) [88]	0.04	0.33	0.38	0.40	0.26	0.28	0.19	0.08	269	21
Jostmann et al. (2009) [89]	0.03	0.11	0.09	0.13	0.48	0.50	0.18	0.30	104	25
Strack et al. (1988) [90]	0.016	0.04	0.13	0.14	0.21	0.23	0.26	0.55	81	286
Hauser et al. (2007) [91]	0.785	0.19	0.46	0.29	0.10	0.33	0.06	0.32	13	30

Notes: The first column denotes the original primary study; the second column shows the corresponding estimated standardized mean difference based on preregistered multilab replications; the next 7 columns show the absolute deviation of various meta-analysis methods relative to replications. Under the assumption that preregistered multilab replications are not affected by p-hacking and publication bias, the replications are unbiased estimates of the true effect, and the deviation of meta-analysis methods from replications measures bias in meta-analysis. F refers to the F-statistic in the first-stage regression of MAIVE. M is the sample size of the corresponding meta-analysis. We recommend using MAIVE only if $M > 30$ and, at the same time, $F > 100$. The first condition is necessary for standard inference in regression analysis. The second condition ensures that, in the particular literature, inverse sample size is a strong instrument for variance. These conditions are met for 8 replication–meta-analysis pairs shown in the first 8 rows; out of these, the MAIVE version of PET-PEESE without weights (the version of MAIVE that we have preferred throughout the paper) reduces the bias of unadjusted PET-PEESE in 6 cases. Trim & Fill and 3PSM are additionally shown in the table because Kvarven et al. consider them as well. MAIVE does not help improve the performance of these estimators, a result consistent with our previous simulations for the selection model.

Table S8: Mean absolute deviations of meta-analysis methods from replications

	PET-PEESE			Trim & Fill		3PSM	
	Standard	MAIVE (adj. weights)	MAIVE (no weights)	Standard	MAIVE	Standard	MAIVE
All datasets	0.17	0.18	0.19	0.24	0.26	0.21	0.26
$F > 100$	0.18	0.16	0.19	0.25	0.26	0.21	0.24
$M > 30$	0.20	0.18	0.19	0.23	0.24	0.23	0.29
$F > 100$ & $M > 30$	0.22	0.18	0.20	0.24	0.24	0.22	0.26

Notes: The table shows the mean absolute deviations of meta-analysis from replications. Under the assumption that preregistered multilab replications are not affected by p-hacking and publication bias, the replications are unbiased estimates of the true effect, and the deviation of meta-analysis methods from replications measures bias in meta-analysis. F is the F-statistic in the first-stage regression of MAIVE. M is the sample size of the corresponding meta-analysis. We recommend using MAIVE only if $M > 30$ and, at the same time, $F > 100$. The first condition is necessary for standard inference in regression analysis. The second condition ensures that, in the particular literature, inverse sample size is a strong instrument for variance. When these conditions are met, MAIVE reduces the mean absolute bias of PET-PEESE. Trim & Fill and 3PSM are additionally shown in the table because Kvarven et al. consider them as well. MAIVE does not help improve the performance of these estimators, a result consistent with our previous simulations for the selection model.

The bottom line is that MAIVE helps, to some extent, reduce the discrepancy between meta-analysis and replication results identified by Kvarven et al. If conditions for MAIVE in meta-regression analysis (strong instrument, at least modest sample size) are not met, MAIVE does not help but does not seem to hurt. However, because of the small dataset available for the comparison of meta-analyses and replications, we now turn to a richer database.

S2.2 Dataset 2: Bartos et al. (2024)

The comparison of meta-analyses and preregistered replications suggests that MAIVE can help bring meta-analysis results closer to the underlying effects, but the conclusions from such a comparison cannot be strong due to the limited number of the available meta-analysis–replication pairs for which the minimum MAIVE conditions are met. In this section we examine the stylized fact uncovered by Kvarven et al.: meta-analyses, even after correction for publication bias, tend to yield larger effect sizes than replications do. We investigate whether, in a large sample of meta-analyses of observational research (where spurious precision is likely to be especially strong), the MAIVE adjustment reduces the absolute value of the reported meta-analysis estimates. If that is the case, the implication is that, consistent with our p-hacking simulations, spurious precision creates an upward bias in existing meta-analysis correction techniques.

This second MAIVE application is performed on a large dataset compiled by Chris Doucouliagos and used in Bartos et al. (2024) [(92)]. The dataset comprises 613 meta-analyses (especially in economics, but also psychology, education, finance, business, political science, and sociology) and includes 209,766 estimates in total, almost exclusively those from observational studies. We thank Chris Doucouliagos for providing us the dataset: estimates and standard errors, also

Table S9: MAIVE estimates tend to be closer to 0 than PET-PEESE in economics meta-analyses

	All effect sizes		PET-PEESE significant	
	Absolute	%	Absolute	%
(a) All meta-analyses				
|MAIVE| > |PET-PEESE|	111	35.8	63	29.9
|MAIVE| < |PET-PEESE|	199	64.2	148	70.1
Total	310	100	193	100
(b) Meta-analyses with $F > 10$				
|MAIVE| > |PET-PEESE|	87	32.6	44	24.6
|MAIVE| < |PET-PEESE|	180	67.4	135	75.4
Total	267	100	172	100
(c) Meta-analyses with $F > 100$				
|MAIVE| > |PET-PEESE|	70	29.2	36	22.4
|MAIVE| < |PET-PEESE|	169	70.7	125	77.6
Total	239	100	151	100

Notes: The table compares the results of MAIVE and PET-PEESE for the sample of economics meta-analyses provided to us by Chris Doucouliagos.[(92)] The table separates the cases in which the estimated underlying effect in PET-PEESE is statistically significant at the 5% level (right) and when all PET-PEESE estimates are considered (left). In both cases, MAIVE estimates are typically closer to zero (that is, smaller in absolute value) than PET-PEESE estimates. The difference is larger for statistically significant effects and for meta-analyses with a large F-statistic in the first-stage regression of MAIVE.

with sample sizes in some cases. Because sample sizes are crucial for the MAIVE adjustment, we attempt to collect them for as many primary studies as possible. We restrict our analysis to (1) meta-analyses with at least 30 estimates ($M > 30$), (2) estimates based on at least 10 observations, (3) estimates with available sample sizes. Doing so reduces the set of usable meta-analyses to 348. We further censor the data at the 1% and 99% percentiles to limit the influence of extreme outliers. For each of the 348 meta-analyses we apply PET-PEESE and its MAIVE version. In 38 meta-analyses, a negative slope estimate appears in the first-stage regression, implying that sample sizes are positively rather than negatively correlated with standard errors. These meta-analyses are also discarded, resulting in a final set of 310 meta-analyses. Computations are conducted in Matlab and R; codes are available at `meta-analysis.cz/maive`.

If SE-selection is present, based on our previous p-hacking simulations we expect MAIVE to reduce the estimates of PET-PEESE. This means that MAIVE should be less negative than PET-PEESE when PET-PEESE is negative, and less positive when PET-PEESE is positive. Table S9 displays the results of such a comparison. Panel (a) considers all the 310 meta-analyses for which the first-stage slope coefficient is positive. MAIVE produces smaller estimates than PET-PEESE in absolute value in 64.2% of the cases and larger estimates in 35.8% of the cases. Among the meta-analyses where the PET-PEESE is statistically significant at the 5% level (the right-hand portion of the table), the difference is greater: 70.1% vs. 29.9%.

Panel (b) restricts the count to meta-analyses where the first-stage F is larger than 10, which

Fig. S10: Histograms of the percentage change of MAIVE relative to unadjusted PET-PEESE

(a): All meta-analyses (310)

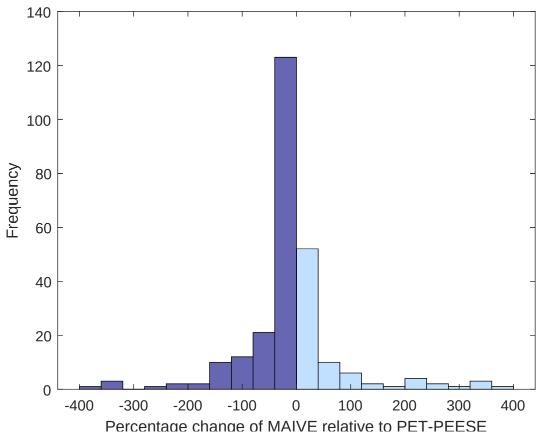

(b): Meta-analyses with $F > 10$ (267)

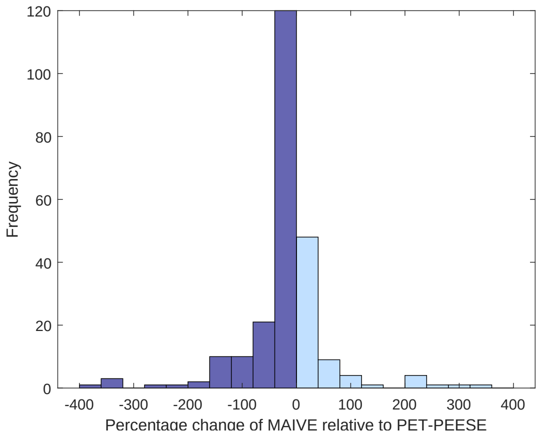

(c): Meta-analyses with $F > 100$ (239)

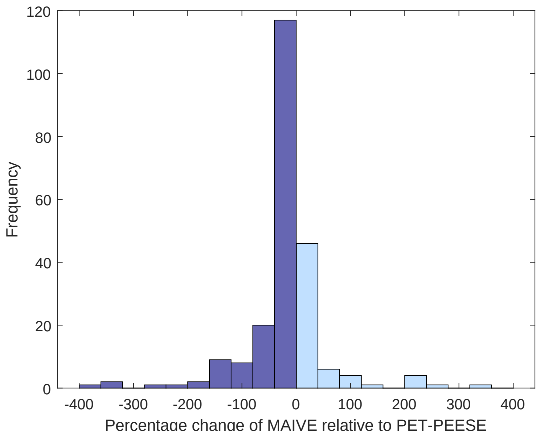

Notes: The figure compares the results, in absolute value, of MAIVE and PET-PEESE for the sample of economics meta-analyses provided to us by Chris Doucouliagos. [92] All available meta-analyses are used, including those that exhibit insignificant results for PET-PEESE. In most cases, MAIVE adjustment reduces PET-PEESE estimates closer to zero. The difference is larger for meta-analyses with a large F-statistic in the first-stage regression of MAIVE.

amount to 267. Here the tendency of MAIVE to reduce PET-PEESE estimates is even more evident: MAIVE reduces PET-PEESE estimates in 67.4% of the cases when all meta-analyses are considered and in 75.4% of the cases when only meta-analyses with statistically significant PET-PEESE estimates are considered. Stronger still are the results in Panel (c), which only considers the 239 meta-analyses with $F > 100$, where inverse sample size forms a very strong instrument for reported variance. Here, MAIVE reduces PET-PEESE estimates in 70.7% of the cases when all meta-analyses are considered and in 77.6% of the cases when only meta-analyses with statistically significant PET-PEESE estimates are considered.

MAIVE corrects down PET-PEESE more strongly when the first-stage F-statistic is large. It is also important to emphasize how likely it is that sample size is a strong instrument for standard errors: in 267 of the 310 meta-analyses for which a first-stage regression is computed (i.e., 86%), the first-stage F statistic exceeds 10. And in 239 of those (i.e., 77%), the F statistic is larger than 100.

Finally, Figure S10 plots the histogram of the percentage change of MAIVE relative to PET-PEESE when all meta-analyses are considered (whether or not PET-PEESE estimates are significant). Dark blue bars indicate negative changes, i.e. meta-analyses for which MAIVE is smaller than a positive PET-PEESE estimate or higher than a negative PET-PEESE estimate. The dark bars should dominate if MAIVE is to correct down PET-PEESE estimates. Light blue bars, in contrast, correspond to meta-analyses for which MAIVE exacerbate a PET-PEESE estimate. Panel (a) shows the histogram considering all 310 meta-analyses for which a positive first-stage coefficient is estimated. Clearly, and consistent with the results in Table S9, MAIVE corrects down most PET-PEESE estimates in absolute value. This result is clearer if we only consider the 267 meta-analyses with $F > 10$ (Panel (b)) and, clearer still, the 239 meta-analyses with $F > 100$.

The bottom line of the second application is the following: we find strong evidence that MAIVE typically reduces PET-PEESE estimates (in about 70% of the cases, which is similar to the results for the Kvarven et al. dataset). The finding is consistent with the conclusion that (i) spurious precision is important and works in a way qualitatively similar to our p-hacking simulations, and (ii) spurious precision helps explain the fact, uncovered by Kvarven et al., that conventional techniques for bias correction in meta-analysis produce effect sizes that are too large compared to preregistered multiple-laboratory replications.

S3 Simulations with True Effect Heterogeneity

Fig. S11: Bias: p-hacking selection, no effect ($\alpha_1 = 0$), various values of ψ: allowing for true effect heterogeneity ($\sigma^2_{\alpha_1} = 0.64$)

(a) Unadjusted estimators

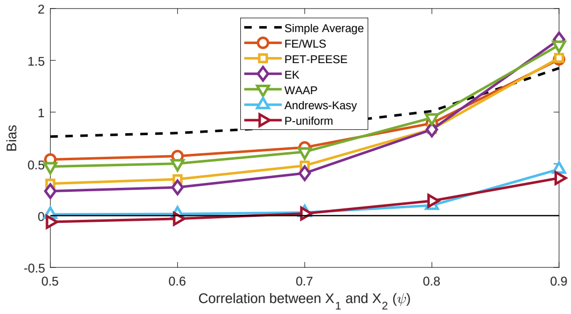

(b) Adjusting FE/WLS

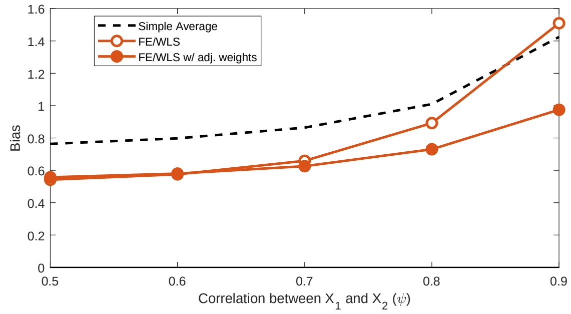

(c) Adjusting PET-PEESE

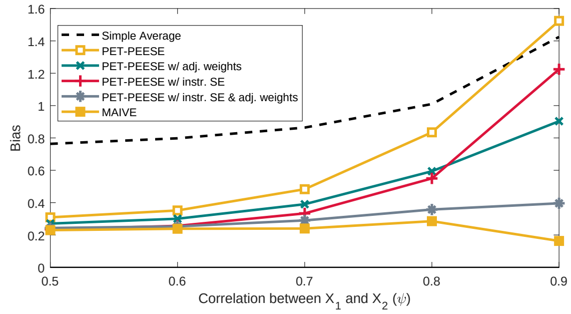

(d) Adjusting EK

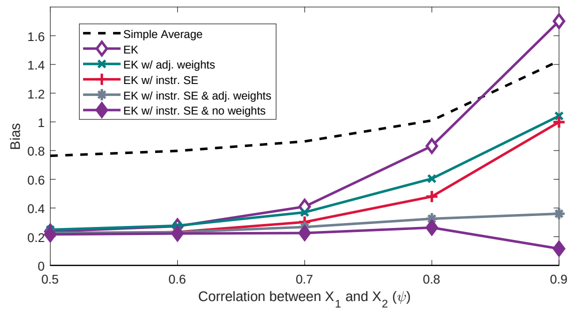

(e) Adjusting WAAP

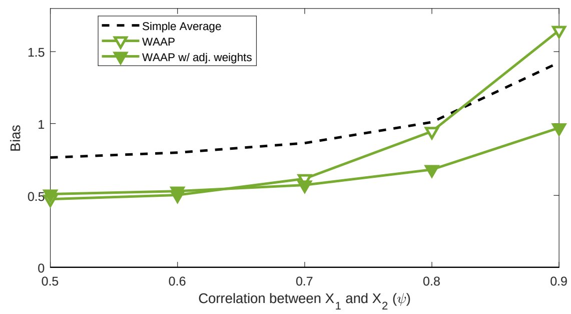

(f) Adjusting Andrews-Kasy

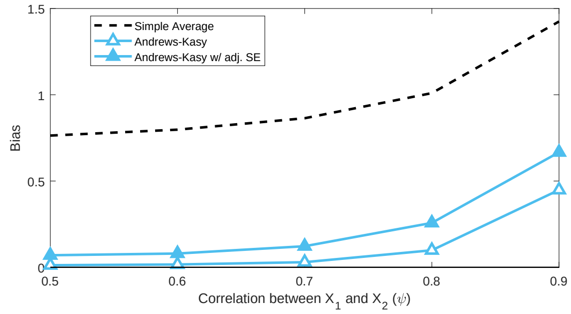

(g) Adjusting p-uniform*

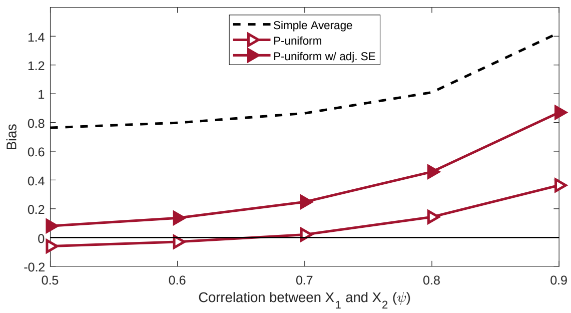

(h) Comparison of adjusted estimators

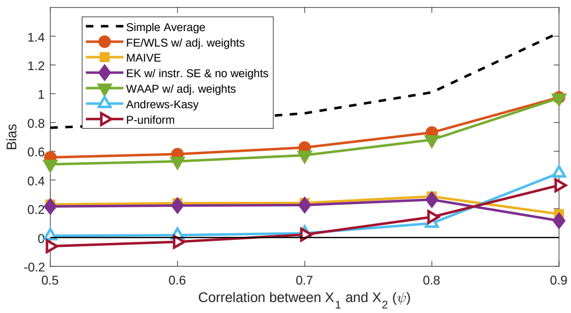

Notes: Figure shows the bias for the case of no effect in the p-hacking scenario allowing for true effect heterogeneity. Panels show (a) a comparison of biases for all unadjusted estimators; bias for (b) the fixed effects or weighted least squares estimator with adjustment, (c) the adjusted precision-effect test and precision-effect estimate with standard errors, (d) the adjusted endogenous kink estimator, (e) the adjusted weighted average of adequately powered; (f) the adjusted Andrews and Kasy estimator, (g) the adjusted p-uniform* method; and (h) a comparison of biases for all adjusted estimators.

Fig. S12: MSE: P-hacking selection, no effect ($\alpha_1 = 0$), various values of ψ: allowing for true effect heterogeneity ($\sigma^2_{\alpha_1} = 0.64$)

(a) Unadjusted estimators

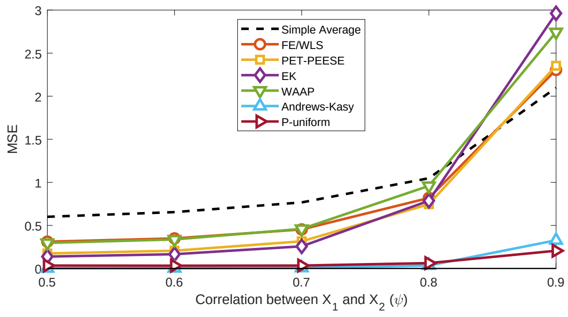

(b) Adjusting FE/WLS

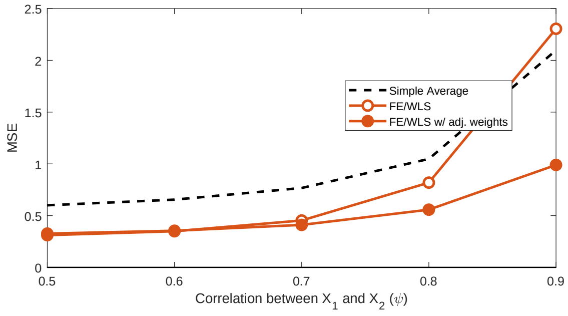

(c) Adjusting PET-PEESE

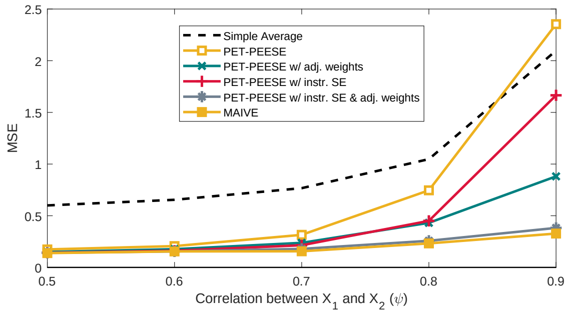

(d) Adjusting EK

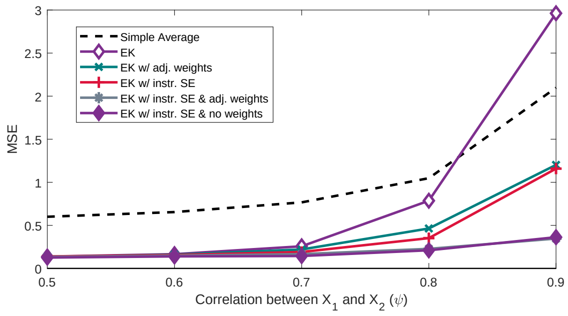

(e) Adjusting WAAP

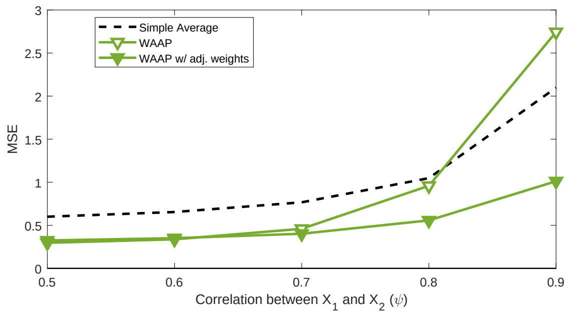

(f) Adjusting Andrews-Kasy

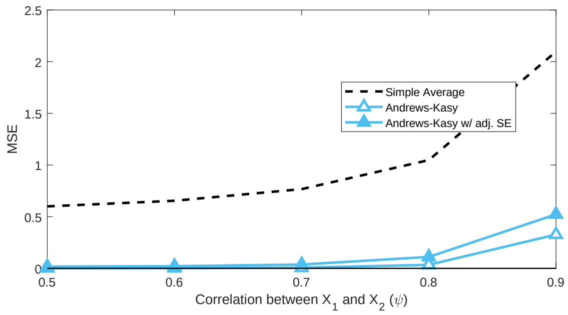

(g) Adjusting *p*-uniform*

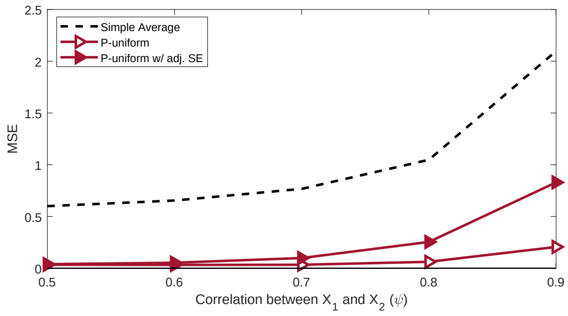

(h) Comparison of adjusted estimators

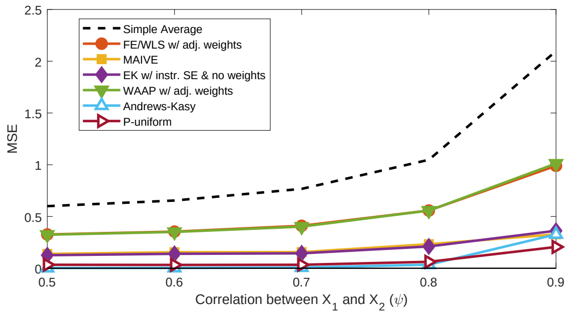

Notes: Figure shows the mean squared error (MSE) for the case of no effect in the *p*-hacking scenario allowing for true effect heterogeneity. Panels show (a) a comparison of MSEs for all unadjusted estimators; MSE for (b) the fixed effects or weighted least squares estimator with adjustment, (c) the adjusted precision-effect test and precision-effect estimate with standard errors, (d) the adjusted endogenous kink estimator, (e) the adjusted weighted average of adequately powered; (f) the adjusted Andrews and Kasy estimator, (g) the adjusted p-uniform* method; and (h) a comparison of MSEs for all adjusted estimators.

Fig. S13: Coverage: P-hacking selection, no effect ($\alpha_1 = 0$), various values of ψ: allowing for true effect heterogeneity ($\sigma^2_{\alpha_1} = 0.64$)

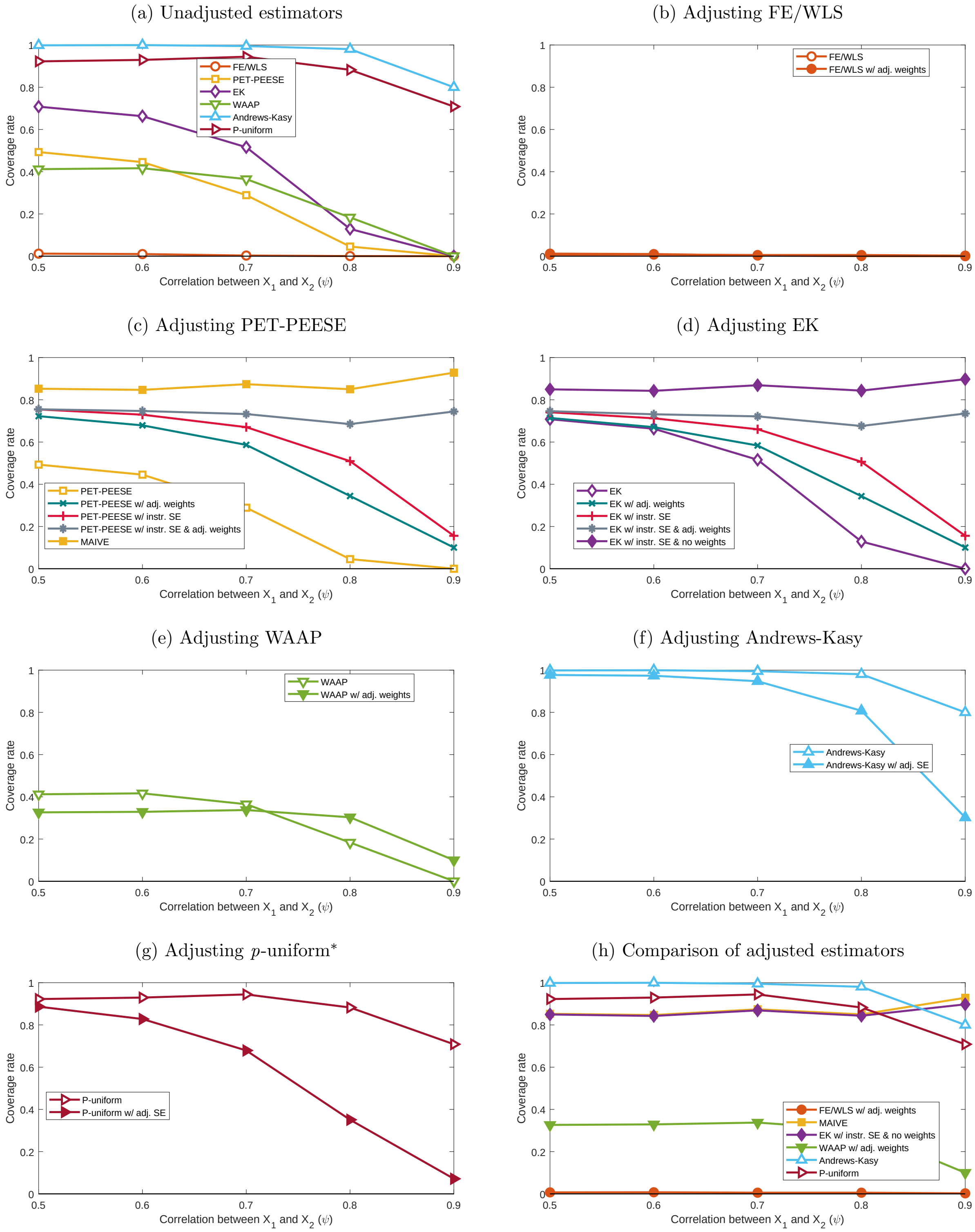

Notes: Figure shows the coverage rate for the case of no effect in the p-hacking scenario allowing for true effect heterogeneity. Panels show (a) a comparison of coverage rates for all unadjusted estimators; coverage rate for (b) the fixed effects or weighted least squares estimator with adjustment, (c) the adjusted precision-effect test and precision-effect estimate with standard errors, (d) the adjusted endogenous kink estimator, (e) the adjusted weighted average of adequately powered; (f) the adjusted Andrews and Kasy estimator, (g) the adjusted p-uniform* method; and (h) a comparison of coverage rates for all adjusted estimators.

Fig. S14: Bias: P-hacking selection, positive effect ($\alpha_1 = 1$), various values of ψ: allowing for true effect heterogeneity ($\sigma^2_{\alpha_1} = 0.64$)

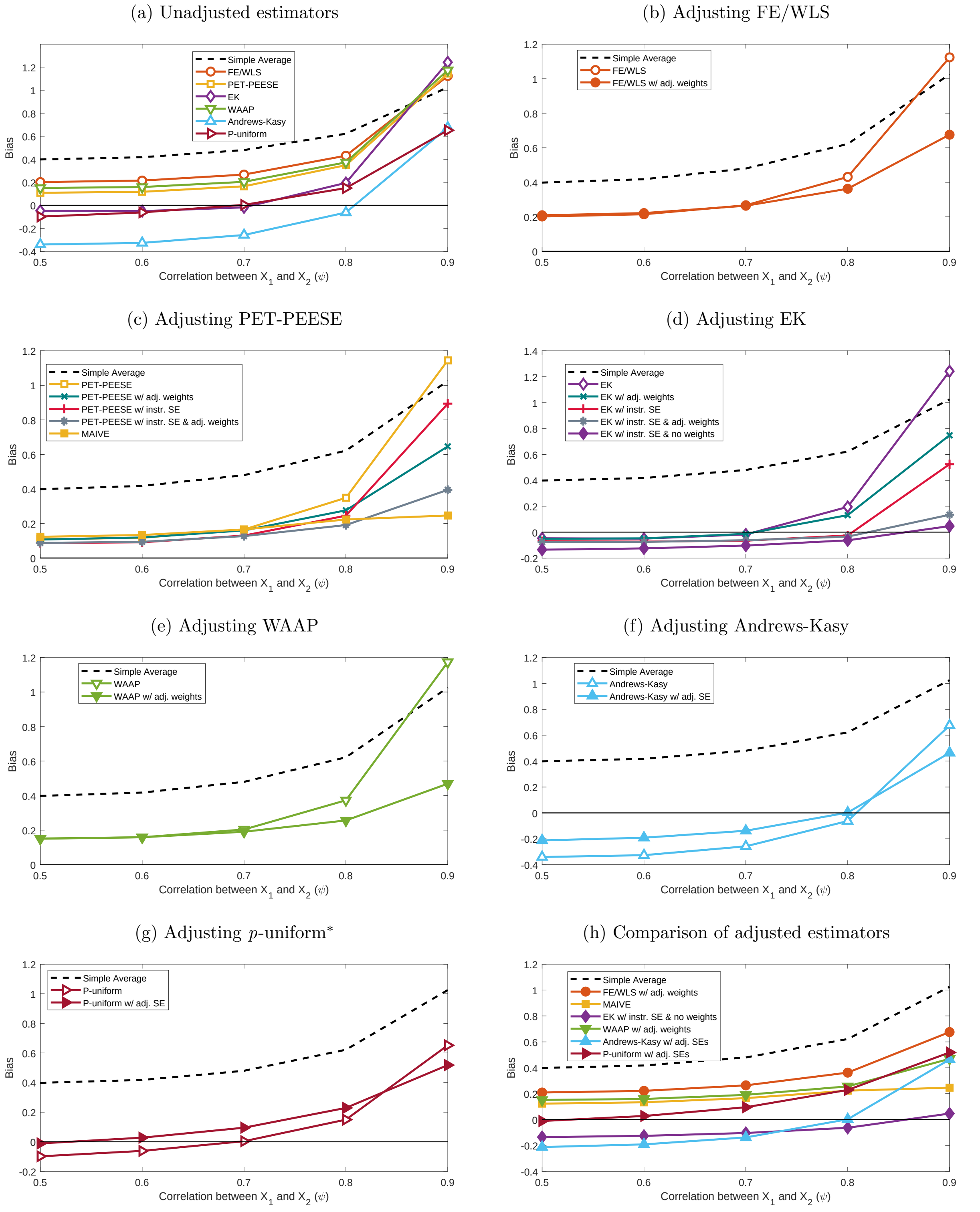

Notes: Figure shows the bias for the case of a positive effect in the p-hacking scenario allowing for true effect heterogeneity. Panels show (a) a comparison of biases for all unadjusted estimators; bias for (b) the fixed effects or weighted least squares estimator with adjustment, (c) the adjusted precision-effect test and precision-effect estimate with standard errors, (d) the adjusted endogenous kink estimator, (e) the adjusted weighted average of adequately powered; (f) the adjusted Andrews and Kasy estimator, (g) the adjusted p-uniform* method; and (h) a comparison of biases for all adjusted estimators.

Fig. S15: MSE: P-hacking selection, positive effect ($\alpha_1 = 1$), various values of ψ: allowing for true effect heterogeneity ($\sigma^2_{\alpha_1} = 0.64$)

(a) Unadjusted estimators

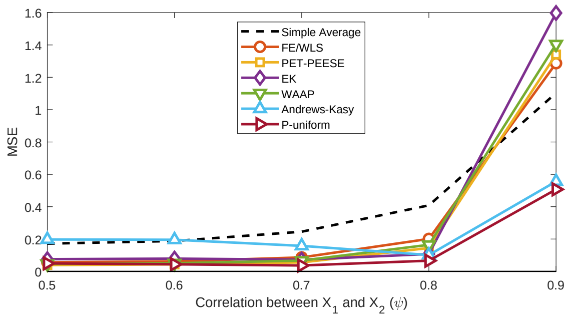

(b) Adjusting FE/WLS

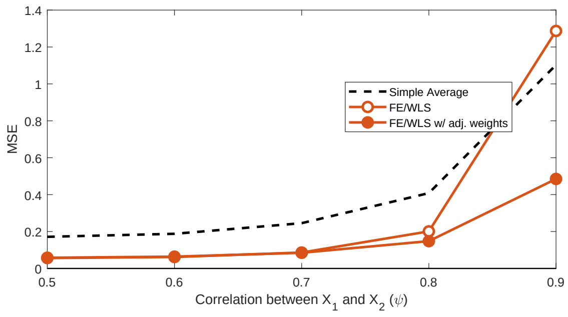

(c) Adjusting PET-PEESE

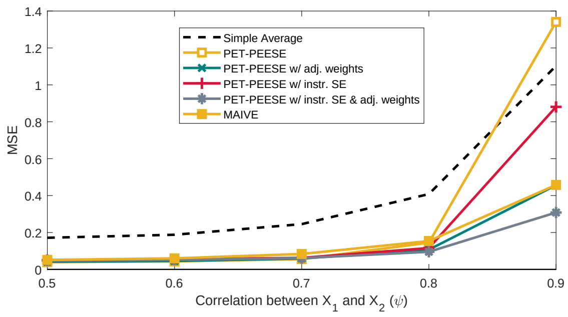

(d) Adjusting EK

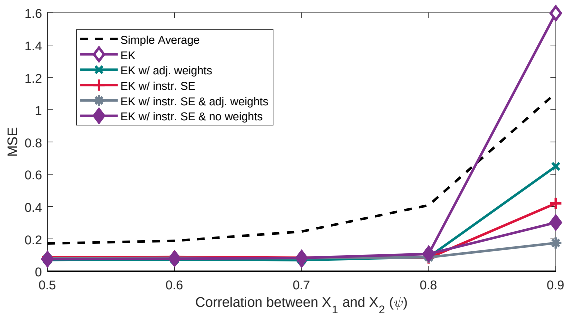

(e) Adjusting WAAP

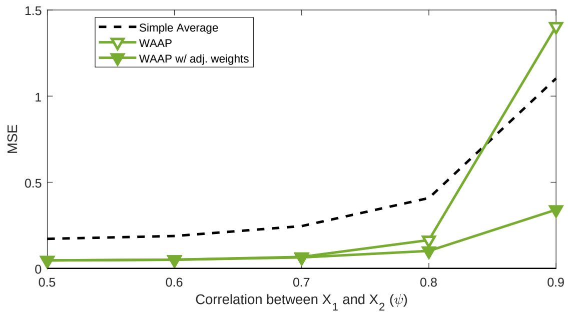

(f) Adjusting Andrews-Kasy

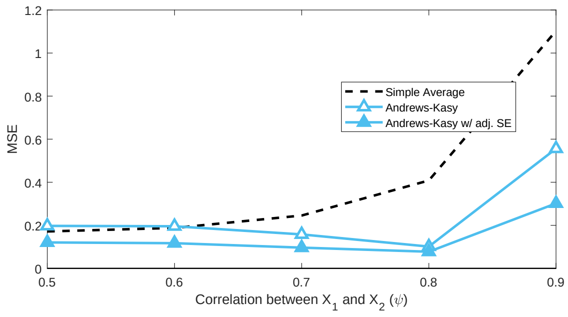

(g) Adjusting *p*-uniform*

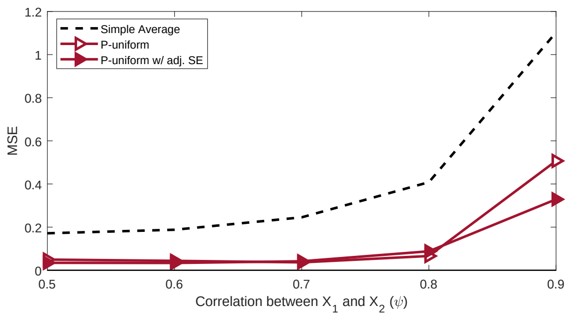

(h) Comparison of adjusted estimators

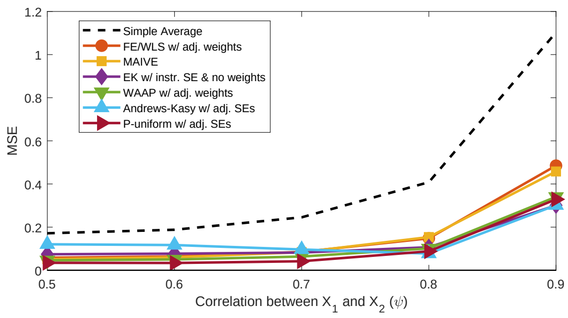

Notes: Figure shows the mean squared error (MSE) for the case of a positive effect in the *p*-hacking scenario allowing for true effect heterogeneity. Panels show (a) a comparison of MSEs for all unadjusted estimators; MSE for (b) the fixed effects or weighted least squares estimator with adjustment, (c) the adjusted precision-effect test and precision-effect estimate with standard errors, (d) the adjusted endogenous kink estimator, (e) the adjusted weighted average of adequately powered; (f) the adjusted Andrews and Kasy estimator, (g) the adjusted p-uniform* method; and (h) a comparison of MSEs for all adjusted estimators.

Fig. S16: Coverage: P-hacking selection, positive effect ($\alpha_1 = 1$), various values of ψ: allowing for true effect heterogeneity ($\sigma^2_{\alpha_1} = 0.64$)

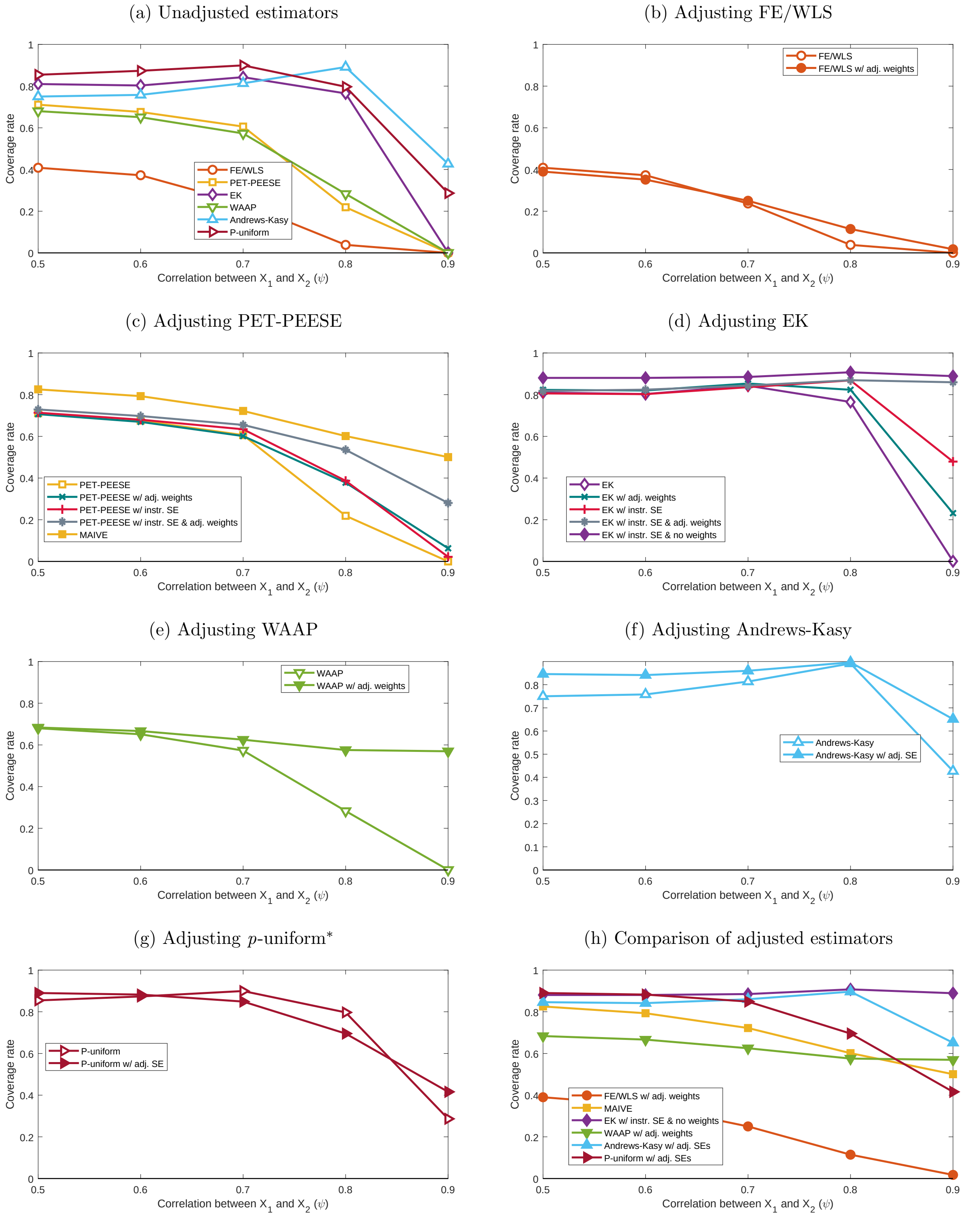

Notes: Figure shows the coverage rate for the case of a positive effect in the p-hacking scenario allowing for true effect heterogeneity. Panels show (a) a comparison of coverage rates for all unadjusted estimators; coverage rate for (b) the fixed effects or weighted least squares estimator with adjustment, (c) the adjusted precision-effect test and precision-effect estimate with standard errors, (d) the adjusted endogenous kink estimator, (e) the adjusted weighted average of adequately powered; (f) the adjusted Andrews and Kasy estimator, (g) the adjusted p-uniform* method; and (h) a comparison of coverage rates for all adjusted estimators.

S4 Simulations with Small Meta-Sample Sizes

Fig. S17: Bias: P-hacking selection, no effect ($\alpha_1 = 0$), various values of ψ: small meta-sample sizes ($M = 30$)

(a) Unadjusted estimators

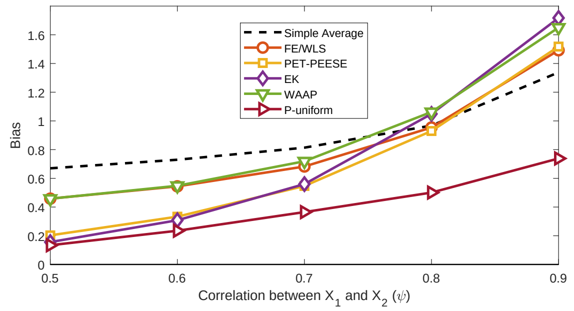

(b) Adjusting FE/WLS

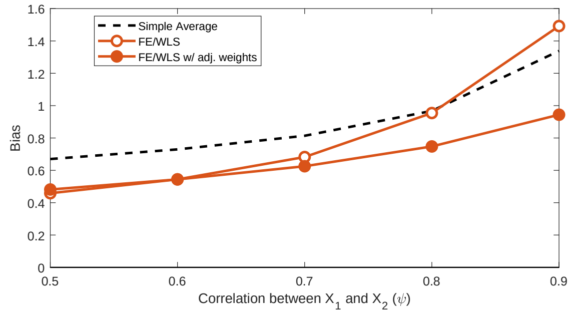

(c) Adjusting PET-PEESE

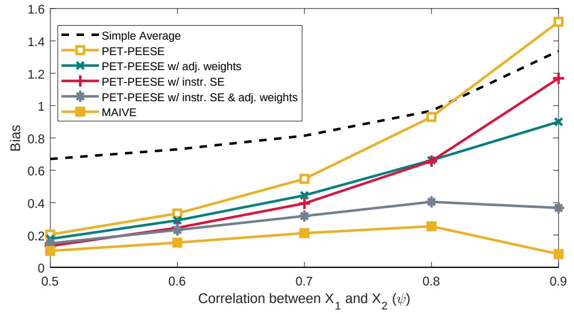

(d) Adjusting EK

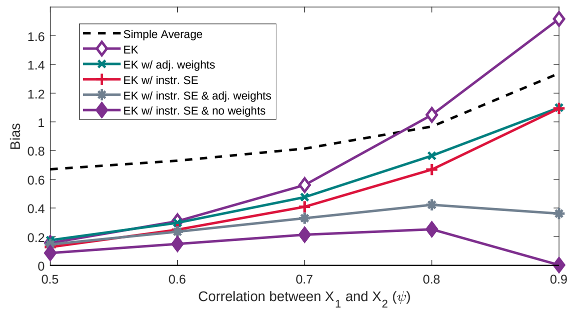

(e) Adjusting WAAP

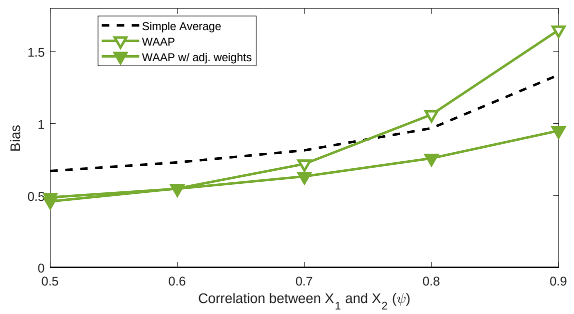

(f) Adjusting Andrews-Kasy

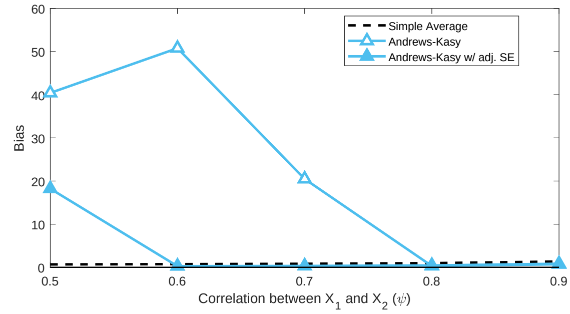

(g) Adjusting p-uniform*

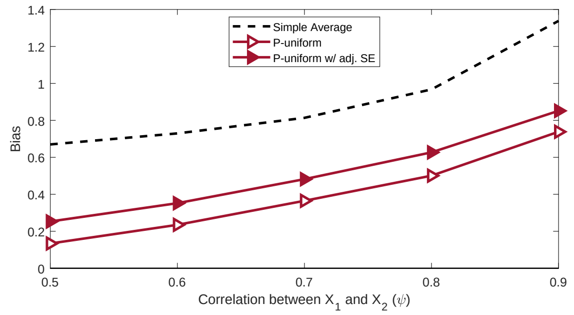

(h) Comparison of adjusted estimators

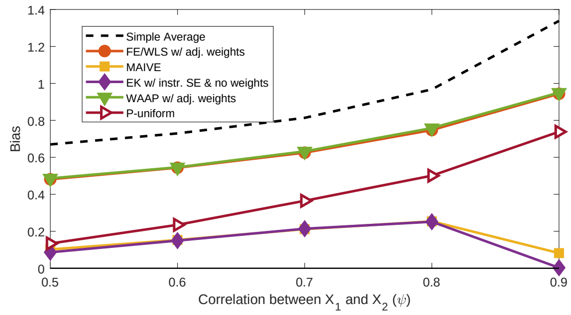

Notes: Figure shows the bias for the case of no effect in the p-hacking scenario considering smaller meta-samples. Panels show (a) a comparison of biases for all unadjusted estimators; bias for (b) the fixed effects or weighted least squares estimator with adjustment, (c) the adjusted precision-effect test and precision-effect estimate with standard errors, (d) the adjusted endogenous kink estimator, (e) the adjusted weighted average of adequately powered; (f) the adjusted Andrews and Kasy estimator, (g) the adjusted p-uniform* method; and (h) a comparison of biases for all adjusted estimators.

Fig. S18: MSE: P-hacking selection, no effect ($\alpha_1 = 0$), various values of ψ: small meta-sample sizes ($M = 30$)

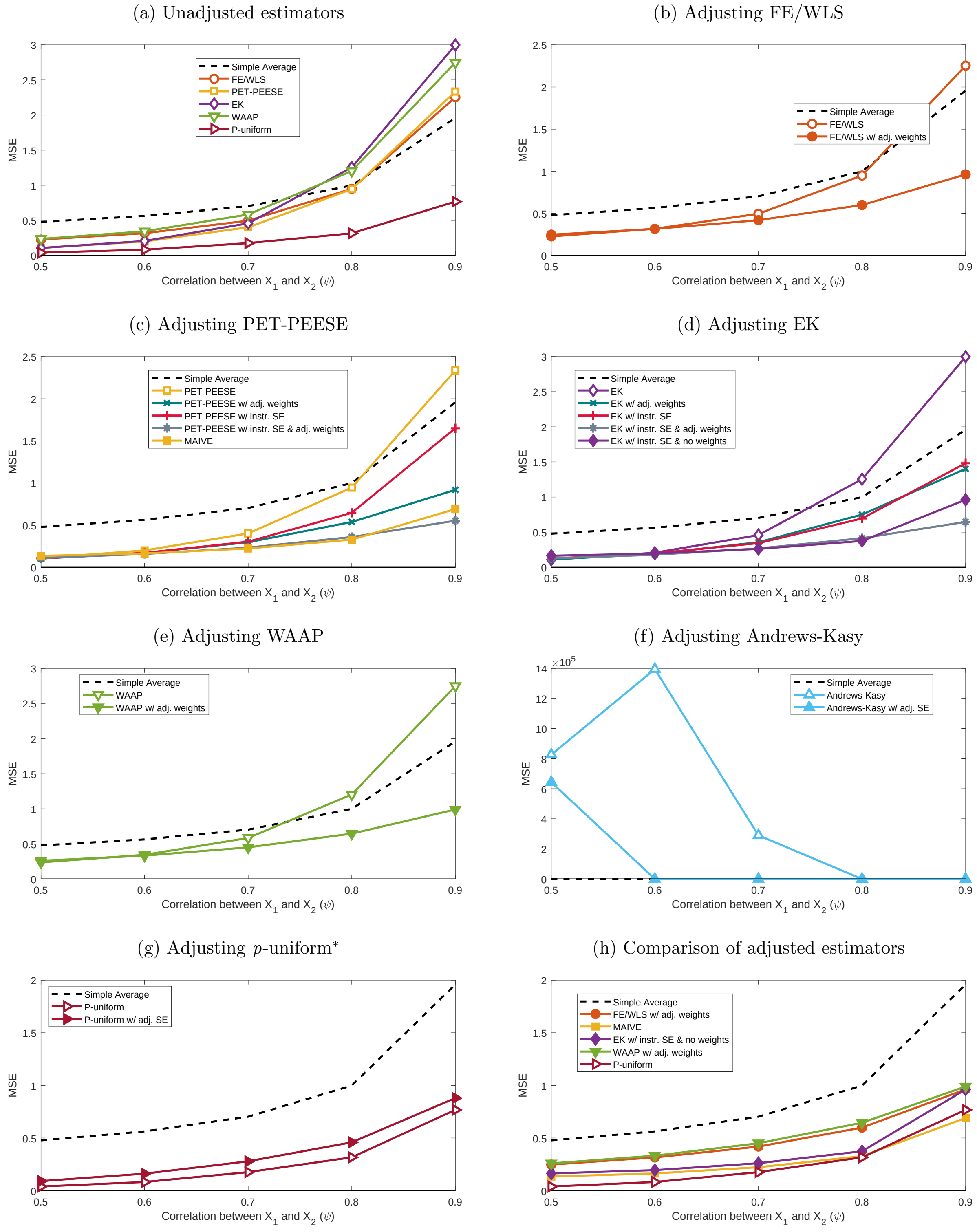

Notes: Figure shows the mean squared error (MSE) for the case of no effect in the p-hacking scenario considering smaller meta-samples. Panels show (a) a comparison of MSEs for all unadjusted estimators; MSE for (b) the fixed effects or weighted least squares estimator with adjustment, (c) the adjusted precision-effect test and precision-effect estimate with standard errors, (d) the adjusted endogenous kink estimator, (e) the adjusted weighted average of adequately powered; (f) the adjusted Andrews and Kasy estimator, (g) the adjusted p-uniform* method; and (h) a comparison of MSEs for all adjusted estimators.

Fig. S19: Coverage: P-hacking selection, no effect ($\alpha_1 = 0$), various values of ψ: small meta-sample sizes ($M = 30$)

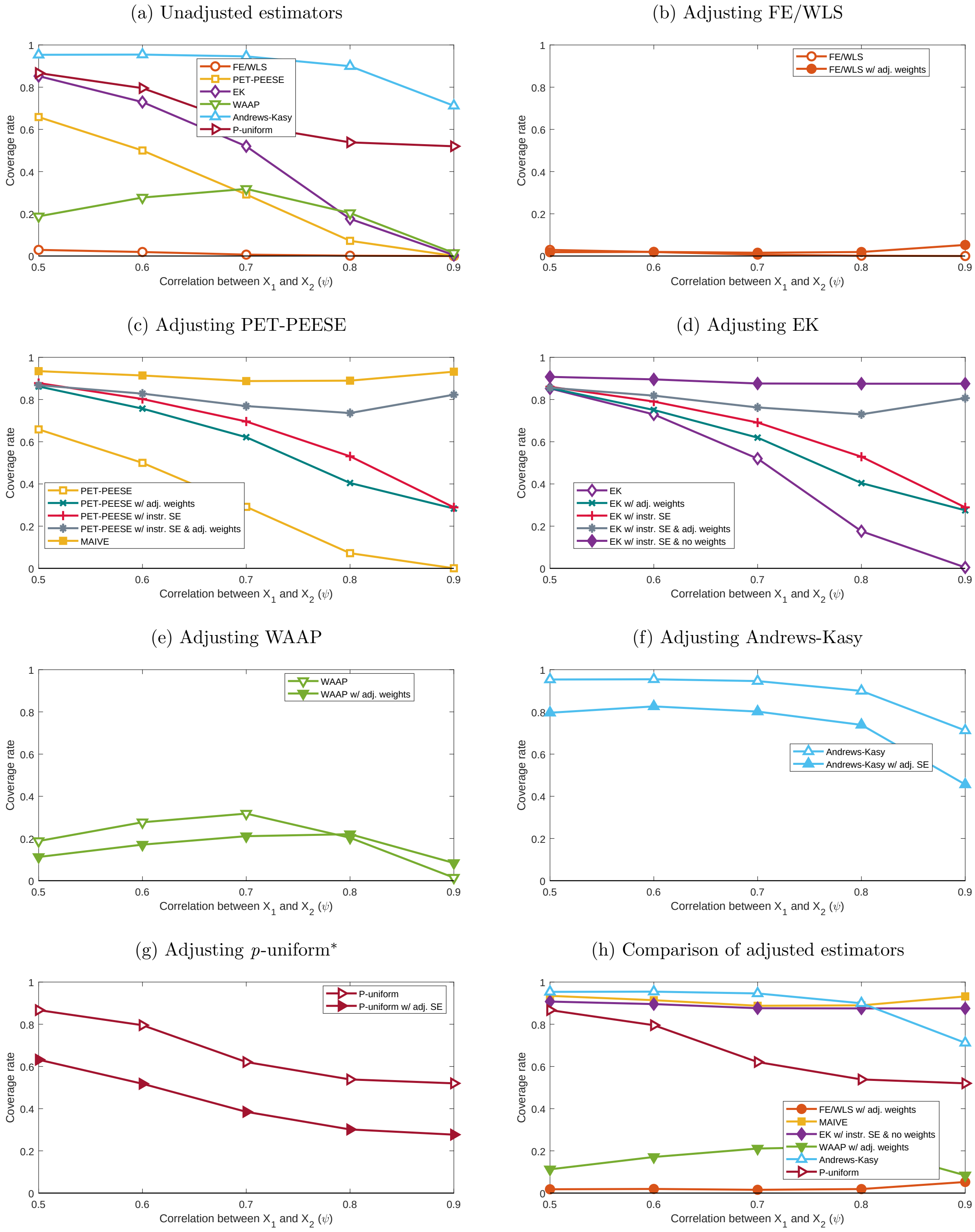

Notes: Figure shows the coverage rate for the case of no effect in the p-hacking scenario considering smaller meta-samples. Panels show (a) a comparison of coverage rates for all unadjusted estimators; coverage rate for (b) the fixed effects or weighted least squares estimator with adjustment, (c) the adjusted precision-effect test and precision-effect estimate with standard errors, (d) the adjusted endogenous kink estimator, (e) the adjusted weighted average of adequately powered; (f) the adjusted Andrews and Kasy estimator, (g) the adjusted p-uniform* method; and (h) a comparison of coverage rates for all adjusted estimators.

Fig. S20: Bias: P-hacking selection, positive effect ($\alpha_1 = 1$), various values of ψ: small meta-sample sizes ($M = 30$)

(a) Unadjusted estimators

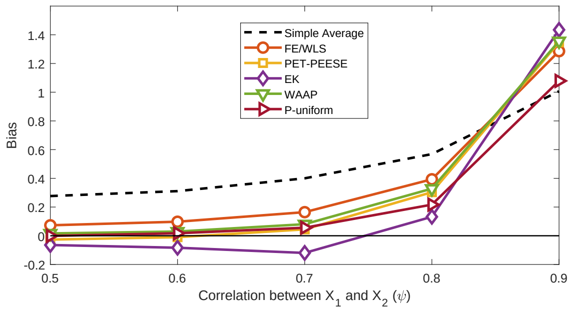

(b) Adjusting FE/WLS

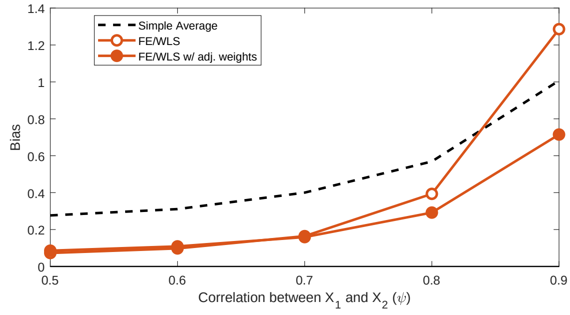

(c) Adjusting PET-PEESE

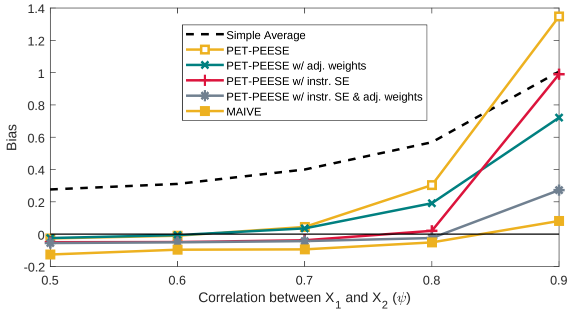

(d) Adjusting EK

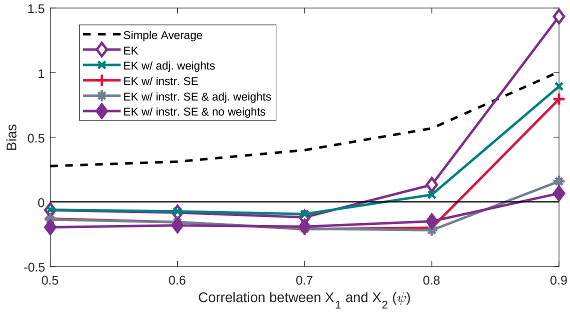

(e) Adjusting WAAP

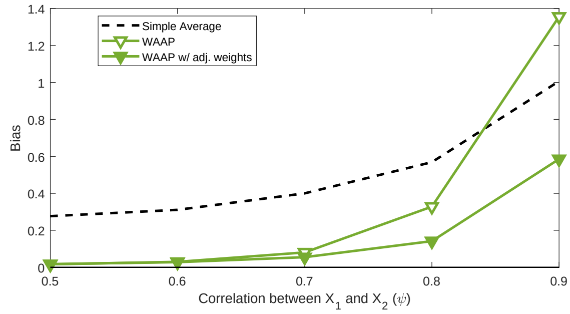

(f) Adjusting Andrews-Kasy

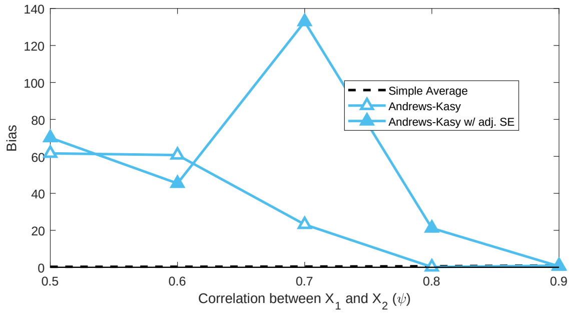

(g) Adjusting p-uniform*

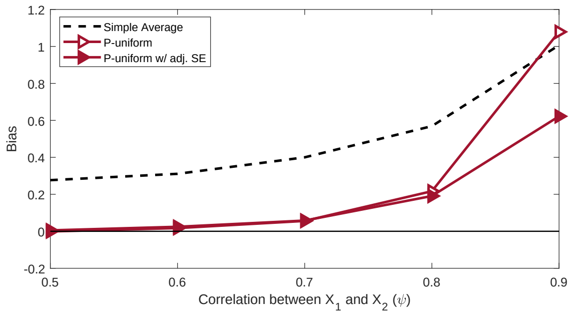

(h) Comparison of adjusted estimators

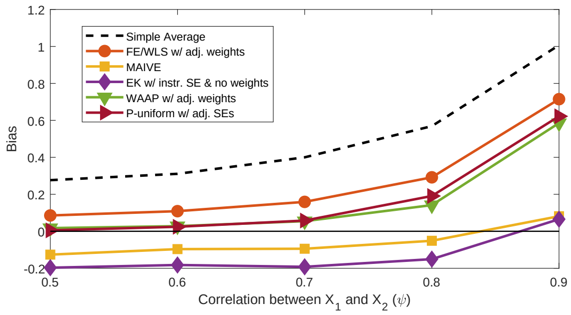

Notes: Figure shows the bias for the case of a positive effect in the p-hacking scenario considering smaller meta-samples. Panels show (a) a comparison of biases for all unadjusted estimators; bias for (b) the fixed effects or weighted least squares estimator with adjustment, (c) the adjusted precision-effect test and precision-effect estimate with standard errors, (d) the adjusted endogenous kink estimator, (e) the adjusted weighted average of adequately powered; (f) the adjusted Andrews and Kasy estimator, (g) the adjusted p-uniform* method; and (h) a comparison of biases for all adjusted estimators.

Fig. S21: MSE: P-hacking selection, positive effect ($\alpha_1 = 1$), various values of ψ: small meta-sample sizes ($M = 30$)

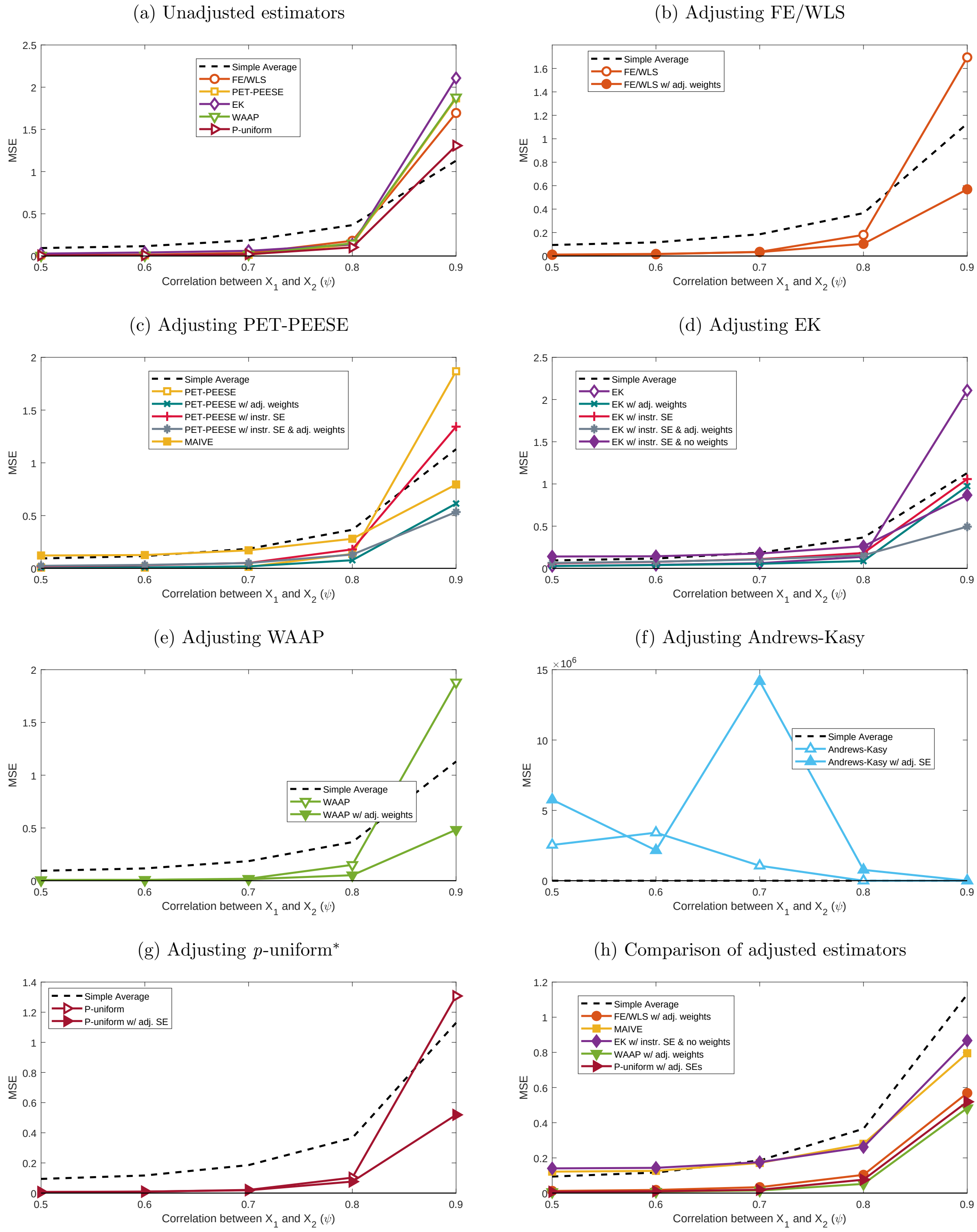

Notes: Figure shows the mean squared error (MSE) for the case of a positive effect in the *p*-hacking scenario considering smaller meta-samples. Panels show (a) a comparison of MSEs for all unadjusted estimators; MSE for (b) the fixed effects or weighted least squares estimator with adjustment, (c) the adjusted precision-effect test and precision-effect estimate with standard errors, (d) the adjusted endogenous kink estimator, (e) the adjusted weighted average of adequately powered; (f) the adjusted Andrews and Kasy estimator, (g) the adjusted p-uniform* method; and (h) a comparison of MSEs for all adjusted estimators.

Fig. S22: Coverage: P-hacking selection, positive effect ($\alpha_1 = 1$), various values of ψ: small meta-sample sizes ($M = 30$)

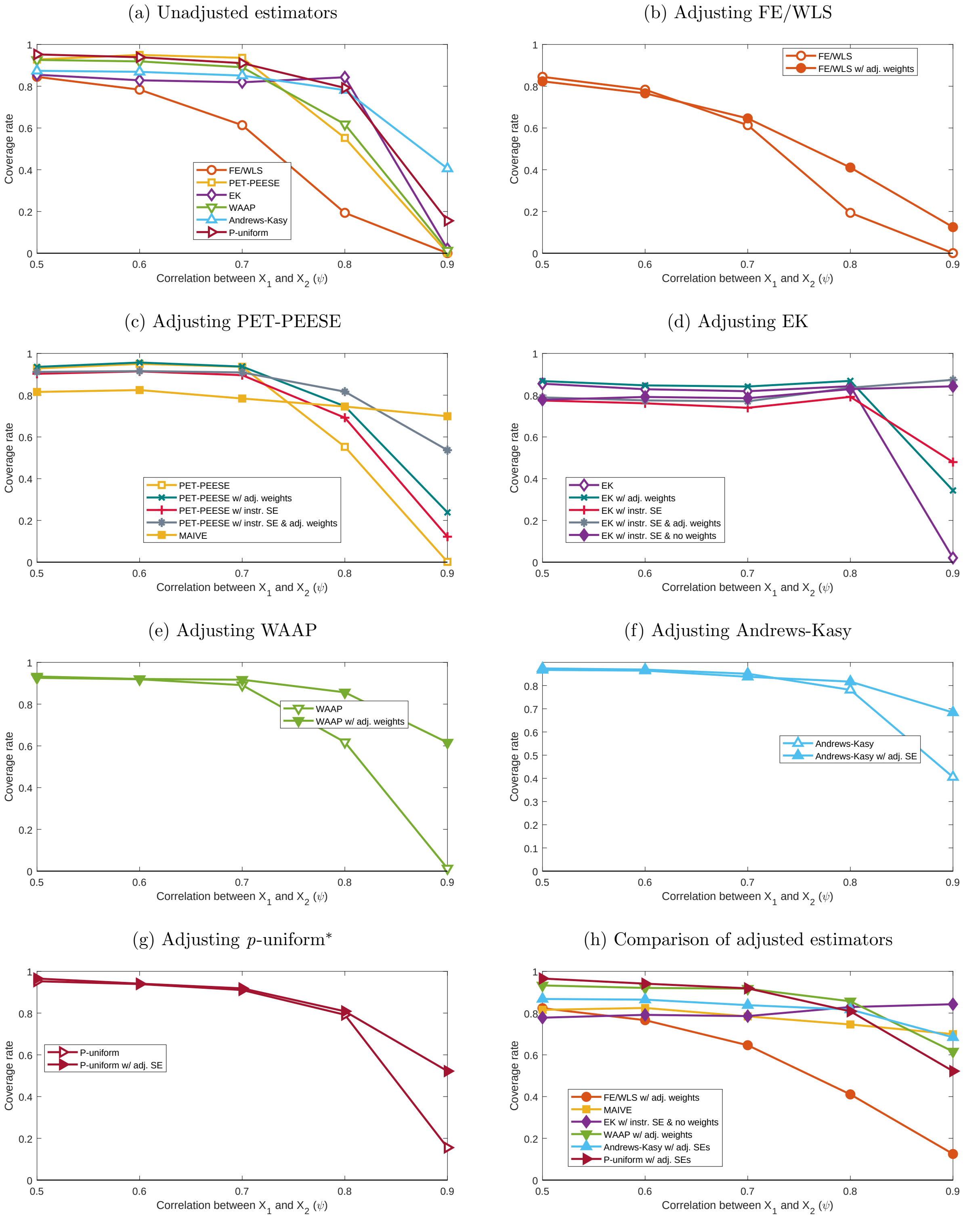

Notes: Figure shows the coverage rate for the case of a positive effect in the p-hacking scenario considering smaller meta-samples. Panels show (a) a comparison of coverage rates for all unadjusted estimators; coverage rate for (b) the fixed effects or weighted least squares estimator with adjustment, (c) the adjusted precision-effect test and precision-effect estimate with standard errors, (d) the adjusted endogenous kink estimator, (e) the adjusted weighted average of adequately powered; (f) the adjusted Andrews and Kasy estimator, (g) the adjusted p-uniform* method; and (h) a comparison of coverage rates for all adjusted estimators.

S5 Stylized Scenario for a Large Underlying Effect

Fig. S23: Bias: stylized scenario, large effect ($\alpha_1 = 2$)

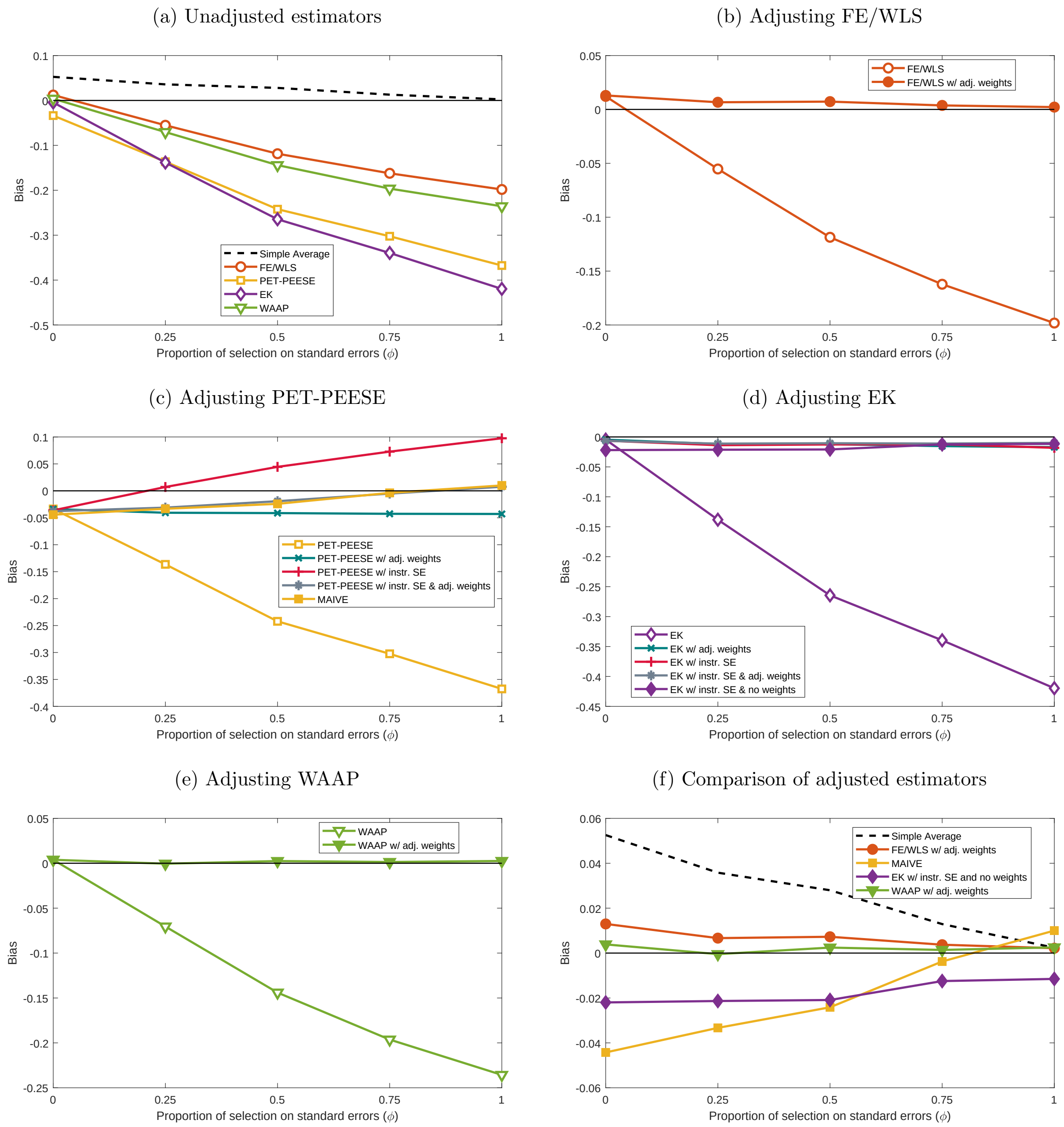

Notes: Figure shows the bias for the case of a large effect in our stylized scenario. Panels show (a) a comparison of biases for all unadjusted estimators; bias for (b) the fixed effects or weighted least squares estimator with adjustment, (c) the adjusted precision-effect test and precision-effect estimate with standard errors, (d) the adjusted endogenous kink estimator, (e) the adjusted weighted average of adequately powered; and (f) a comparison of biases for all adjusted estimators.

Fig. S24: MSE: stylized scenario, large effect ($\alpha_1 = 2$)

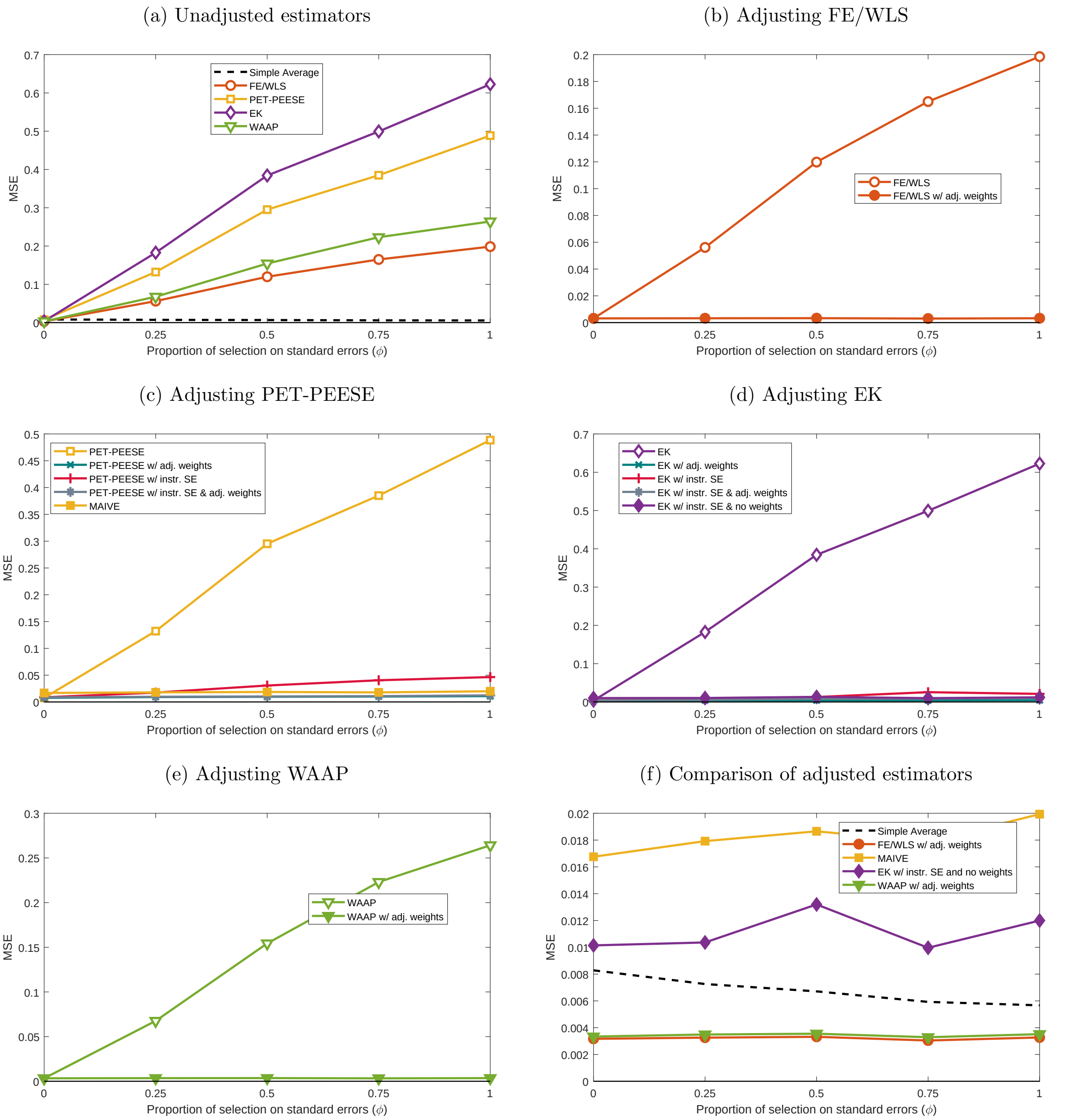

Notes: Figure shows the mean squared error (MSE) for the case of a large effect in our stylized scenario. Panels show (a) a comparison of MSEs for all unadjusted estimators; MSE for (b) the fixed effects or weighted least squares estimator with adjustment, (c) the adjusted precision-effect test and precision-effect estimate with standard errors, (d) the adjusted endogenous kink estimator, (e) the adjusted weighted average of adequately powered; and (f) a comparison of MSEs for all adjusted estimators.

Fig. S25: Coverage: stylized scenario, large effect ($\alpha_1 = 2$)

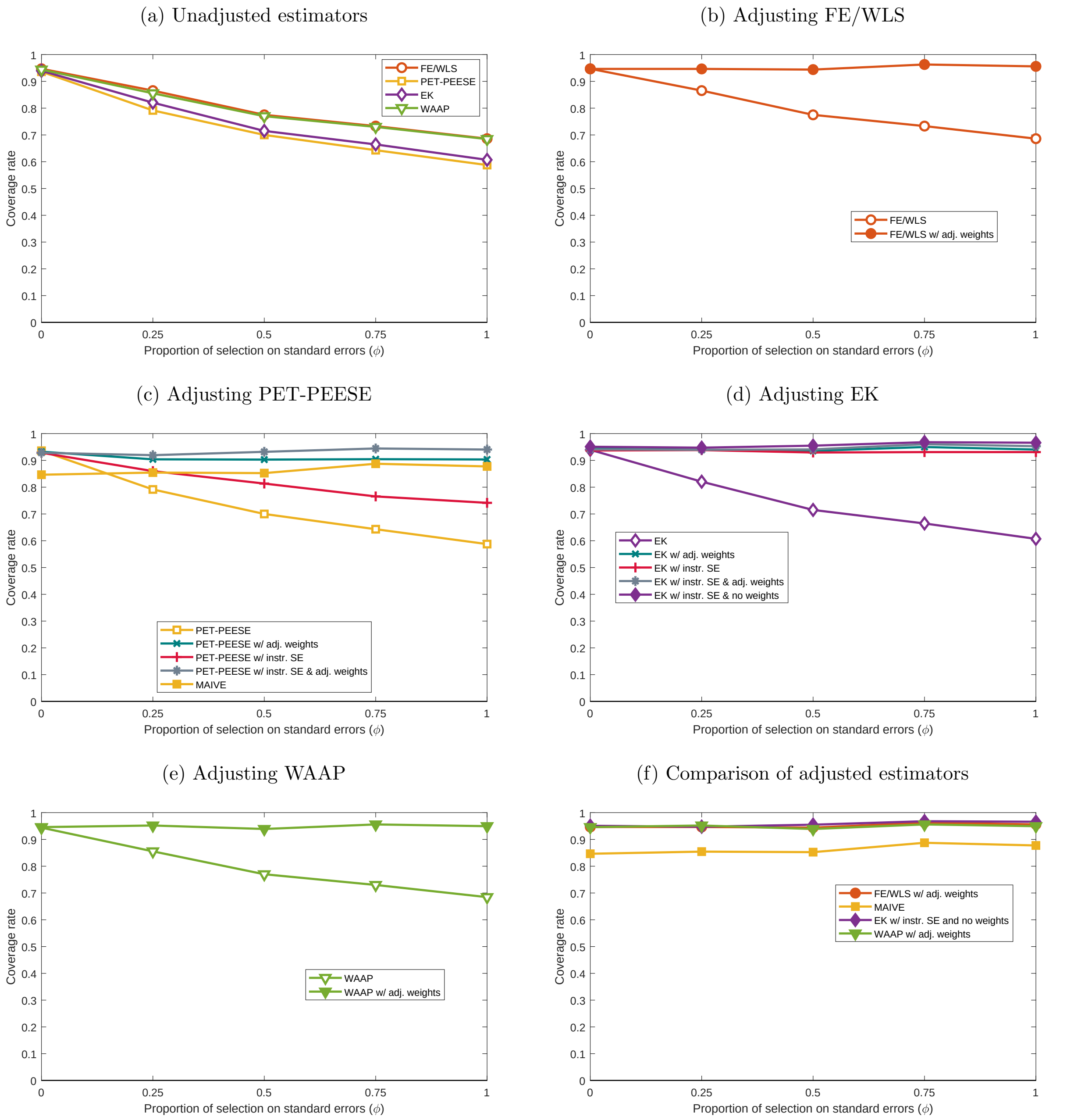

Notes: Figure shows the coverage rate for the case of a large effect in our stylized scenario. Panels show (a) a comparison of coverage rates for all unadjusted estimators; coverage rate for (b) the fixed effects or weighted least squares estimator with adjustment, (c) the adjusted precision-effect test and precision-effect estimate with standard errors, (d) the adjusted endogenous kink estimator, (e) the adjusted weighted average of adequately powered; and (f) a comparison of coverage rates for all adjusted estimators.

S6 Mean Squared Error (MSE) Simulation Results

Fig. S26: MSE: stylized scenario, no effect ($\alpha_1 = 0$)

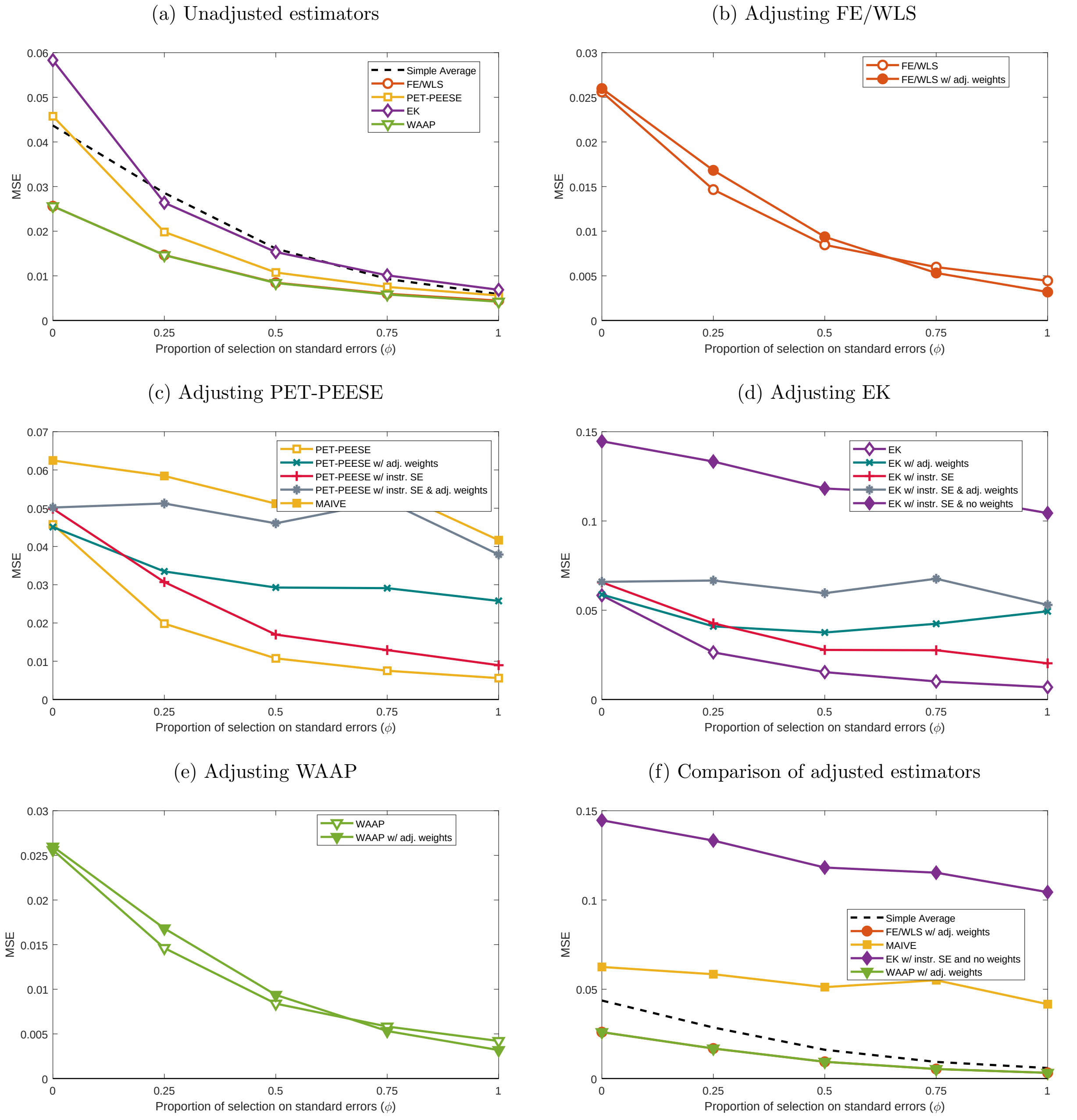

Notes: Figure shows the mean squared error (MSE) for the case of no effect in our stylized scenario. Panels show (a) a comparison of MSEs for all unadjusted estimators; MSE for (b) the fixed effects or weighted least squares estimator with adjustment, (c) the adjusted precision-effect test and precision-effect estimate with standard errors, (d) the adjusted endogenous kink estimator, (e) the adjusted weighted average of adequately powered; and (f) a comparison of MSEs for all adjusted estimators.

Fig. S27: MSE: stylized scenario, moderate effect ($\alpha_1 = 1$)

(a) Unadjusted estimators

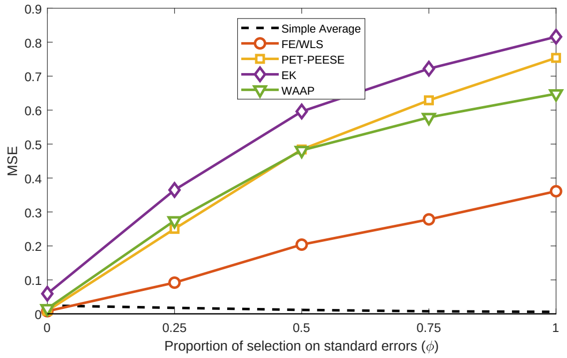

(b) Adjusting FE/WLS

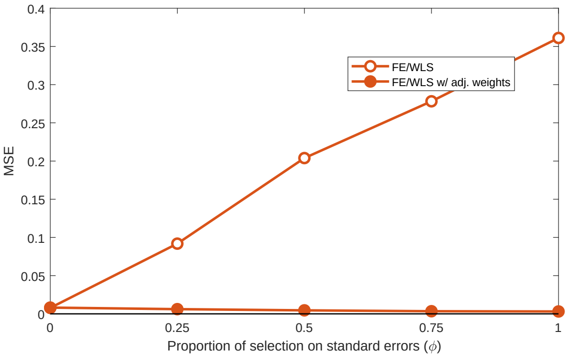

(c) Adjusting PET-PEESE

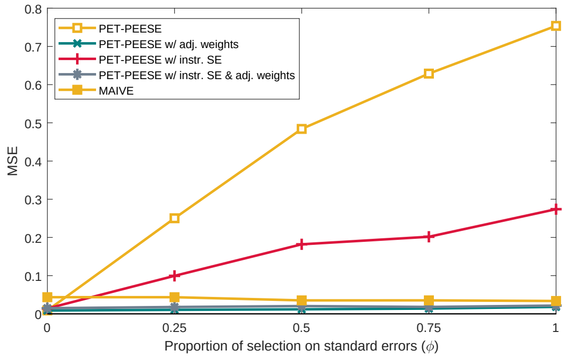

(d) Adjusting EK

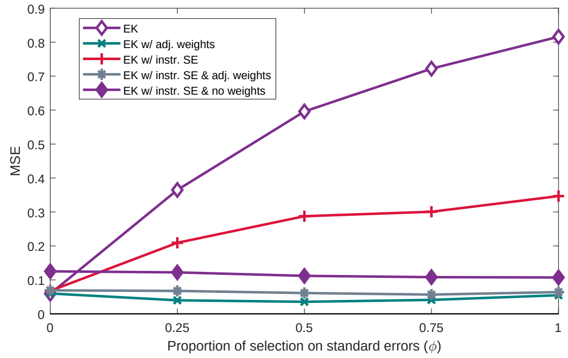

(e) Adjusting WAAP

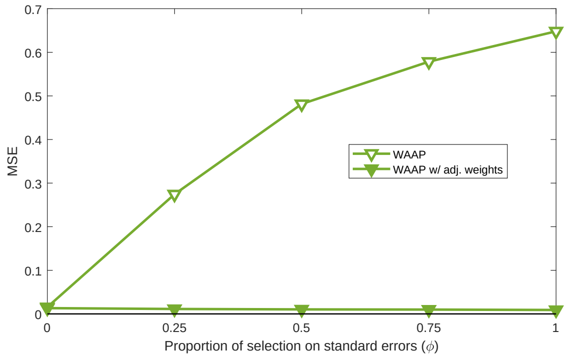

(f) Comparison of adjusted estimators

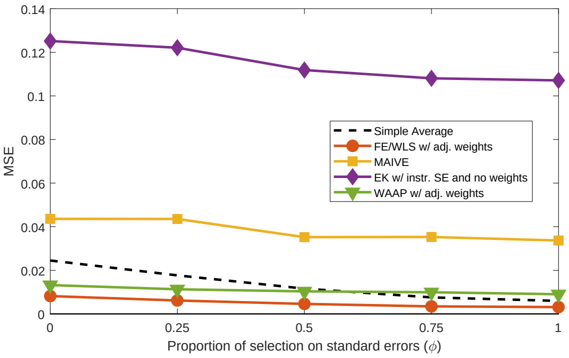

Notes: Figure shows the mean squared error (MSE) for the case of a moderate effect in our stylized scenario. Panels show (a) a comparison of MSEs for all unadjusted estimators; MSE for (b) the fixed effects or weighted least squares estimator with adjustment, (c) the adjusted precision-effect test and precision-effect estimate with standard errors, (d) the adjusted endogenous kink estimator, (e) the adjusted weighted average of adequately powered; and (f) a comparison of MSEs for all adjusted estimators.

Fig. S28: MSE: p-hacking selection, no effect ($\alpha_1 = 0$), various values of ψ

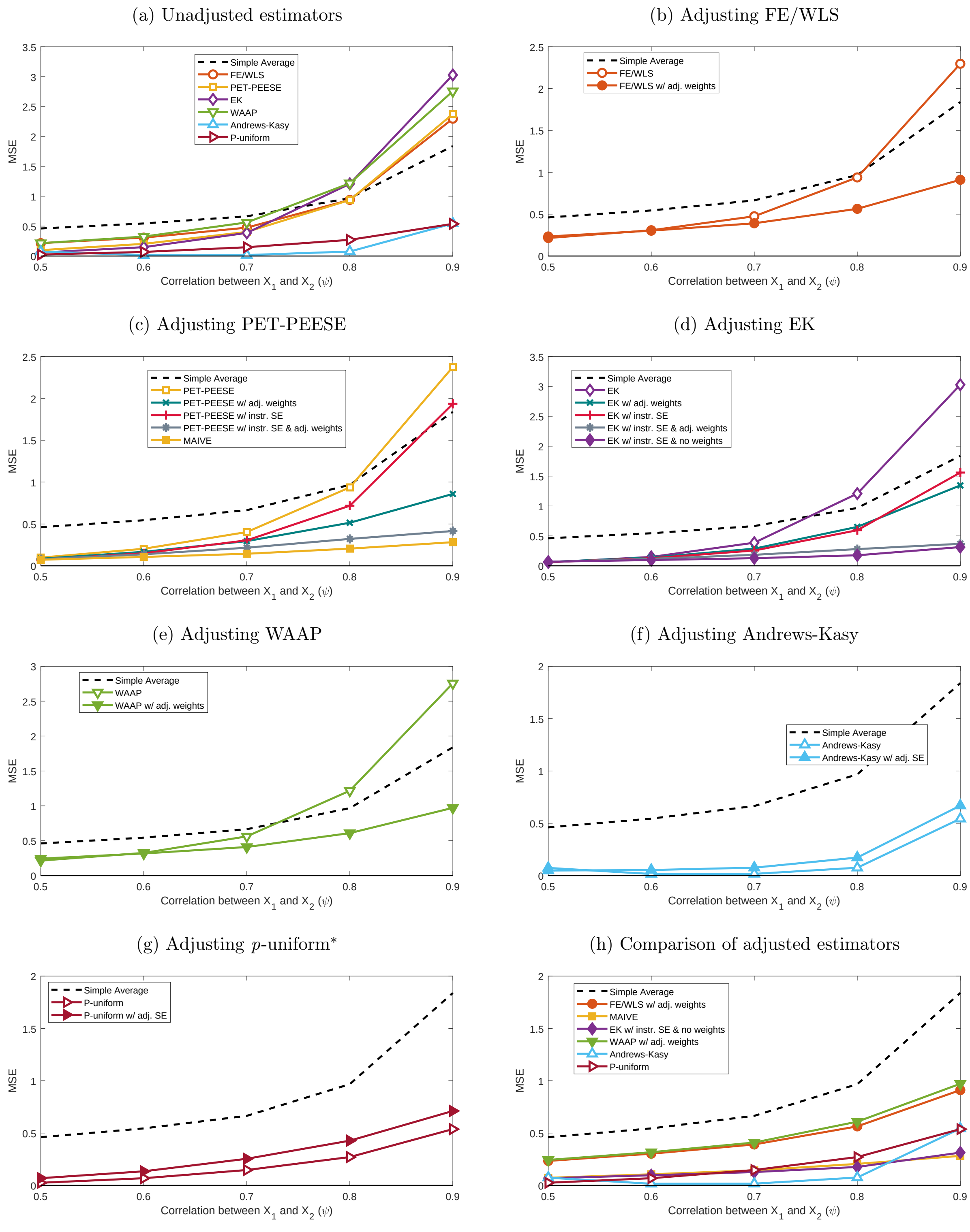

Notes: Figure shows the mean squared error (MSE) for the case of no effect in the p-hacking scenario. Panels show (a) a comparison of MSEs for all unadjusted estimators; MSE for (b) the fixed effects or weighted least squares estimator with adjustment, (c) the adjusted precision-effect test and precision-effect estimate with standard errors, (d) the adjusted endogenous kink estimator, (e) the adjusted weighted average of adequately powered; (f) the adjusted Andrews and Kasy estimator, (g) the adjusted p-uniform* method; and (h) a comparison of MSEs for all adjusted estimators.

Fig. S29: MSE: p-hacking selection, positive effect ($\alpha_1 = 1$), various values of ψ

(a) Unadjusted estimators

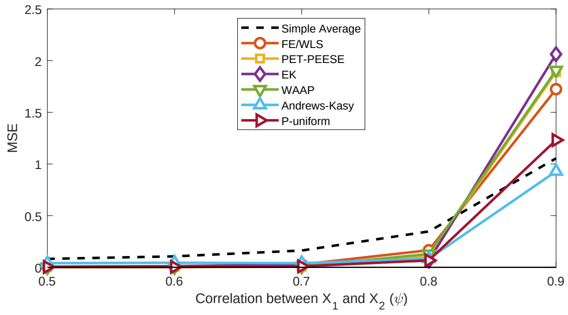

(b) Adjusting FE/WLS

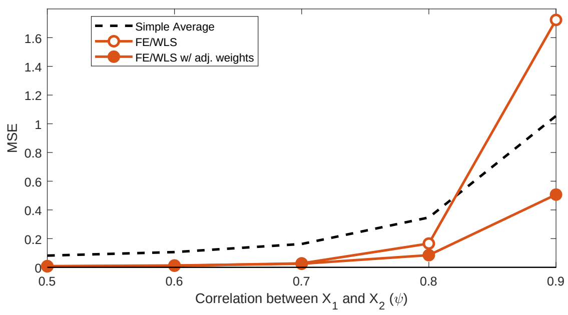

(c) Adjusting PET-PEESE

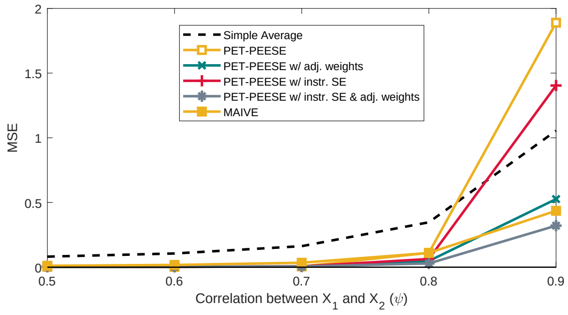

(d) Adjusting EK

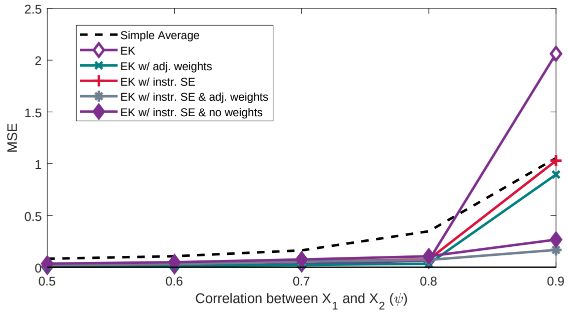

(e) Adjusting WAAP

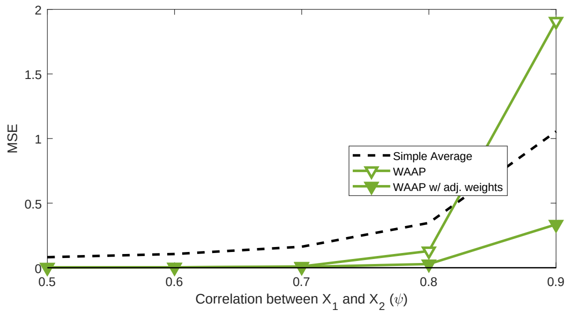

(f) Adjusting Andrews-Kasy

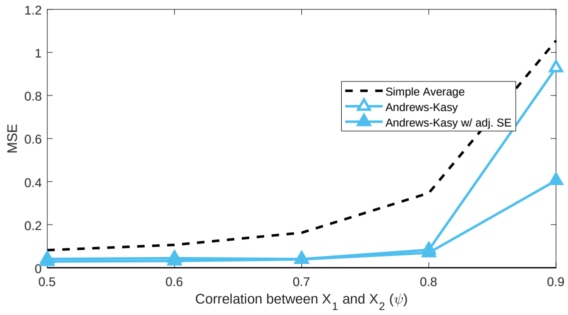

(g) Adjusting p-uniform*

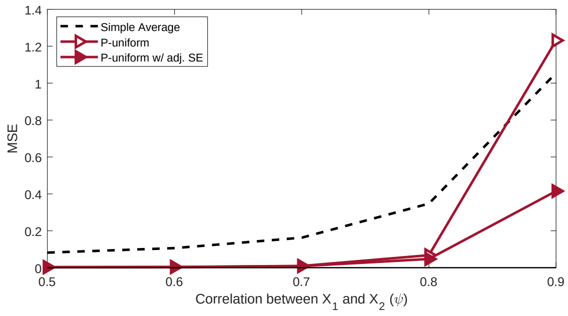

(h) Comparison of adjusted estimators

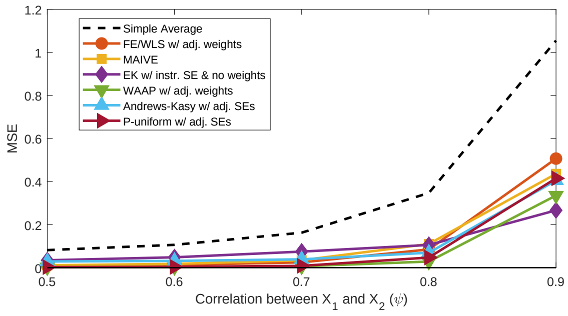

Notes: Figure shows the mean squared error (MSE) for the case of a positive effect in the p-hacking scenario. Panels show (a) a comparison of MSEs for all unadjusted estimators; MSE for (b) the fixed effects or weighted least squares estimator with adjustment, (c) the adjusted precision-effect test and precision-effect estimate with standard errors, (d) the adjusted endogenous kink estimator, (e) the adjusted weighted average of adequately powered; (f) the adjusted Andrews and Kasy estimator, (g) the adjusted p-uniform* method; and (h) a comparison of MSEs for all adjusted estimators.

References

1. Iyengar S, Greenhouse JB. Selection Models and the File Drawer Problem. *Statistical Science* 1988; 3(1): 109–117.
2. Hedges LV. Modeling Publication Selection Effects in Meta-Analysis. *Statistical Science* 1992; 72(2): 246–255.
3. Pustejovsky JE, Rodgers MA. Testing for funnel plot asymmetry of standardized mean differences. *Research Synthesis Methods* 2019; 10(1): 57–71.
4. Rodgers MA, Pustejovsky JE. Evaluating meta-analytic methods to detect selective reporting in the presence of dependent effect sizes. *Psychological Methods* 2021; 26(2): 141–160.
5. Andrews I, Kasy M. Identification of and correction for publication bias. *American Economic Review* 2019; 109(8): 2766–2794.
6. Kvarven A, Stromland E, Johannesson M. Identification of and Correction for Publication Bias: Comment. MetaArXiv dh87m, Center for Open Science, doi: 10.31219/osf.io/dh87m; 2019.
7. Hong S, Reed WR. Using Monte Carlo experiments to select meta-analytic estimators. *Research Synthesis Methods* 2021; 12(2): 192–215.
8. Mathur MB, VanderWeele TJ. Sensitivity analysis for publication bias in meta-analyses. *Journal of the Royal Statistical Society Series C* 2020; 69(5): 10911119.
9. Mathur MB. Sensitivity analysis for p-hacking in meta-analyses. Quantitative Sciences Unit and Department of Pediatrics, Stanford University, working paper, doi: 10.31219/osf.io/ezjsx; 2022.
10. Mathur MB. P-hacking in meta-analyses: A formalization and new meta-analytic methods. *Research Synthesis Methods* 2024; 15(3): 483-499.
11. Schmidt FL, Oh IS, Hayes TL. Fixed-versus random-effects models in meta-analysis: Model properties and an empirical comparison of differences in results. *British Journal of Mathematical and Statistical Psychology* 2009; 62(1): 97–128.
12. Stanley TD, Doucouliagos H, Ioannidis JPA. Beyond Random Effects: When Small-Study Findings Are More Heterogeneous. *Advances in Methods and Practices in Psychological Science* 2022; 5(4): 1–11.
13. Stanley TD, Doucouliagos H. Neither fixed nor random: Weighted least squares meta-analysis. *Statistics in Medicine* 2015; 34(13): 2116–2127.
14. Stanley TD, Doucouliagos H. Neither fixed nor random: Weighted least squares meta-regression. *Research Synthesis Methods* 2017; 8(1): 19–42.
15. Stanley TD, Doucouliagos H. Harnessing the power of excess statistical significance: Weighted and iterative least squares. *Psychological Methods* 2024; 29(2): 407-420.
16. Kvarven A, Stromland E, Johannesson M. Comparing meta-analyses and preregistered multiple-laboratory replication projects. *Nature Human Behavior* 2020; 4: 423–434.
17. Copas JB, Li HG. Inference for Non-random Samples. *Journal of the Royal Statistical Society: Series B (Statistical Methodology)* 1997; 59(1): 55–95.
18. Copas JB. What works? Selectivity models and meta-analysis. *Journal of the Royal Statistical Society: Series A (Statistics in Society)* 1999; 162(1): 95–109.
19. Copas JB, Shi JQ. A sensitivity analysis for publication bias in systematic reviews. *Statistical Methods in Medical Research* 2001; 10(4): 251–265.
20. Copas JB. A likelihood-based sensitivity analysis for publication bias in meta-analysis. *Journal of the Royal Statistical Society: Series C (Applied Statistics)* 2013; 62(1): 47–66.
21. McShane BB, Böckenholt U, Hansen KT. Adjusting for Publication Bias in Meta-Analysis: An Evaluation of Selection Methods and Some Cautionary Notes. *Perspectives on Psychological Science* 2016; 11(5): 730–749.
22. Stanley TD. Beyond Publication Bias. *Journal of Economic Surveys* 2005; 19(3): 309–345.
23. Hausman J. Mismeasured Variables in Econometric Analysis: Problems from the Right and Problems from the Left. *Journal of Economic Perspectives* 2001; 15(4): 57–67.
24. Havranek T, Irsova Z, Laslopova L, Zeynalova O. Publication and Attenuation Biases in Measuring Skill Substitution. *The Review of Economics and Statistics* 2024; 106(5): 1187-1200. doi: 10.1162/rest_a_01227.

25. Card D, Krueger AB. Time-series minimum-wage studies: A meta-analysis. *American Economic Review* 1995; 85(2): 238–243.

26. Bom PRD, Rachinger H. A kinked meta-regression model for publication bias correction. *Research Synthesis Methods* 2019; 10(4): 497–514.

27. Olken BA. Promises and Perils of Pre-analysis Plans. *Journal of Economic Perspectives* 2015; 29(3): 61–80.

28. Bom PRD, Ligthart JE. What Have We Learned From Three Decades of Research on the Productivity of Public Capital?. *Journal of Economic Surveys* 2014; 28(5): 889–916.

29. Havranek T. Measuring intertemporal substitution: The importance of method choices and selective reporting. *Journal of the European Economic Association* 2015; 13(6): 1180–1204.

30. Egger M, Schneider M, Smith GD. Spurious precision? Meta-analysis of observational studies. *BMJ* 1998; 316(7125): 140–144.

31. Hansen TF. On bias and precision in meta-analysis: the error in the error. *Journal of Evolutionary Biology* 2016; 29(10): 1919–1921.

32. Nakagawa S, Lagisz M, Jennions MD, et al. Methods for testing publication bias in ecological and evolutionary meta-analyses. *Methods in Ecology and Evolution* 2022; 13(1): 4–21.

33. Nakagawa S, Noble DWA, Lagisz M, Spake R, Viechtbauer W, Senior AM. A robust and readily implementable method for the meta-analysis of response ratios with and without missing standard deviations. *Ecology Letters* 2023; 26(2): 232–244.

34. Hedges LV. Effect Sizes in Cluster-Randomized Designs. *Journal of Educational and Behavioral Statistics* 2007; 32(4): 341–370.

35. Hedges LV. Effect Sizes in Three-Level Cluster-Randomized Experiments. *Journal of Educational and Behavioral Statistics* 2011; 36(3): 346–380.

36. Hedges LV, Citkowicz M. Estimating effect size when there is clustering in one treatment group. *Behavior Research Methods* 2015; 47: 1295-1308.

37. Sanchez-Meca J, Marín-Martínez F. Weighting by Inverse Variance or by Sample Size in Meta-Analysis: A Simulation Study. *Educational and Psychological Measurement* 1998; 58(2): 211–220.

38. Deeks JJ, Macaskill P, Irwig L. The performance of tests of publication bias and other sample size effects in systematic reviews. *Journal of Clinical Epidemiology* 2005; 58(9): 882–893.

39. Peters JL, Sutton AJ, Jones DR, Abrams KR, Rushton L. Comparison of Two Methods to Detect Publication Bias in Meta-analysis. *JAMA* 2006; 295(6): 676-680.

40. Hedges LV. A random effects model for effect sizes. *Psychological Bulletin* 1983; 93(2): 388–395.

41. Sterne JA, Egger M. Funnel plots for detecting bias in meta-analysis: guidelines on choice of axis. *Journal of Clinical Epidemiology* 2001; 54(10): 1046–1055.

42. Andrews I. Conditional Linear Combination Tests for Weakly Identified Models. *Econometrica* 2016; 84(6): 2155–2182.

43. Andrews I. Valid Two-Step Identification-Robust Confidence Sets for GMM. *The Review of Economics and Statistics* 2018; 100(2): 337–348.

44. Sun L. Implementing valid two-step identification-robust confidence sets for linear instrumental-variables models. *Stata Journal* 2018; 18(4): 803–825.

45. Andrews I, Stock JH, Sun L. Weak Instruments in Instrumental Variables Regression: Theory and Practice. *Annual Review of Economics* 2019; 11(1): 727–753.

46. Fraser H, Parker T, Nakagawa S, Barnett A, Fidler F. Questionable research practices in ecology and evolution. *PloS ONE* 2018; 13(7): e0200303.

47. Krueger AB. Experimental Estimates of Education Production Functions. *The Quarterly Journal of Economics* 1999; 114(2): 497-532.

48. Stock JH, Watson MW. *star_sw subset of webstar database*. Instructional dataset, accompanying Introduction to Econometrics, J.H. Stock and M.W. Watson, Pearson Education . 2003.

49. Stanley TD, Doucouliagos H. Meta-regression approximations to reduce publication selection bias. *Research Synthesis Methods* 2014; 5(1): 60–78.

50. Bom PRD, Rachinger H. A generalized-weights solution to sample overlap in meta-analysis. *Research Synthesis Methods* 2020; 11(6): 812–832.

51. Joshi M, Pustejovsky JE, Beretvas SN. Cluster wild bootstrapping to handle dependent effect sizes in meta-analysis with a small number of studies. *Research Synthesis Methods* 2022; 13(4): 457–477.

52. Pustejovsky JE, Tipton E. Meta-Analysis with Robust Variance Estimation: Expanding the Range of Working Models. MetaArXiv vyfcj, Center for Open Science, doi: 10.31219/osf.io/vyfcj; 2020.

53. Vembye M, Pustejovsky J, Pigott T. Power Approximations for Overall Average Effects in Meta-Analysis With Dependent Effect Sizes. *Journal of Educational and Behavioral Statistics* 2023; 48(1): 70–102.

54. Ioannidis JP, Stanley TD, Doucouliagos H. The Power of Bias in Economics Research. *The Economic Journal* 2017; 127(605): F236–F265.

55. Hausman JA. Specification Tests in Econometrics. *Econometrica* 1978; 46(6): 1251–1271.

56. Abadie A, Athey S, Imbens GW, Wooldridge JM. When Should You Adjust Standard Errors for Clustering?. *The Quarterly Journal of Economics* 2023; 138(1): 1-35.

57. de Chaisemartin C, Ramirez-Cuellar J. At What Level Should One Cluster Standard Errors in Paired and Small-Strata Experiments?. *American Economic Journal: Applied Economics* 2024; 16(1): 193-212.

58. MacKinnon JG, Nielsen MO, Webb MD. Cluster-robust inference: A guide to empirical practice. *Journal of Econometrics* 2023; 232(2): 272-299.

59. Chesher A, Jewitt I. The Bias of a Heteroskedasticity Consistent Covariance Matrix Estimator. *Econometrica* 1987; 55(5): 1217-1222.

60. Lang K. How Credible is the Credibility Revolution?. *Journal of Labor Economics* 2025(forthcoming).

61. Tipton E. Small sample adjustments for robust variance estimation with meta-regression. *Psychological Methods* 2015; 20(3): 375–393.

62. Tipton E, Pustejovsky JE. Small-Sample Adjustments for Tests of Moderators and Model Fit Using Robust Variance Estimation in Meta-Regression. *Journal of Educational and Behavioral Statistics* 2015; 40(6): 604–634.

63. Pustejovsky JE, Tipton E. Small-Sample Methods for Cluster-Robust Variance Estimation and Hypothesis Testing in Fixed Effects Models. *Journal of Business & Economic Statistics* 2018; 36(4): 672–683.

64. Pustejovsky JE, Tipton E. Meta-analysis with Robust Variance Estimation: Expanding the Range of Working Models. *Prevention Science* 2022; 23: 425–438.

65. Pustejovsky JE, Tipton E. Corrigendum: Small Sample Methods for Cluster-Robust Variance Estimation and Hypothesis Testing in Fixed Effects Models. *Journal of Business & Economic Statistics* 2023; 41(2): 650–652.

66. Fisher Z, Tipton E. robumeta: An R-package for robust variance estimation in meta-analysis. https://cran.r-project.org/web/packages/robumeta/vignettes/robumetaVignette.pdf; 2015.

67. Roodman D, Nielsen MØ, MacKinnon JG, Webb MD. Fast and Wild: Bootstrap Inference in Stata Using Boottest. *The Stata Journal* 2019; 19(1): 4–60.

68. Brodeur A, Carrell S, Figlio D, Lusher L. Unpacking p-hacking and publication bias. *American Economic Review* 2023; 113(11): 2974–3002.

69. Bruns SB, Ioannidis JP. p-Curve and p-Hacking in Observational Research. *PloS ONE* 2016; 11(2): e0149144.

70. Bruns SB. Meta-Regression Models and Observational Research. *Oxford Bulletin of Economics and Statistics* 2017; 79(5): 637–653.

71. Simonsohn U, Simmons J, Nelson LD. Above Averaging in Literature Reviews. *Nature Reviews Psychology* 2022; 1: 551–552.

72. Gechert S, Havranek T, Irsova Z, Kolcunova D. Measuring Capital-Labor Substitution: The Importance of Method Choices and Publication Bias. *Review of Economic Dynamics* 2022; 45(C): 55–82.

73. Imai T, Rutter TA, Camerer CF. Meta-Analysis of Present-Bias Estimation Using Convex Time Budgets. *The Economic Journal* 2021; 131(636): 1788–1814.

74. Gechert S, Heimberger P. Do corporate tax cuts boost economic growth?. *European Economic Review* 2022; 147(C): 104157.

75. Matousek J, Havranek T, Irsova Z. Individual discount rates: A meta-analysis of experimental evidence. *Experimental Economics* 2022; 25(1): 318–358.

76. Keane M, Neal T. Instrument strength in IV estimation and inference: A guide to theory and practice. *Journal of Econometrics* 2023; 235(2): 1625–1653.

77. Srull TK, Wyer RS. The role of category accessibility in the interpretation of information about persons: Some determinants and implications. *Journal of Personality* 1979; 37(10): 1660–1672.

78. Graham J, Haidt J, Nosek BA. Liberals and conservatives rely on different sets of moral foundations. *Journal of Personality Social Psychology* 2009; 96(5): 1029–1046.

79. Monin B, Miller DT. Moral credentials and the expression of prejudice. *Journal of Personality and Social Psychology* 2001; 81(1): 33–43.

80. Sripada C, Kessler D, Jonides J. Methylphenidate blocks effort-induced depletion of regulatory control in healthy volunteers. *Psychological Science* 2014; 25(6): 1227–1234.

81. Tversky A, Kahneman D. The framing of decisions and the psychology of choice. *Science* 1981; 211(4481): 453–458.

82. Oppenheimer DM, Meyvis T, Davidenko N. Instructional manipulation checks: Detecting satisficing to increase statistical power. *Journal of Experimental Social Psychology* 2009; 45(4): 867–872.

83. Rand DG, Greene JD, Nowak MA. Spontaneous giving and calculated greed. *Nature* 2012; 489: 427–430.

84. Husnu S, Crisp RJ. Elaboration enhances the imagined contact effect. *Journal of Experimental Social Psychology* 2010; 46(6): 943–950.

85. Schwarz N, Strack F, Mai HP. Assimilation and contrast effects in part-whole question sequences: A conversational logic analysis. *Public Opinion Quarterly* 1991; 55(1): 3–23.

86. Schooler JW, Engstler-Schooler TY. Verbal overshadowing of visual memories: Some things are better left unsaid. *Cognitive Psychology* 1990; 22(1): 36–71.

87. Mazar N, Amir O, Ariely D. The dishonesty of honest people: A theory of self-concept maintenance. *Journal of Marketing Research* 2008; 45(6): 633–644.

88. Critcher CR, Gilovich T. Incidental environmental anchors. *Journal of Behavioral Decision Making* 2008; 21(3): 241–251.

89. Jostmann NB, Lakens D, Schubert TW. Weight as an embodiment of importance. *Psychological Science* 2009; 20(9): 1169–1174.

90. Strack F, Martin LL, Stepper S. Inhibiting and facilitating conditions of the human smile: A nonobtrusive test of the facial feedback hypothesis. *Journal of Personality and Social Psychology* 1988; 54(5): 768–777.

91. Hauser M, Cushman F, Young L, Kang-Xing Jin R, Mikhail J. A dissociation between moral judgments and justifications. *Mind Language* 2007; 22(1): 1–21.

92. Bartos F, Maier M, Wagenmakers EJ, et al. Footprint of publication selection bias on meta-analyses in medicine, environmental sciences, psychology, and economics. *Research Synthesis Methods* 2024; 15(3): 500-511.
