# Supplementary material for: Spurious precision in meta-analysis of observational research
Source: Nat Commun. 2025 Sep 26;16:8454. doi: 10.1038/s41467-025-63261-0 (PMC12475282; doi:10.1038/s41467-025-63261-0)
Supplement: Supplementary file 2 — Reporting Summary [file 41467_2025_63261_MOESM2_ESM.pdf]

nature portfolio

Corresponding author(s): Zuzana Irsova

Last updated by author(s): May 13, 2025

Reporting Summary

Nature Portfolio wishes to improve the reproducibility of the work that we publish. This form provides structure for consistency and transparency in reporting. For further information on Nature Portfolio policies, see our Editorial Policies and the Editorial Policy Checklist.

Statistics

For all statistical analyses, confirm that the following items are present in the figure legend, table legend, main text, or Methods section.

n/a	Confirmed	
☐	☒	The exact sample size (*n*) for each experimental group/condition, given as a discrete number and unit of measurement
☒	☐	A statement on whether measurements were taken from distinct samples or whether the same sample was measured repeatedly
☐	☒	The statistical test(s) used AND whether they are one- or two-sided<br>*Only common tests should be described solely by name; describe more complex techniques in the Methods section.*
☐	☒	A description of all covariates tested
☐	☒	A description of any assumptions or corrections, such as tests of normality and adjustment for multiple comparisons
☐	☒	A full description of the statistical parameters including central tendency (e.g. means) or other basic estimates (e.g. regression coefficient) AND variation (e.g. standard deviation) or associated estimates of uncertainty (e.g. confidence intervals)
☐	☒	For null hypothesis testing, the test statistic (e.g. *F*, *t*, *r*) with confidence intervals, effect sizes, degrees of freedom and *P* value noted<br>*Give P values as exact values whenever suitable.*
☒	☐	For Bayesian analysis, information on the choice of priors and Markov chain Monte Carlo settings
☒	☐	For hierarchical and complex designs, identification of the appropriate level for tests and full reporting of outcomes
☐	☒	Estimates of effect sizes (e.g. Cohen's *d*, Pearson's *r*), indicating how they were calculated

Our web collection on statistics for biologists contains articles on many of the points above.

Software and code

Policy information about availability of computer code

Data collection	All data used in this study have been deposited in the meta-analysis.cz database at meta-analysis.cz/maive and archived at doi.org/10.5281/zenodo.15425605. These are raw data.
Data analysis	All codes used in this study have been deposited in the GitHub database at github.com/meta-analysis-es/maive and archived at doi.org/10.5281/zenodo.15425605. The maive R package and an interactive web application are available at spuriousprecision.com.

For manuscripts utilizing custom algorithms or software that are central to the research but not yet described in published literature, software must be made available to editors and reviewers. We strongly encourage code deposition in a community repository (e.g. GitHub). See the Nature Portfolio guidelines for submitting code & software for further information.

Data

Policy information about availability of data

All manuscripts must include a data availability statement. This statement should provide the following information, where applicable:

- Accession codes, unique identifiers, or web links for publicly available datasets
- A description of any restrictions on data availability
- For clinical datasets or third party data, please ensure that the statement adheres to our policy

All replication files are publicly available at meta-analysis.cz/maive/maive_replication.zip.

Research involving human participants, their data, or biological material

Policy information about studies with human participants or human data. See also policy information about sex, gender (identity/presentation), and sexual orientation and race, ethnicity and racism.

Reporting on sex and gender	No reporting on sex and gender.
Reporting on race, ethnicity, or other socially relevant groupings	No reporting on race.
Population characteristics	No human participants.
Recruitment	No recruitment.
Ethics oversight	No needed due to the lack of human participants.

Note that full information on the approval of the study protocol must also be provided in the manuscript.

Field-specific reporting

Please select the one below that is the best fit for your research. If you are not sure, read the appropriate sections before making your selection.

☐ Life sciences ☒ Behavioural & social sciences ☐ Ecological, evolutionary & environmental sciences

For a reference copy of the document with all sections, see nature.com/documents/nr-reporting-summary-flat.pdf

Behavioural & social sciences study design

All studies must disclose on these points even when the disclosure is negative.

Study description	The study is a Monte Carlo analysis combined with a meta-meta-analysis (analysis of existing meta-analysis data).
Research sample	Results of previous meta-analyses and replications reported by Kvarven et al., 2020, and Bartos et al., 2024.
Sampling strategy	No sampling strategy needed due to the use of existing datasets.
Data collection	No data collection needed due to the use of existing datasets.
Timing	No timing applicable due to the use of existing datasets.
Data exclusions	Not applicable due to the use of existing datasets.
Non-participation	Not applicable due to the use of existing datasets.
Randomization	Not applicable due to the use of existing datasets.

Reporting for specific materials, systems and methods

We require information from authors about some types of materials, experimental systems and methods used in many studies. Here, indicate whether each material, system or method listed is relevant to your study. If you are not sure if a list item applies to your research, read the appropriate section before selecting a response.

Materials & experimental systems

n/a	Involved in the study
☒	☐ Antibodies
☒	☐ Eukaryotic cell lines
☒	☐ Palaeontology and archaeology
☒	☐ Animals and other organisms
☒	☐ Clinical data
☒	☐ Dual use research of concern
☒	☐ Plants

Methods

n/a	Involved in the study
☒	☐ ChIP-seq
☒	☐ Flow cytometry
☒	☐ MRI-based neuroimaging

Plants

Seed stocks	NA
Novel plant genotypes	NA
Authentication	NA
